# Supplementary material for: Haplotype Purging after Relaxation of Selection in Lines of Chickens That Had Undergone Long-Term Selection for High and Low Body Weight
Source: Genes (Basel). 2020 Jun 8;11(6):630. doi: 10.3390/genes11060630 (PMC7349872; doi:10.3390/genes11060630)
Supplement: Supplementary file 1 [file genes-11-00630-s001.zip › SupplementaryTablesS1-S12.pdf]

Table S1. Number of individuals with genotypes and phenotypes used in the Virginia line AIL for haplotype association analyses.

Generation	Number of Individuals
F_2	665
F_3	383
F_4	110
F_5	115
F_6	88
F_7	46
F_8	254
F_9	50
F_{10}	60
F_{11}	59
F_{13}	67
F_{14}	88
F_{15}	682

Table S2. SNPs with allele-frequency difference > 0.5 in purging region in LW lineage on chromosome 1 (177438078-177438879 bp).

Chromosome	Position	AFD
1	177438813	0,558
1	177438879	0,584

Table S3. SNPs with allele-frequency difference > 0.5 in purging region in LW lineage on chromosome 6 (16.9-17.7 Mb). Loci highlighted in green were tag SNPs selected by backward-elimination analysis in this region.

Chromosome	Position	AFD
6	16936608	0,526
6	16936719	0,500
6	16936837	0,526
6	16937663	0,500
6	16938360	0,526
6	16939131	0,526
6	16939200	0,526
6	16940356	0,500
6	16940400	0,526
6	16940652	0,526
6	16947247	0,526
6	16948206	0,526
6	16948626	0,500
6	16949032	0,526
6	16950126	0,500
6	16951424	0,526
6	16951791	0,526
6	16952171	0,526
6	16958434	0,526
6	16959791	0,526
6	16959806	0,526
6	16960895	0,500
6	17522977	0,579
6	17523004	0,579
6	17523046	0,579
6	17524185	0,579
6	17524550	0,579
6	17524606	0,579
6	17525074	0,579
6	17525086	0,579
6	17525191	0,579
6	17525323	0,579
6	17525409	0,579
6	17525441	0,579
6	17525450	0,579
6	17525632	0,579
6	17525664	0,579
6	17525774	0,579
6	17526620	0,579
6	17526670	0,579
6	17527201	0,579
6	17527240	0,579
6	17527280	0,579

6	17527566	0,579
6	17528573	0,579
6	17528729	0,579
6	17528884	0,579
6	17528928	0,579
6	17528959	0,579
6	17529607	0,579
6	17529609	0,579
6	17529644	0,579
6	17529667	0,579
6	17529764	0,579
6	17530052	0,579
6	17530251	0,579
6	17530252	0,579
6	17530975	0,579
6	17531356	0,579
6	17531439	0,579
6	17531442	0,579
6	17531522	0,579
6	17531528	0,579
6	17531555	0,579
6	17531572	0,579
6	17531575	0,579
6	17531577	0,579
6	17531617	0,579
6	17531640	0,579
6	17531706	0,579
6	17531717	0,579
6	17531722	0,579
6	17532002	0,579
6	17532185	0,579
6	17532313	0,579
6	17532361	0,579
6	17532460	0,579
6	17532468	0,579
6	17532697	0,579
6	17532742	0,579
6	17532744	0,579
6	17532998	0,579
6	17533439	0,579
6	17533475	0,579
6	17533661	0,579
6	17534337	0,579
6	17534400	0,579
6	17534416	0,579
6	17534444	0,579
6	17534497	0,579
6	17534501	0,579

6	17534605	0,579
6	17534631	0,579
6	17534663	0,579
6	17534670	0,579
6	17534683	0,579
6	17534707	0,579
6	17534718	0,579
6	17534767	0,579
6	17534781	0,579
6	17534811	0,579
6	17535010	0,579
6	17535090	0,579
6	17535356	0,579
6	17535842	0,579
6	17536248	0,579
6	17536444	0,579
6	17536546	0,579
6	17536736	0,579
6	17538172	0,579
6	17538618	0,579
6	17539125	0,579
6	17539189	0,579
6	17539197	0,579
6	17539240	0,579
6	17539271	0,579
6	17539344	0,579
6	17539411	0,579
6	17539429	0,579
6	17539462	0,579
6	17539832	0,579
6	17540206	0,579
6	17540418	0,579
6	17540458	0,579
6	17540779	0,579
6	17540788	0,579
6	17540796	0,579
6	17540802	0,579
6	17540807	0,579
6	17540843	0,579
6	17540922	0,579
6	17541527	0,579
6	17541745	0,579
6	17542383	0,579
6	17542393	0,579
6	17542403	0,579
6	17542440	0,579
6	17542506	0,579
6	17544709	0,579

6	17544818	0,579
6	17545262	0,579
6	17545346	0,579
6	17545387	0,579
6	17545471	0,579
6	17545686	0,579
6	17545767	0,579
6	17545835	0,579
6	17545859	0,579
6	17546460	0,579
6	17546484	0,579
6	17546499	0,579
6	17546500	0,579
6	17546656	0,579
6	17546677	0,579
6	17546828	0,579
6	17547151	0,579
6	17547223	0,579
6	17547537	0,579
6	17547544	0,579
6	17547651	0,579
6	17547717	0,579
6	17547718	0,579
6	17547744	0,579
6	17547755	0,579
6	17547937	0,579
6	17547979	0,579
6	17548076	0,579
6	17548130	0,579
6	17548205	0,579
6	17548508	0,579
6	17548517	0,579
6	17548522	0,579
6	17548526	0,579
6	17548580	0,579
6	17548599	0,579
6	17548641	0,579
6	17549016	0,579
6	17549154	0,579
6	17549181	0,579
6	17549185	0,579
6	17549273	0,579
6	17549278	0,579
6	17549319	0,579
6	17549353	0,579
6	17549360	0,579
6	17549386	0,579
6	17549389	0,579

6	17549471	0,579
6	17549475	0,579
6	17549476	0,579
6	17549477	0,579
6	17549527	0,579
6	17549947	0,579
6	17550114	0,579
6	17550352	0,579
6	17550462	0,579
6	17550688	0,579
6	17550819	0,579
6	17551541	0,579
6	17551690	0,579
6	17551716	0,579
6	17551760	0,579
6	17551834	0,579
6	17551852	0,579
6	17551946	0,579
6	17552005	0,579
6	17552828	0,579
6	17552829	0,579
6	17552858	0,579
6	17553080	0,579
6	17553104	0,579
6	17553332	0,579
6	17553855	0,579
6	17553859	0,579
6	17554058	0,579
6	17556289	0,579
6	17557133	0,579
6	17557346	0,579
6	17559515	0,579
6	17559687	0,579
6	17560297	0,579
6	17560854	0,579
6	17561418	0,579
6	17561463	0,579
6	17561656	0,579
6	17561657	0,579
6	17561857	0,579
6	17561869	0,579
6	17561875	0,579
6	17561880	0,579
6	17561899	0,579
6	17561912	0,579
6	17562143	0,579
6	17562148	0,579
6	17562152	0,579

6	17562158	0,579
6	17562363	0,579
6	17562636	0,579
6	17562859	0,579
6	17562860	0,579
6	17563084	0,579
6	17563304	0,579
6	17563907	0,579
6	17563911	0,579
6	17563945	0,579
6	17563986	0,579
6	17564583	0,579
6	17565130	0,579
6	17565183	0,579
6	17565565	0,579
6	17593736	0,579
6	17596720	0,579
6	17596721	0,579
6	24711581	0,605
6	24711738	0,605
6	24711837	0,605
6	24711890	0,684
6	24712039	0,684
6	24712166	0,684
6	24712219	0,684
6	24712220	0,684
6	24712317	0,684
6	24712381	0,684
6	24712409	0,684
6	24712580	0,684
6	24712598	0,684
6	24712661	0,684
6	24712743	0,684
6	24712777	0,684
6	24712813	0,684
6	24712854	0,684
6	24712870	0,684
6	24712913	0,684
6	24712963	0,684
6	24713064	0,605
6	24713075	0,605
6	24713100	0,605
6	24713127	0,605
6	24713163	0,605
6	24713551	0,605
6	24713686	0,605
6	24713728	0,605
6	24713898	0,605

6	24713950	0,605
6	24714002	0,605
6	24714013	0,605
6	24714444	0,684
6	24714912	0,605
6	24715166	0,605
6	24715567	0,684
6	24715785	0,684
6	24715843	0,684
6	24715899	0,684
6	24716291	0,605
6	24716795	0,605
6	24717725	0,605
6	24717820	0,684
6	24718269	0,684
6	24718322	0,684
6	24718366	0,684
6	24718562	0,684
6	24718614	0,684
6	24718620	0,684
6	24718863	0,605
6	24719026	0,684
6	24719322	0,684
6	24719423	0,605
6	24719446	0,684
6	24719548	0,605
6	24719618	0,605
6	24719820	0,605
6	24719913	0,605
6	24720179	0,605
6	24720257	0,605
6	24720276	0,605
6	24720389	0,684
6	24720452	0,605
6	24720458	0,684
6	24720896	0,684
6	24720952	0,605
6	24721301	0,684
6	24721420	0,684
6	24721468	0,605
6	24721497	0,684
6	24721811	0,684
6	24721828	0,684
6	24722099	0,605
6	24722303	0,605
6	24722509	0,605
6	24722791	0,605
6	24722959	0,684

6	24723123	0,605
6	24723195	0,605
6	24723231	0,605
6	24723598	0,684
6	24724354	0,605
6	24724666	0,684
6	24724999	0,684
6	24725352	0,605
6	24725389	0,605
6	24725573	0,605
6	24725634	0,684
6	24725672	0,605
6	24725711	0,684
6	24725840	0,684
6	24725987	0,684
6	24726095	0,605
6	24726334	0,605
6	24726440	0,605
6	24726470	0,605
6	24726539	0,605
6	24726617	0,605
6	24726638	0,605
6	24726710	0,605
6	24726771	0,605
6	24726963	0,605
6	24731304	0,605
6	24764269	0,605
6	24765236	0,684
6	24765281	0,684
6	24765531	0,684
6	24765596	0,605
6	24765978	0,684
6	24765995	0,684
6	24766246	0,684
6	24766710	0,684
6	24766750	0,684
6	24766801	0,684
6	24767022	0,605
6	24768331	0,684
6	24768359	0,605
6	24768360	0,605
6	24768609	0,684
6	24768620	0,684
6	24768820	0,684
6	24769501	0,684
6	24769685	0,684
6	24769733	0,605
6	24769765	0,605

6	24769942	0,605
6	24770035	0,605
6	24770077	0,605
6	24770157	0,605
6	24770341	0,605
6	24770745	0,605
6	24771142	0,605
6	24771289	0,605
6	24771291	0,605
6	24771400	0,684
6	24771530	0,605
6	24771596	0,605
6	24771640	0,605
6	24771711	0,684
6	24771754	0,605
6	24771786	0,605
6	24772005	0,605
6	24772225	0,684
6	24772352	0,684
6	24772455	0,605
6	24772588	0,684
6	24772733	0,684
6	24772884	0,684
6	24772885	0,684
6	24772953	0,684
6	24773139	0,684
6	24773186	0,684
6	24773214	0,605
6	24773337	0,684
6	24774170	0,605
6	24774171	0,605
6	24774516	0,684
6	24775477	0,605
6	24775487	0,605
6	24776141	0,684
6	24776631	0,684
6	24776681	0,684
6	24776693	0,684
6	24776751	0,684
6	24776963	0,684
6	24777027	0,605
6	24777113	0,605
6	24777187	0,605
6	24777310	0,605
6	24777324	0,605
6	24777398	0,605
6	24777513	0,605
6	24777554	0,605

6	24777566	0,605
6	24777612	0,605
6	24777632	0,605
6	24777713	0,684
6	24777730	0,605
6	24777731	0,605
6	24777747	0,605
6	24777849	0,605
6	24777874	0,605
6	24777899	0,605
6	24777909	0,605
6	24777911	0,605
6	24777924	0,605
6	24777999	0,605
6	24778005	0,605
6	24778126	0,605
6	24778601	0,605
6	24778709	0,605
6	24778726	0,605
6	24778867	0,605
6	24778928	0,605
6	24778935	0,605
6	24779108	0,684
6	24779143	0,684
6	24779207	0,684
6	24779852	0,684
6	24780020	0,684
6	24780065	0,684
6	24780126	0,684
6	24780159	0,684
6	24780368	0,605
6	24780402	0,605
6	24780801	0,605
6	24780873	0,684
6	24780890	0,684
6	24780912	0,684
6	24781028	0,605
6	24781328	0,684
6	24781392	0,684
6	24781463	0,684
6	24781959	0,684
6	24782036	0,684
6	24782074	0,605
6	24782107	0,684
6	24782206	0,605
6	24782211	0,605
6	24782234	0,605
6	24782448	0,605

6	24782450	0,684
6	24782471	0,605
6	24782562	0,605
6	24782643	0,605
6	24782910	0,684
6	24783029	0,684
6	24783317	0,605
6	24783411	0,605
6	24783619	0,605
6	24783620	0,605
6	24783680	0,605
6	24783960	0,684
6	24784106	0,684
6	24785017	0,684
6	24785883	0,605
6	24786613	0,684
6	24786990	0,684
6	24787287	0,605
6	24787319	0,684
6	24787416	0,605
6	24787672	0,605
6	24787708	0,605
6	24787856	0,684
6	24787947	0,605
6	24787951	0,605
6	24787953	0,605
6	24788028	0,605
6	24788061	0,605
6	24788156	0,605
6	24788196	0,605
6	24788297	0,605
6	24788322	0,605
6	24788452	0,605
6	24788587	0,605
6	24788693	0,605
6	24788714	0,605
6	24788786	0,605
6	24788816	0,605
6	24788881	0,605
6	24788882	0,605
6	24788935	0,605
6	24788942	0,605
6	24789714	0,605
6	24789908	0,605
6	24789965	0,605
6	24789966	0,605
6	24789986	0,605
6	24790128	0,605

6	24790156	0,605
6	24790391	0,605
6	24790474	0,684
6	24790513	0,684
6	24790562	0,605
6	24790653	0,605
6	24790877	0,684
6	24790878	0,684
6	24790968	0,684
6	24791020	0,605
6	24791021	0,605
6	24791157	0,684
6	24792100	0,605
6	24792333	0,605
6	24792340	0,605
6	24792838	0,605
6	24793070	0,684
6	24793091	0,684
6	24793182	0,605
6	24793354	0,684
6	24793625	0,605
6	24793662	0,605
6	24793915	0,605
6	24794335	0,605
6	24794488	0,605
6	24794585	0,684
6	24794958	0,605
6	24794998	0,605
6	24795319	0,605
6	24796469	0,605
6	24796569	0,605
6	24796635	0,684
6	24797140	0,684
6	24797617	0,684
6	24797944	0,684
6	24798086	0,684
6	24798535	0,684
6	24799636	0,684
6	24799830	0,605
6	24800107	0,605
6	24800274	0,684
6	24800405	0,684
6	24800907	0,605
6	24801034	0,684
6	24801146	0,684
6	24801255	0,684
6	24801305	0,684
6	24803355	0,684

6	24804435	0,605
6	24805587	0,605
6	24805672	0,605
6	24806067	0,605
6	24806352	0,605
6	24806463	0,605
6	24806469	0,684
6	24806521	0,605
6	24806736	0,684
6	24807278	0,605
6	24807396	0,605
6	24807645	0,684
6	24807870	0,605
6	24808013	0,684
6	24808704	0,605
6	24808918	0,605
6	24809021	0,684
6	24809073	0,684
6	24809151	0,684
6	24809342	0,684
6	24809354	0,684
6	24809355	0,684
6	24809360	0,684
6	24809364	0,684
6	24809366	0,684
6	24809397	0,684
6	24809580	0,605
6	24809662	0,684
6	24809827	0,605
6	24809936	0,605
6	24810152	0,684
6	24810681	0,605
6	24811182	0,684
6	24811219	0,605
6	24811256	0,684
6	24811305	0,684
6	24811562	0,684
6	24811671	0,605
6	24811725	0,605
6	24811731	0,605
6	24811802	0,605
6	24811878	0,605
6	24812031	0,605
6	24812173	0,605
6	24812278	0,605
6	24812495	0,605
6	24812506	0,605
6	24812597	0,605

6	24812764	0,605
6	24812860	0,684
6	24812906	0,684
6	24812937	0,684
6	24813022	0,684
6	24813244	0,684
6	24813639	0,684
6	24813763	0,605
6	24813869	0,684
6	24813870	0,684
6	24813893	0,684
6	24813936	0,684
6	24813937	0,684
6	24814000	0,684
6	24814199	0,684
6	24814226	0,605
6	24814473	0,684
6	24815135	0,605
6	24815176	0,605
6	24815221	0,605
6	24815243	0,684
6	24815269	0,605
6	24815751	0,684
6	24815784	0,684
6	24815836	0,684
6	24815874	0,684
6	24815946	0,684
6	24815987	0,684
6	24816111	0,684
6	24816136	0,684
6	24816424	0,684
6	24816429	0,684
6	24816637	0,684
6	24817217	0,605
6	24817222	0,605
6	24817320	0,605
6	24817354	0,684
6	24817502	0,605
6	24817623	0,605
6	24817828	0,605
6	24817879	0,605
6	24817929	0,605
6	24817936	0,605
6	24817948	0,605
6	24817949	0,605
6	24817962	0,605
6	24817964	0,605
6	24817966	0,605

6	24817969	0,605
6	24817970	0,605
6	24817977	0,605
6	24817984	0,605
6	24818021	0,605
6	24818024	0,605
6	24818055	0,605
6	24818190	0,605
6	24818295	0,605
6	24818297	0,605
6	24818307	0,605
6	24818528	0,605
6	24818615	0,605
6	24818696	0,605
6	24818930	0,684
6	24819038	0,605
6	24819093	0,605
6	24819142	0,605
6	24819345	0,605
6	24819409	0,605
6	24819411	0,605
6	24819815	0,605
6	24820191	0,684
6	24820348	0,605
6	24820363	0,605
6	24820374	0,605
6	24820399	0,605
6	24820438	0,605
6	24820687	0,605
6	24821172	0,684
6	24821318	0,684
6	24821319	0,684
6	24821324	0,605
6	24821336	0,684
6	24821381	0,684
6	24821864	0,605
6	24821865	0,605
6	24822184	0,684
6	24822821	0,605
6	24823301	0,684
6	24823324	0,684
6	24823395	0,684
6	24823414	0,684
6	24823452	0,684
6	24823460	0,684
6	24823583	0,684
6	24823612	0,684
6	24823711	0,684

6	24823750	0,684
6	24823754	0,684
6	24823841	0,684
6	24823902	0,684
6	24823913	0,684
6	24823992	0,684
6	24824143	0,684
6	24824536	0,684
6	24824590	0,684
6	24824591	0,684
6	24824622	0,684
6	24824699	0,684
6	24825189	0,605
6	24825190	0,605
6	24825241	0,605
6	24825734	0,684
6	24825802	0,684
6	24826167	0,605
6	24826377	0,605
6	24826690	0,605
6	24826761	0,605
6	24827230	0,605
6	24865851	0,605
6	24866687	0,684
6	24866898	0,684
6	24866918	0,684
6	24867143	0,684
6	24867305	0,684
6	24867894	0,684
6	24867953	0,684
6	24867955	0,684
6	24867961	0,684
6	24867981	0,684
6	24868014	0,684
6	24868137	0,605
6	24868370	0,605
6	24868385	0,605
6	24869564	0,684
6	24869818	0,605
6	24869937	0,684
6	24869947	0,684
6	24869989	0,684
6	24870070	0,684
6	24871391	0,605
6	24871403	0,605
6	24871538	0,605
6	24871587	0,605
6	24871674	0,605

6	24871808	0,605
6	24871858	0,605
6	24872562	0,605
6	24872716	0,684
6	24872847	0,684
6	24872961	0,605
6	24873030	0,684
6	24873035	0,684
6	24873118	0,684
6	24873345	0,684
6	24873626	0,605
6	24874371	0,605
6	24874394	0,605
6	24874894	0,684
6	24874945	0,605
6	24874953	0,605
6	24875133	0,605
6	24875457	0,605
6	24875806	0,684
6	24876057	0,684
6	24876930	0,605
6	24877182	0,605
6	24877351	0,605
6	24877376	0,605
6	24877377	0,605
6	24877593	0,605
6	24877601	0,605
6	24877614	0,605
6	24877628	0,605
6	24877833	0,684
6	24877923	0,605
6	24878006	0,684
6	24878107	0,605
6	24878287	0,605
6	24878349	0,684
6	24878376	0,684
6	24878377	0,684
6	24878784	0,684
6	24879009	0,605
6	24886337	0,605
6	24886426	0,605
6	24886632	0,605
6	24886749	0,605
6	24886899	0,684
6	24887059	0,605
6	24887121	0,605
6	24887543	0,684
6	24887727	0,684

6	24887772	0,684
6	24888000	0,684
6	24888180	0,684
6	24888411	0,684
6	24888768	0,605
6	24888955	0,605
6	24889258	0,605
6	24889451	0,684
6	24889651	0,684
6	24889736	0,684
6	24889742	0,605
6	24889787	0,684
6	24890540	0,684
6	24890677	0,684
6	24890682	0,684
6	24890754	0,684
6	24890757	0,684
6	24890766	0,684
6	24890773	0,684
6	24890837	0,684
6	24890848	0,684
6	24891018	0,684
6	24891303	0,605
6	24891425	0,684
6	24891592	0,605
6	24891631	0,605
6	24891635	0,605
6	24891642	0,605
6	24891818	0,605
6	24891921	0,605
6	24892023	0,605
6	24892455	0,605
6	24892636	0,605
6	24892840	0,684
6	24892961	0,684
6	24893084	0,605
6	24893400	0,605
6	24893567	0,605
6	24893643	0,605
6	24893745	0,605
6	24893915	0,684
6	24894070	0,684
6	24894114	0,605
6	24894369	0,684
6	24894390	0,684
6	24894403	0,684
6	24894404	0,684
6	24894406	0,684

6	24894408	0,684
6	24894666	0,684
6	24894683	0,684
6	24895682	0,684
6	24895716	0,684
6	24895803	0,684
6	24895806	0,684
6	24895972	0,684
6	24896031	0,684
6	24896053	0,684
6	24896054	0,684
6	24896129	0,684
6	24896360	0,684
6	24910731	0,684
6	24910733	0,684
6	24910774	0,684
6	24910790	0,684
6	24910879	0,684
6	24911462	0,684
6	24911650	0,684
6	24911660	0,684
6	24911725	0,684
6	24911739	0,684
6	24911755	0,684
6	24911926	0,684
6	24912458	0,684
6	24912490	0,684
6	24912507	0,684
6	24912656	0,684
6	24912666	0,684
6	24912855	0,684
6	24912862	0,684
6	24913014	0,684
6	24913039	0,684
6	24913303	0,684
6	24949153	0,684
6	24953279	0,684
6	24954050	0,684
6	24954110	0,684
6	25077997	0,684
6	25078089	0,684
6	25078286	0,684
6	25081105	0,684
6	25081884	0,684
6	25081935	0,684
6	25082010	0,684
6	25082046	0,684
6	25089050	0,684

6	25089856	0,684
6	25089868	0,684
6	25089922	0,684
6	25090048	0,684
6	25090184	0,684
6	25090325	0,684
6	25090448	0,684
6	25090451	0,684
6	25090452	0,684
6	25090620	0,684
6	25090683	0,684
6	25096043	0,684
6	25217711	0,684
6	25217723	0,684
6	25217818	0,684
6	25217927	0,684
6	25241780	0,684
6	25241794	0,684
6	25242732	0,684
6	25242812	0,684
6	25243594	0,684
6	25243959	0,684
6	25244233	0,684
6	25244522	0,684
6	25244723	0,684
6	25245199	0,684
6	25246463	0,684
6	25246475	0,684
6	25246509	0,684
6	25246774	0,684
6	25246953	0,684
6	25247164	0,684
6	25247165	0,684
6	25254353	0,684
6	25255117	0,684
6	25255259	0,684
6	25257957	0,684
6	25307621	0,684
6	25307672	0,684
6	25307764	0,684
6	25307962	0,684
6	25307969	0,684
6	25308117	0,684
6	25308180	0,684
6	25308352	0,684
6	25308471	0,684
6	25308617	0,684
6	25308834	0,684

6	25309046	0,684
6	25309189	0,684
6	25309264	0,684
6	25309396	0,684
6	25309654	0,684
6	25311782	0,684
6	25312026	0,684
6	25312992	0,684
6	25314420	0,684
6	25314458	0,684
6	25314459	0,684
6	25314472	0,684
6	25316054	0,684
6	25316086	0,684
6	25316276	0,684
6	25317023	0,684
6	25317204	0,684
6	25317733	0,684
6	25317808	0,684
6	25318564	0,684
6	25319022	0,684
6	25319031	0,684
6	25319039	0,763
6	25319088	0,711
6	25319135	0,737
6	25319284	0,737
6	25319362	0,711
6	25319412	0,711
6	25319565	0,711
6	25319767	0,737
6	25319874	0,500
6	25320126	0,684
6	25320515	0,684
6	25320860	0,711
6	25320884	0,500
6	25321328	0,711
6	25321760	0,526
6	25321772	0,711
6	25321852	0,684
6	25321873	0,711
6	25321891	0,711
6	25321994	0,684
6	25322034	0,763
6	25322082	0,763
6	25322189	0,789
6	25322191	0,763
6	25322210	0,789
6	25322212	0,763

6	25322241	0,684
6	25322318	0,711
6	25322363	0,684
6	25322460	0,526
6	25322820	0,763
6	25322930	0,789
6	25322971	0,500
6	25323271	0,711
6	25323284	0,711
6	25323290	0,789
6	25323304	0,711
6	25323313	0,684
6	25323327	0,737
6	25323403	0,684
6	25323407	0,500
6	25323457	0,684
6	25323503	0,684
6	25323576	0,684
6	25323584	0,500
6	25323713	0,684
6	25323865	0,684
6	25324178	0,684
6	25324358	0,684
6	25324499	0,500
6	25325165	0,684
6	25325465	0,684
6	25325699	0,526
6	25325843	0,526
6	25326073	0,684
6	25326258	0,684
6	25326289	0,684
6	25326713	0,684
6	25327257	0,500
6	25341408	0,684
6	25341444	0,684
6	25341497	0,684
6	25341671	0,684
6	25341682	0,684
6	25341861	0,684
6	25342363	0,684
6	25342572	0,684
6	25342638	0,526
6	25342717	0,500
6	25342941	0,684
6	25343006	0,684
6	25343051	0,684
6	25343071	0,684
6	25343181	0,684

6	25343687	0,684
6	25343950	0,684
6	25344113	0,684
6	25344157	0,526
6	25345281	0,737
6	25348780	0,684
6	25357329	0,684
6	25357356	0,684
6	25357423	0,684
6	25357434	0,684
6	25357814	0,684
6	25368545	0,684
6	25370937	0,684
6	25372655	0,684
6	25373261	0,684
6	25374391	0,684
6	25374411	0,684
6	25375832	0,684
6	25376515	0,684
6	25376955	0,684
6	25377386	0,684
6	25377503	0,684
6	25385919	0,684
6	25386015	0,684
6	25386169	0,684
6	25386291	0,684
6	25387529	0,684
6	25387611	0,684
6	25387615	0,684
6	25387619	0,684
6	25387631	0,684
6	25388073	0,684
6	25388089	0,684
6	25388329	0,684
6	25395143	0,684
6	25395429	0,684
6	25396926	0,684
6	25398373	0,684
6	25400953	0,684
6	25400958	0,684
6	25400965	0,684
6	25401484	0,684
6	25402552	0,684
6	25402651	0,684
6	25402747	0,684
6	25405238	0,684
6	25405324	0,684
6	25407229	0,684

6	25407242	0,684
6	25407435	0,684
6	25407727	0,684
6	25408199	0,684
6	25408969	0,684
6	25408991	0,684
6	25409032	0,684
6	25410671	0,684
6	25410784	0,684
6	25427464	0,684
6	25427508	0,684
6	25427510	0,684
6	25427525	0,684
6	25427536	0,684
6	25428009	0,684
6	25428117	0,684
6	25430472	0,684
6	25439205	0,684
6	16945167	0,508
6	16945417	0,508
6	17521770	0,584
6	17521917	0,584
6	17522191	0,584
6	24952918	0,500
6	25078330	0,500
6	25078643	0,500
6	25078679	0,500
6	25078854	0,500
6	25078894	0,500
6	25079110	0,500
6	25079161	0,500
6	25079203	0,500
6	25079282	0,500
6	25079402	0,500
6	25079495	0,500
6	25079541	0,500
6	25080802	0,500
6	25080855	0,500
6	25080922	0,500
6	25080951	0,500
6	25080994	0,500
6	25081080	0,500
6	25081087	0,500
6	25081096	0,500
6	25081102	0,500
6	25081103	0,500
6	25081117	0,500
6	25081134	0,500

6	25081231	0,500
6	25081242	0,500
6	25081269	0,500
6	25081275	0,500
6	25081592	0,500
6	25081888	0,500
6	25088112	0,500
6	25088202	0,500
6	25088688	0,500
6	25088918	0,500
6	25089169	0,500
6	25090241	0,500
6	25090355	0,500
6	25090475	0,500
6	25090555	0,500
6	25090693	0,500
6	25091386	0,500
6	25092818	0,500
6	25092849	0,500
6	25093278	0,500
6	25094614	0,500
6	25094711	0,500
6	25094745	0,500
6	25243749	0,500
6	25245025	0,500
6	25246140	0,500
6	25246938	0,500
6	25251749	0,500
6	25252421	0,500
6	25252424	0,500
6	25253557	0,500
6	25254085	0,500
6	25254385	0,500
6	25255135	0,500
6	25255155	0,500
6	25255226	0,500
6	25255538	0,500
6	25258545	0,500
6	25258806	0,500
6	25312708	0,500
6	25314962	0,500
6	25314991	0,500
6	25315413	0,500
6	25315626	0,500
6	25316018	0,500
6	25316635	0,500
6	25316698	0,500
6	25316995	0,500

6	25317076	0,500
6	25317163	0,500
6	25317335	0,500
6	25319455	0,500
6	25319756	0,500
6	25320270	0,500
6	25320310	0,500
6	25320619	0,500
6	25320655	0,500
6	25320844	0,500
6	25320945	0,500
6	25321611	0,500
6	25321849	0,500
6	25322164	0,500
6	25322213	0,500
6	25322813	0,500
6	25323269	0,500
6	25323650	0,500
6	25323911	0,500
6	25324116	0,500
6	25324677	0,500
6	25325847	0,500
6	25326511	0,500
6	25327466	0,500
6	25327543	0,500
6	25327567	0,500
6	25327631	0,500
6	25327708	0,500
6	25327976	0,500
6	25330052	0,500
6	25330206	0,500
6	25330218	0,500
6	25330269	0,500
6	25330389	0,500
6	25330448	0,500
6	25330471	0,500
6	25330503	0,500
6	25330543	0,500
6	25330654	0,500
6	25330664	0,500
6	25330679	0,500
6	25339738	0,500
6	25339769	0,500
6	25340172	0,500
6	25340765	0,500
6	25340805	0,500
6	25341447	0,500
6	25344936	0,500

6	25345112	0,500
6	25345178	0,500
6	25345311	0,500
6	25345312	0,500
6	25345328	0,500
6	25345384	0,500
6	25345388	0,500
6	25345392	0,500
6	25345453	0,500
6	25345612	0,500
6	25345643	0,500
6	25345833	0,500
6	25345885	0,500
6	25345968	0,500
6	25346023	0,500
6	25346131	0,500
6	25346727	0,500
6	25348178	0,500
6	25348482	0,500
6	25348502	0,500
6	25348698	0,500
6	25357717	0,500
6	25357782	0,500
6	25357784	0,500
6	25368596	0,500
6	25370519	0,500
6	25370773	0,500
6	25372078	0,500
6	25372719	0,500
6	25372912	0,500
6	25374581	0,500
6	25374760	0,500
6	25377028	0,500
6	25377976	0,500
6	25378133	0,500
6	25378180	0,500
6	25378395	0,500
6	25387396	0,500
6	25395336	0,500
6	25395685	0,500
6	25396132	0,500
6	25396402	0,500
6	25396897	0,500
6	25397019	0,500
6	25397086	0,500
6	25397225	0,500
6	25397589	0,500
6	25397653	0,500

6	25397780	0,500
6	25397798	0,500
6	25397814	0,500
6	25397815	0,500
6	25398251	0,500
6	25398316	0,500
6	25398396	0,500
6	25398576	0,500
6	25400996	0,500
6	25400997	0,500
6	25401065	0,500
6	25401353	0,500
6	25401470	0,500
6	25401665	0,500
6	25402349	0,500
6	25402391	0,500
6	25405090	0,500
6	25405302	0,500
6	25405325	0,500
6	25405389	0,500
6	25405423	0,500
6	25405517	0,500
6	25406033	0,500
6	25406079	0,500
6	25407014	0,500
6	25407033	0,500
6	25407351	0,500
6	25407741	0,500
6	25408386	0,500
6	25408802	0,500
6	25410245	0,500
6	25410383	0,500
6	25415470	0,500
6	25415647	0,500
6	25427907	0,500
6	25427961	0,500
6	25430569	0,500
6	25438116	0,500
6	16934444	0,553
6	16934494	0,579
6	16934749	0,632
6	16936216	0,553
6	16936263	0,872
6	16936590	0,846
6	16937668	0,872
6	16940710	0,872
6	16941003	0,872
6	16941579	0,846

6	16944642	0,504
6	16944815	0,530
6	16946528	0,530
6	16950404	0,500
6	16951088	0,500
6	16951467	0,846
6	16952058	0,872
6	16953428	0,820
6	16953617	0,526
6	16953996	0,872
6	16954457	0,767
6	16954624	0,793
6	16954633	0,846
6	16954978	0,846
6	16956274	0,846
6	16956869	0,872
6	16957246	0,872
6	16957747	0,846
6	16958850	0,872
6	16959017	0,846
6	16959818	0,846
6	16959927	0,872
6	16960165	0,846
6	16960198	0,557
6	16961454	0,504
6	16961599	0,793
6	16961643	0,872
6	17023529	0,716
6	17023570	0,716
6	17023614	0,716
6	17023629	0,716
6	17023666	0,716
6	17023671	0,716
6	17023676	0,716
6	17023682	0,716
6	17024213	0,716
6	17026502	0,716
6	17027269	0,716
6	17027274	0,716
6	17027319	0,716
6	17027384	0,716
6	17027570	0,716
6	17029305	0,716
6	17029330	0,557
6	17029399	0,716
6	17029432	0,716
6	17032597	0,716
6	17032833	0,716

6	17032936	0,716
6	17033803	0,716
6	17033869	0,716
6	17033870	0,716
6	17034007	0,716
6	17034020	0,716
6	17034032	0,716
6	17034034	0,716
6	17034045	0,716
6	17034072	0,716
6	17034073	0,716
6	17034143	0,716
6	17034364	0,716
6	17034865	0,716
6	17035107	0,716
6	17035518	0,716
6	17035801	0,557
6	17035817	0,716
6	17036211	0,716
6	17036414	0,557
6	17036433	0,557
6	17036529	0,557
6	17036604	0,557
6	17036643	0,716
6	17036645	0,716
6	17037384	0,716
6	17037552	0,557
6	17037579	0,557
6	17037812	0,716
6	17038334	0,716
6	17039112	0,716
6	17039645	0,716
6	17039739	0,716
6	17039788	0,716
6	17041222	0,716
6	17041317	0,557
6	17041824	0,716
6	17042440	0,716
6	17042812	0,716
6	17043311	0,716
6	17043532	0,716
6	17046540	0,716
6	17046672	0,716
6	17047048	0,716
6	17047569	0,716
6	17047715	0,716
6	17047858	0,557
6	17048330	0,716

6	17048451	0,557
6	17048868	0,557
6	17048882	0,557
6	17048906	0,716
6	17048951	0,716
6	17048961	0,557
6	17049044	0,557
6	17049529	0,557
6	17049530	0,716
6	17049921	0,716
6	17050434	0,557
6	17050522	0,557
6	17050690	0,557
6	17050809	0,557
6	17050812	0,557
6	17050818	0,557
6	17050952	0,557
6	17051129	0,557
6	17051500	0,557
6	17052031	0,557
6	17052186	0,716
6	17052297	0,557
6	17052448	0,716
6	17052628	0,557
6	17052641	0,557
6	17052697	0,557
6	17053270	0,716
6	17053906	0,557
6	17054285	0,557
6	17054543	0,557
6	17054566	0,716
6	17054569	0,716
6	17054618	0,716
6	17054638	0,716
6	17054761	0,716
6	17054778	0,557
6	17054849	0,716
6	17055226	0,557
6	17055262	0,716
6	17055694	0,716
6	17055754	0,716
6	17055755	0,716
6	17056016	0,716
6	17058313	0,716
6	17058457	0,716
6	17058600	0,716
6	17058671	0,716
6	17058672	0,716

6	17058688	0,716
6	17058780	0,716
6	17060029	0,716
6	17060703	0,716
6	17060717	0,716
6	17060896	0,716
6	17061220	0,716
6	17061283	0,716
6	17061340	0,716
6	17061395	0,716
6	17061404	0,716
6	17061412	0,716
6	17061483	0,716
6	17061587	0,716
6	17061770	0,716
6	17062823	0,716
6	17062845	0,716
6	17062875	0,716
6	17062989	0,716
6	17063002	0,716
6	17063059	0,716
6	17063295	0,716
6	17063359	0,716
6	17063719	0,716
6	17063900	0,716
6	17063992	0,716
6	17064065	0,716
6	17064248	0,716
6	17064257	0,716
6	17064268	0,716
6	17065483	0,716
6	17065678	0,716
6	17065723	0,716
6	17066497	0,716
6	17066620	0,716
6	17066663	0,716
6	17066904	0,716
6	17067709	0,557
6	17068664	0,716
6	17068719	0,557
6	17068817	0,557
6	17070627	0,557
6	17070694	0,716
6	17070762	0,716
6	17070769	0,716
6	17070780	0,557
6	17070839	0,716
6	17070884	0,716

6	17071503	0,716
6	17071602	0,557
6	17071633	0,716
6	17071663	0,557
6	17072036	0,716
6	17072369	0,557
6	17073131	0,557
6	17073216	0,557
6	17073318	0,716
6	17073337	0,716
6	17073340	0,716
6	17073427	0,716
6	17073642	0,557
6	17073931	0,716
6	17074425	0,557
6	17074653	0,557
6	17074751	0,557
6	17074794	0,716
6	17074918	0,716
6	17074939	0,716
6	17075192	0,716
6	17075407	0,716
6	17075535	0,716
6	17075562	0,557
6	17075952	0,716
6	17076413	0,716
6	17077527	0,716
6	17077655	0,716
6	17077732	0,557
6	17077998	0,716
6	17078250	0,716
6	17079552	0,716
6	17079998	0,716
6	17080019	0,716
6	17080024	0,716
6	17080027	0,716
6	17081003	0,716
6	17082827	0,716
6	17082832	0,716
6	17082880	0,716
6	17082907	0,716
6	17083437	0,716
6	17083440	0,716
6	17083647	0,716
6	17083880	0,716
6	17084076	0,716
6	17084090	0,716
6	17084876	0,716

6	17085020	0,716
6	17085612	0,716
6	17085627	0,716
6	17091935	0,716
6	17123405	0,716
6	17123560	0,557
6	17140970	0,716
6	17141067	0,716
6	17141259	0,716
6	17141303	0,716
6	17141531	0,716
6	17141809	0,716
6	17143651	0,716
6	17145150	0,716
6	17146235	0,716
6	17149176	0,716
6	17149337	0,716
6	17150065	0,716
6	17150144	0,716
6	17150290	0,716
6	17150303	0,716
6	17150347	0,716
6	17150361	0,716
6	17150539	0,716
6	17150769	0,716
6	17150995	0,716
6	17151018	0,716
6	17151467	0,716
6	17151763	0,716
6	17151765	0,716
6	17163314	0,716
6	17163866	0,716
6	17164024	0,716
6	17164798	0,716
6	17164895	0,716
6	17165611	0,716
6	17166150	0,716
6	17166237	0,716
6	17166344	0,716
6	17166450	0,716
6	17166585	0,716
6	17166677	0,716
6	17167131	0,716
6	17167462	0,716
6	17167533	0,716
6	17167621	0,716
6	17168487	0,716
6	17169024	0,716

6	17169029	0,716
6	17169219	0,716
6	17169312	0,716
6	17169386	0,716
6	17169611	0,716
6	17169699	0,716
6	17170082	0,716
6	17170157	0,716
6	17170205	0,716
6	17170333	0,716
6	17170638	0,716
6	17246955	0,689
6	17246965	0,742
6	17249027	0,742
6	17251256	0,716
6	17253440	0,689
6	17253629	0,633
6	17254771	0,742
6	17257720	0,716
6	17257965	0,584
6	17258691	0,637
6	17259492	0,558
6	17259513	0,663
6	17264516	0,716
6	17267853	0,742
6	17268166	0,611
6	17268180	0,558
6	17268226	0,742
6	17268449	0,663
6	17269580	0,689
6	17269924	0,663
6	17270445	0,742
6	17272656	0,530
6	17272663	0,557
6	17272951	0,583
6	17273454	0,557
6	17274692	0,742
6	17475792	0,609
6	17476337	0,609
6	17476390	0,609
6	17476441	0,609
6	17477750	0,609
6	17477893	0,609
6	17477897	0,609
6	17477907	0,609
6	17477924	0,609
6	17478014	0,609
6	17478686	0,609

6	17478809	0,609
6	17478810	0,609
6	17478837	0,609
6	17478838	0,609
6	17481494	0,609
6	17481501	0,609
6	17481545	0,609
6	17481600	0,609
6	17483842	0,609
6	17485185	0,609
6	17485639	0,609
6	17486168	0,609
6	17486260	0,609
6	17494277	0,609
6	17501502	0,609
6	17506202	0,609
6	17511622	0,609
6	17512053	0,742
6	17512306	0,609
6	17512636	0,609
6	17512696	0,609
6	17513106	0,609
6	17513683	0,742
6	17514321	0,742
6	17514364	0,742
6	17515012	0,609
6	17515223	0,742
6	17515234	0,609
6	17515581	0,742
6	17515584	0,742
6	17516329	0,742
6	17516341	0,742
6	17516400	0,742
6	17516446	0,609
6	17516531	0,609
6	17516658	0,609
6	17516748	0,609
6	17516760	0,609
6	17517484	0,609
6	17517716	0,609
6	17517800	0,609
6	17517914	0,609
6	17518100	0,609
6	17518248	0,609
6	17518289	0,609
6	17518298	0,609
6	17518353	0,609
6	17518355	0,609

6	17518447	0,609
6	17518486	0,609
6	17518591	0,609
6	17518606	0,609
6	17518804	0,742
6	17519248	0,609
6	17519535	0,742
6	17519597	0,609
6	17519752	0,609
6	17519853	0,742
6	17519866	0,609
6	17520022	0,609
6	17520204	0,609
6	17520314	0,609
6	17520656	0,609
6	17521146	0,609
6	17521953	0,742
6	17522051	0,742
6	17522434	0,742
6	17522549	0,742
6	17523751	0,742
6	17523870	0,742
6	17523893	0,742
6	17524019	0,742
6	17524049	0,742
6	17524070	0,742
6	17524093	0,742
6	17524124	0,742
6	17524273	0,742
6	17525270	0,742
6	17527365	0,742
6	17528259	0,742
6	17528430	0,742
6	17528448	0,742
6	17528457	0,742
6	17528475	0,742
6	17528888	0,742
6	17528913	0,742
6	17528940	0,742
6	17529882	0,742
6	17532645	0,742
6	17533058	0,742
6	17533090	0,742
6	17533766	0,742
6	17534043	0,742
6	17534983	0,742
6	17535341	0,742
6	17537560	0,742

6	17537660	0,742
6	17537923	0,742
6	17538314	0,742
6	17538336	0,742
6	17538398	0,742
6	17538474	0,742
6	17538654	0,742
6	17538667	0,742
6	17538707	0,742
6	17538716	0,742
6	17538728	0,742
6	17538817	0,742
6	17538828	0,742
6	17538829	0,742
6	17538847	0,742
6	17538875	0,742
6	17538876	0,742
6	17539170	0,742
6	17539347	0,742
6	17539766	0,742
6	17539813	0,742
6	17540118	0,742
6	17540297	0,742
6	17545048	0,742
6	17545104	0,742
6	17546212	0,742
6	17546332	0,742
6	17546389	0,742
6	17547697	0,742
6	17548092	0,742
6	17550567	0,742
6	17551509	0,742
6	17551675	0,742
6	17551892	0,742
6	17551933	0,742
6	17552220	0,742
6	17552221	0,742
6	17552259	0,742
6	17552260	0,742
6	17552585	0,742
6	17552662	0,742
6	17552680	0,742
6	17552722	0,742
6	17553936	0,742
6	17554213	0,742
6	17554819	0,742
6	17555095	0,742
6	17555145	0,742

6	17555226	0,742
6	17555347	0,742
6	17555601	0,742
6	17555619	0,742
6	17559121	0,742
6	17559514	0,742
6	17560173	0,742
6	17560177	0,742
6	17560423	0,742
6	17560424	0,742
6	17560470	0,742
6	17560889	0,742
6	17561072	0,742
6	17561198	0,742
6	17561475	0,742
6	17561499	0,742
6	17561545	0,742
6	17561596	0,742
6	17562346	0,742
6	17562440	0,742
6	17562459	0,742
6	17562461	0,742
6	17562588	0,742
6	17562638	0,742
6	17562814	0,742
6	17562966	0,742
6	17563184	0,742
6	17563386	0,742
6	17563523	0,742
6	17563749	0,742
6	17563862	0,742
6	17563974	0,742
6	17564655	0,742
6	17565308	0,742
6	17565890	0,742
6	17568326	0,742
6	17569200	0,742
6	17569228	0,742
6	17569249	0,742
6	17569329	0,742
6	17569376	0,742
6	17569379	0,742
6	17569383	0,742
6	17569389	0,742
6	17569450	0,742
6	17569456	0,742
6	17569463	0,742
6	17569543	0,742

6	17570208	0,742
6	17570866	0,742
6	17570962	0,742
6	17571410	0,742
6	17571664	0,742
6	17571694	0,742
6	17571741	0,742
6	17571921	0,742
6	17572290	0,742
6	17573412	0,742
6	17574492	0,742
6	17574593	0,742
6	17574637	0,742
6	17574882	0,742
6	17574884	0,742
6	17575013	0,742
6	17575138	0,742
6	17575156	0,742
6	17575348	0,742
6	17575353	0,742
6	17575432	0,742
6	17575438	0,742
6	17575444	0,742
6	17575481	0,742
6	17575502	0,742
6	17575603	0,742
6	17575609	0,742
6	17575736	0,742
6	17575803	0,742
6	17575829	0,742
6	17575831	0,742
6	17575835	0,742
6	17576009	0,742
6	17576062	0,742
6	17576068	0,742
6	17576082	0,742
6	17576094	0,742
6	17576098	0,742
6	17576106	0,742
6	17576107	0,742
6	17576127	0,742
6	17576138	0,742
6	17576143	0,742
6	17576144	0,742
6	17576308	0,742
6	17576314	0,742
6	17576322	0,742
6	17576403	0,742

6	17576410	0,742
6	17576418	0,742
6	17576428	0,742
6	17576486	0,742
6	17576505	0,742
6	17576531	0,742
6	17576656	0,742
6	17576662	0,742
6	17577586	0,742
6	17577659	0,742
6	17577673	0,742
6	17577872	0,742
6	17578098	0,742
6	17578111	0,742
6	17578115	0,742
6	17578116	0,742
6	17578359	0,742
6	17578505	0,742
6	17578846	0,742
6	17578884	0,742
6	17578932	0,742
6	17579852	0,742
6	17579937	0,742
6	17579958	0,742
6	17579995	0,742
6	17580047	0,742
6	17580051	0,742
6	17580189	0,742
6	17580248	0,742
6	17580283	0,742
6	17580292	0,742
6	17580466	0,742
6	17580521	0,742
6	17580586	0,742
6	17580592	0,742
6	17580797	0,742
6	17580915	0,742
6	17580942	0,742
6	17581225	0,742
6	17581296	0,742
6	17581299	0,742
6	17581341	0,742
6	17581357	0,742
6	17581379	0,742
6	17581427	0,742
6	17581593	0,742
6	17581632	0,742
6	17581634	0,742

6	17581649	0,742
6	17581673	0,742
6	17581701	0,742
6	17581741	0,742
6	17581799	0,742
6	17581809	0,742
6	17581897	0,742
6	17581912	0,742
6	17581915	0,742
6	17581935	0,742
6	17581938	0,742
6	17582006	0,742
6	17582295	0,742
6	17582313	0,742
6	17582322	0,742
6	17582365	0,742
6	17582604	0,742
6	17582674	0,742
6	17582745	0,742
6	17582761	0,742
6	17582871	0,742
6	17582943	0,742
6	17583044	0,742
6	17583122	0,742
6	17583175	0,742
6	17583287	0,742
6	17583430	0,742
6	17583488	0,742
6	17583579	0,742
6	17583589	0,742
6	17583594	0,742
6	17583674	0,742
6	17583731	0,742
6	17583776	0,742
6	17583897	0,742
6	17583925	0,742
6	17584002	0,742
6	17584143	0,742
6	17584429	0,742
6	17584609	0,742
6	17584658	0,742
6	17585171	0,742
6	17586097	0,742
6	17586207	0,742
6	17586223	0,742
6	17586451	0,742
6	17586612	0,742
6	17587699	0,742

6	17587840	0,742
6	17587880	0,742
6	17588101	0,742
6	17588105	0,742
6	17588106	0,742
6	17588196	0,742
6	17588246	0,742
6	17588329	0,742
6	17588429	0,742
6	17588594	0,742
6	17588725	0,742
6	17588739	0,742
6	17588887	0,742
6	17589169	0,742
6	17589250	0,742
6	17589317	0,742
6	17589389	0,742
6	17589404	0,742
6	17589490	0,742
6	17589491	0,742
6	17589899	0,742
6	17590062	0,742
6	17590183	0,742
6	17590664	0,742
6	17591741	0,742
6	17591996	0,742
6	17592054	0,742
6	17592080	0,742
6	17592285	0,742
6	17592783	0,742
6	17592817	0,742
6	17592828	0,742
6	17592909	0,742
6	17593063	0,742
6	17593121	0,742
6	17593237	0,742
6	17593245	0,742
6	17593249	0,742
6	17594322	0,742
6	17594621	0,742
6	17594804	0,742
6	17594987	0,742
6	17595333	0,742
6	17595398	0,742
6	17595840	0,742
6	17596058	0,742
6	17596060	0,742
6	17596258	0,742

6	17596297	0,742
6	17596342	0,742
6	17596422	0,742
6	17596575	0,742
6	17596602	0,742
6	17596608	0,742
6	17596622	0,742
6	17596632	0,742
6	17596633	0,742
6	17596678	0,742
6	17596766	0,742
6	17596825	0,742
6	17596828	0,742
6	17596840	0,742
6	17597176	0,742
6	17597191	0,742
6	17597231	0,742
6	17597245	0,742
6	17597497	0,742
6	17597711	0,742
6	17597915	0,742
6	17598090	0,742
6	17598878	0,742
6	17598897	0,742
6	17598903	0,742
6	17599916	0,742
6	17599932	0,742
6	17600045	0,742
6	17600056	0,742
6	17600109	0,742
6	17600115	0,742
6	17600385	0,742
6	17600415	0,742
6	17600444	0,742
6	17600450	0,742
6	17600502	0,742
6	17600546	0,742
6	17600560	0,742
6	17600593	0,742
6	17600705	0,742
6	17600778	0,742
6	17600905	0,742
6	17600912	0,742
6	17602220	0,742
6	17602299	0,742
6	17602329	0,742
6	17602368	0,742
6	17602477	0,742

6	17602485	0,742
6	17602486	0,742
6	17602495	0,742
6	17602580	0,742
6	17602622	0,742
6	17602636	0,742
6	17602747	0,742
6	17602899	0,742
6	17603157	0,742
6	17604023	0,742
6	17604283	0,742
6	17604533	0,742
6	17604658	0,742
6	17604946	0,742
6	17605149	0,742
6	17605679	0,742
6	17605779	0,742
6	17605827	0,742
6	17605859	0,742
6	17605906	0,742
6	17605907	0,742
6	17605953	0,742
6	17605956	0,742
6	17605974	0,742
6	17606039	0,742
6	17606049	0,742
6	17606061	0,742
6	17606062	0,742
6	17606479	0,742
6	17606558	0,742
6	17606769	0,742
6	17606906	0,742
6	17607259	0,742
6	17607297	0,742
6	17607687	0,742
6	17607798	0,742
6	17607838	0,742
6	17607852	0,742
6	17607942	0,742
6	17607944	0,742
6	17607946	0,742
6	17607954	0,742
6	17607959	0,742
6	17607967	0,742
6	17607983	0,742
6	17608026	0,742
6	17608054	0,742
6	17608081	0,742

6	17608159	0,742
6	17608168	0,742
6	17608174	0,742
6	17608189	0,742
6	17608200	0,742
6	17608306	0,742
6	17608311	0,742
6	17608315	0,742
6	17608316	0,742
6	17608330	0,742
6	17608362	0,742
6	17608363	0,742
6	17608366	0,742
6	17608375	0,742
6	17608461	0,742
6	17608889	0,742
6	17609067	0,742
6	17609084	0,742
6	17609130	0,742
6	17609149	0,742
6	17609153	0,742
6	17609255	0,742
6	17609336	0,742
6	17609369	0,742
6	17609603	0,742
6	17609617	0,742
6	17609661	0,742
6	17609664	0,742
6	17609743	0,742
6	17609755	0,742
6	17609758	0,742
6	17609770	0,742
6	17610079	0,742
6	17610307	0,742
6	17610332	0,742
6	17610341	0,742
6	17610423	0,742
6	17610448	0,742
6	17610450	0,742
6	17610500	0,742
6	17610518	0,742
6	17610663	0,742
6	17610757	0,742
6	17610906	0,742
6	17610926	0,742
6	17611077	0,742
6	17611133	0,742
6	17611155	0,742

6	17611189	0,742
6	17611499	0,742
6	17611524	0,742
6	17611662	0,742
6	17611735	0,742
6	17611741	0,742
6	17611794	0,742
6	17611881	0,742
6	17611957	0,742
6	17612302	0,742
6	17612326	0,742
6	17612341	0,742
6	17612348	0,742
6	17612429	0,742
6	17612432	0,742
6	17612433	0,742
6	17612444	0,742
6	17612468	0,742
6	17613594	0,742
6	17613690	0,742
6	17613939	0,742
6	17613945	0,742
6	17614137	0,742
6	17614198	0,742
6	17614229	0,742
6	17614270	0,742
6	17614585	0,742
6	17614658	0,742
6	17614752	0,742
6	17614753	0,742
6	17614786	0,742
6	17614862	0,742
6	17615189	0,742
6	17615383	0,742
6	17615409	0,742
6	17615420	0,742
6	17615482	0,742
6	17615489	0,742
6	17615576	0,742
6	17616194	0,742
6	17616227	0,742
6	17616268	0,742
6	17616321	0,742
6	17616391	0,742
6	17616434	0,742
6	17616463	0,742
6	17616468	0,742
6	17616624	0,742

6	17616851	0,742
6	17616910	0,742
6	17616963	0,742
6	17617366	0,742
6	17617951	0,742
6	17618039	0,742
6	17618264	0,742
6	17618713	0,742
6	17618839	0,742
6	17618850	0,742
6	17618997	0,742
6	17619156	0,742
6	17619177	0,742
6	17619585	0,742
6	17619689	0,742
6	17621302	0,742
6	25317172	0,649
6	25322358	0,649
6	25322443	0,622
6	25323028	0,622
6	25323464	0,622
6	25323540	0,622
6	25326026	0,649
6	25326461	0,649
6	25341050	0,570
6	25341664	0,622
6	25342160	0,622
6	25344472	0,649
6	25344669	0,622
6	25344784	0,649
6	25344813	0,596
6	25344850	0,622
6	25344900	0,622
6	25344962	0,622
6	25345051	0,649
6	25345053	0,649
6	25345076	0,622
6	25345137	0,649
6	25345189	0,649
6	25345205	0,622
6	25345220	0,622
6	25345248	0,649
6	25345250	0,622
6	25345356	0,649
6	25345420	0,649

Table S4. SNPs with allele-frequency difference > 0.5 in purging region in LW lineage on chromosome 7 (2.8-2.9 Mb).

Chromosome	Position	AFD
7	2834870	0,526
7	2835006	0,737
7	2835106	0,526
7	2835124	0,526
7	2835139	0,737
7	2835316	0,737
7	2835415	0,526
7	2835438	0,526
7	2835471	0,526
7	2835490	0,737
7	2835565	0,737
7	2835801	0,526
7	2836435	0,526
7	2855722	0,737
7	2855851	0,737
7	2856223	0,526
7	2856344	0,737
7	2856389	0,737
7	2856481	0,526
7	2856594	0,737
7	2856599	0,737
7	2856604	0,737
7	2856893	0,737
7	2856923	0,526
7	2856963	0,737
7	2856966	0,737
7	2856985	0,737
7	2856991	0,737
7	2856996	0,737
7	2857264	0,526
7	2857533	0,526
7	2857818	0,526
7	2858070	0,526
7	2858295	0,737
7	2950485	0,526
7	2950734	0,737
7	2952037	0,737
7	2954523	0,526
7	2954926	0,737
7	2954942	0,737
7	2954980	0,737
7	2954989	0,737
7	2955036	0,737
7	2955067	0,526

7	2955137	0,526
7	2955899	0,737
7	2956163	0,737
7	2956232	0,526
7	2956238	0,737
7	2956243	0,526
7	2956313	0,526
7	2956329	0,737
7	2958422	0,737
7	2976249	0,526
7	2976396	0,737

Table S5. SNPs with allele-frequency difference > 0.5 in purging region in LW lineage on chromosome 13 (3.2-3.5 Mb). Loci highlighted in green were tag SNPs selected by backward-elimination analysis in this region.

Chromosome	Position	AFD
13	3213191	0,632
13	3213197	0,632
13	3213235	0,632
13	3213239	0,632
13	3213477	0,632
13	3213671	0,632
13	3220658	0,632
13	3220811	0,632
13	3221898	0,632
13	3222399	0,632
13	3222413	0,632
13	3222508	0,632
13	3223469	0,632
13	3223501	0,632
13	3224029	0,632
13	3224142	0,632
13	3224168	0,632
13	3224194	0,632
13	3224206	0,632
13	3224207	0,632
13	3224208	0,632
13	3224210	0,632
13	3224298	0,632
13	3224651	0,632
13	3225579	0,632
13	3225695	0,632
13	3225727	0,632
13	3225767	0,632
13	3226449	0,632
13	3226472	0,632
13	3226596	0,632
13	3227204	0,632
13	3227578	0,632
13	3227692	0,632
13	3227698	0,632
13	3227703	0,632
13	3227722	0,632
13	3227731	0,632
13	3227751	0,632
13	3227883	0,632
13	3227892	0,632
13	3227948	0,632
13	3227950	0,632

13	3227962	0,632
13	3228170	0,632
13	3228202	0,632
13	3228542	0,632
13	3228559	0,632
13	3228592	0,632
13	3228605	0,632
13	3228626	0,632
13	3228806	0,632
13	3231098	0,632
13	3231291	0,632
13	3234458	0,632
13	3234502	0,632
13	3236100	0,632
13	3236632	0,632
13	3236988	0,632
13	3237366	0,632
13	3237855	0,632
13	3238664	0,632
13	3238673	0,632
13	3238751	0,632
13	3239106	0,632
13	3239147	0,632
13	3239172	0,632
13	3239190	0,632
13	3239215	0,632
13	3239219	0,632
13	3239286	0,632
13	3239304	0,632
13	3240054	0,632
13	3240162	0,632
13	3240279	0,632
13	3240321	0,632
13	3241013	0,632
13	3242053	0,632
13	3242321	0,632
13	3242391	0,632
13	3242433	0,632
13	3242494	0,632
13	3242653	0,632
13	3242736	0,632
13	3325423	0,605
13	3326758	0,605
13	3326767	0,605
13	3329507	0,684
13	3337244	0,658
13	3337726	0,579
13	3337734	0,579

13	3337753	0,579
13	3347092	0,579
13	3347439	0,658
13	3347590	0,658
13	3347614	0,658
13	3347628	0,658
13	3347688	0,579
13	3347726	0,658
13	3348091	0,579
13	3348208	0,658
13	3348506	0,658
13	3348508	0,658
13	3348581	0,658
13	3348970	0,579
13	3350060	0,658
13	3353445	0,579
13	3353466	0,579
13	3353795	0,579
13	3353798	0,579
13	3353948	0,579
13	3354238	0,579
13	3354892	0,658
13	3355489	0,500
13	3355563	0,579
13	3355765	0,658
13	3356743	0,658
13	3356995	0,579
13	3357094	0,579
13	3357220	0,500
13	3357697	0,500
13	3357753	0,658
13	3357762	0,658
13	3358256	0,500
13	3359343	0,658
13	3359345	0,658
13	3359718	0,500
13	3359821	0,658
13	3360471	0,658
13	3360524	0,500
13	3360548	0,500
13	3360951	0,658
13	3361180	0,658
13	3361181	0,500
13	3361293	0,658
13	3361361	0,658
13	3362190	0,658
13	3362225	0,500
13	3363491	0,658

13	3363947	0,658
13	3364678	0,658
13	3364862	0,658
13	3364988	0,658
13	3365031	0,658
13	3365107	0,658
13	3365464	0,658
13	3365698	0,658
13	3365949	0,579
13	3366828	0,579
13	3367290	0,579
13	3367627	0,658
13	3367632	0,658
13	3367701	0,658
13	3367704	0,579
13	3367741	0,658
13	3367805	0,579
13	3367982	0,579
13	3368035	0,658
13	3368070	0,658
13	3368227	0,658
13	3368479	0,658
13	3368560	0,658
13	3368734	0,658
13	3369505	0,579
13	3370418	0,500
13	3370585	0,658
13	3370662	0,658
13	3371096	0,500
13	3371521	0,579
13	3372080	0,658
13	3372224	0,658
13	3372953	0,658
13	3372976	0,500
13	3373005	0,500
13	3373013	0,500
13	3373382	0,579
13	3373537	0,658
13	3374186	0,658
13	3374387	0,500
13	3374451	0,658
13	3375075	0,500
13	3375146	0,579
13	3375212	0,579
13	3375509	0,658
13	3375525	0,579
13	3375635	0,579
13	3375638	0,579

13	3375704	0,658
13	3375963	0,579
13	3375967	0,579
13	3375979	0,658
13	3376813	0,500
13	3376872	0,658
13	3376932	0,579
13	3377000	0,658
13	3377379	0,658
13	3379438	0,579
13	3380432	0,579
13	3380585	0,500
13	3380842	0,500
13	3381156	0,658
13	3381282	0,500
13	3381288	0,500
13	3382033	0,500
13	3382052	0,658
13	3382105	0,579
13	3382216	0,500
13	3382239	0,500
13	3412966	0,658
13	3412984	0,658
13	3413003	0,658
13	3413007	0,658
13	3413022	0,658
13	3413025	0,658
13	3413843	0,500
13	3414160	0,500
13	3414287	0,500
13	3414384	0,500
13	3414716	0,500
13	3414726	0,500
13	3414797	0,658
13	3460317	0,579
13	3460453	0,579
13	3460715	0,579
13	3461384	0,579
13	3462159	0,579
13	3462216	0,579
13	3462221	0,579
13	3462229	0,579
13	3462455	0,579
13	3462646	0,579
13	3463308	0,579
13	3463320	0,579
13	3463352	0,579
13	3463553	0,579

13	3464217	0,579
13	3486910	0,658
13	3487821	0,579
13	3488285	0,579
13	3488581	0,658
13	3488652	0,579
13	3488755	0,579
13	3488873	0,658
13	3488912	0,579
13	3489237	0,579
13	3489331	0,579
13	3489628	0,658
13	3489697	0,579
13	3489718	0,579
13	3489762	0,579
13	3489841	0,579
13	3489900	0,579
13	3489901	0,579
13	3490132	0,658
13	3490135	0,579
13	3490167	0,579
13	3490317	0,579
13	3490380	0,658
13	3490406	0,579
13	3494738	0,579
13	3494817	0,658
13	3495425	0,658
13	3495522	0,658
13	3495558	0,579
13	3495641	0,658
13	3495648	0,632
13	3495663	0,632
13	3495684	0,632
13	3495685	0,632
13	3495688	0,632
13	3495757	0,632
13	3495828	0,553
13	3495901	0,632
13	3495920	0,632
13	3495933	0,632
13	3495937	0,632
13	3495954	0,632
13	3496043	0,553
13	3496467	0,553
13	3496981	0,632
13	3497011	0,632
13	3498304	0,632
13	3499683	0,658

13	3499834	0,658
13	3500241	0,553
13	3500839	0,553
13	3500854	0,553
13	3501401	0,632
13	3501443	0,632
13	3501809	0,632
13	3501880	0,632
13	3502009	0,632
13	3502021	0,632
13	3502450	0,632
13	3214343	0,697
13	3216765	0,697
13	3216784	0,697
13	3217631	0,697
13	3218011	0,697
13	3218461	0,697
13	3218781	0,697
13	3219016	0,697
13	3219120	0,697
13	3219165	0,697
13	3219321	0,697
13	3219327	0,697
13	3219336	0,697
13	3219609	0,697
13	3220096	0,697
13	3220230	0,697
13	3220543	0,697
13	3220605	0,697
13	3220898	0,697
13	3222356	0,697
13	3222375	0,697
13	3224114	0,697
13	3224135	0,697
13	3224185	0,697
13	3225323	0,697
13	3225373	0,697
13	3226886	0,697
13	3227756	0,697
13	3227849	0,697
13	3227866	0,697
13	3228129	0,697
13	3228299	0,697
13	3228300	0,697
13	3228838	0,697
13	3233670	0,697
13	3233780	0,697
13	3233816	0,697

13	3233930	0,697
13	3233950	0,697
13	3234455	0,697
13	3234524	0,697
13	3234579	0,697
13	3234586	0,697
13	3234613	0,697
13	3234745	0,697
13	3235677	0,697
13	3236231	0,697
13	3236602	0,697
13	3236898	0,697
13	3238486	0,697
13	3238608	0,697
13	3238635	0,697
13	3238640	0,697
13	3238696	0,697
13	3238749	0,697
13	3238767	0,697
13	3238786	0,697
13	3238855	0,697
13	3238886	0,697
13	3239318	0,697
13	3326432	0,597
13	3326688	0,597
13	3326707	0,597
13	3326792	0,597
13	3326835	0,597
13	3328272	0,597
13	3328377	0,597
13	3329158	0,597
13	3335016	0,597
13	3335018	0,597
13	3335153	0,597
13	3335577	0,597
13	3335818	0,597
13	3336218	0,597
13	3337199	0,597
13	3337245	0,597
13	3337476	0,597
13	3337627	0,597
13	3337705	0,597
13	3347646	0,597
13	3347770	0,597
13	3347781	0,597
13	3348291	0,597
13	3348767	0,597
13	3349106	0,597

13	3350055	0,597
13	3350123	0,597
13	3353510	0,597
13	3353520	0,597
13	3355697	0,597
13	3357510	0,597
13	3358025	0,597
13	3358730	0,597
13	3359136	0,597
13	3359178	0,597
13	3359181	0,597
13	3359199	0,597
13	3359312	0,597
13	3359563	0,597
13	3360174	0,597
13	3360285	0,597
13	3360388	0,597
13	3360801	0,597
13	3361013	0,597
13	3361190	0,597
13	3361263	0,597
13	3362280	0,597
13	3363494	0,597
13	3364449	0,597
13	3364531	0,597
13	3365041	0,597
13	3368410	0,597
13	3370110	0,597
13	3370115	0,597
13	3370533	0,597
13	3370609	0,597
13	3370615	0,597
13	3370806	0,597
13	3371012	0,597
13	3371676	0,597
13	3371902	0,597
13	3372287	0,597
13	3372875	0,597
13	3372894	0,597
13	3373102	0,597
13	3374039	0,597
13	3374131	0,597
13	3374365	0,597
13	3374370	0,597
13	3376080	0,597
13	3376589	0,597
13	3377004	0,597
13	3377027	0,597

13	3377222	0,597
13	3377885	0,597
13	3377894	0,597
13	3377911	0,597
13	3377973	0,597
13	3377986	0,597
13	3378008	0,597
13	3378013	0,597
13	3378029	0,597
13	3379152	0,597
13	3379245	0,597
13	3379273	0,597
13	3379352	0,597
13	3379381	0,597
13	3379443	0,597
13	3381718	0,597
13	3381720	0,597
13	3382265	0,597
13	3382414	0,597
13	3413030	0,597
13	3414169	0,597
13	3414194	0,597
13	3414310	0,597
13	3414517	0,597
13	3414713	0,597
13	3414875	0,597
13	3414912	0,597
13	3460399	0,597
13	3460408	0,597
13	3460535	0,597
13	3460811	0,597
13	3460876	0,597
13	3461264	0,797
13	3461778	0,797
13	3462378	0,797
13	3462573	0,797
13	3463013	0,797
13	3463876	0,797
13	3464248	0,797
13	3464425	0,797
13	3464439	0,797
13	3464453	0,797
13	3464494	0,797
13	3464603	0,797
13	3464870	0,797
13	3464895	0,797
13	3464949	0,797
13	3465014	0,797

13	3465251	0,797
13	3465253	0,797
13	3465315	0,797
13	3465518	0,797
13	3465836	0,797
13	3465914	0,797
13	3466003	0,797
13	3466133	0,797
13	3466272	0,797
13	3466361	0,797
13	3466412	0,797
13	3466729	0,797
13	3466730	0,797
13	3466758	0,797
13	3467068	0,797
13	3467387	0,797
13	3467474	0,797
13	3467793	0,797
13	3467805	0,797
13	3467906	0,797
13	3468125	0,797
13	3468206	0,797
13	3468258	0,797
13	3468277	0,797
13	3468396	0,797
13	3468729	0,797
13	3468858	0,797
13	3469135	0,797
13	3469238	0,797
13	3469298	0,797
13	3469348	0,797
13	3469386	0,797
13	3469399	0,797
13	3469408	0,797
13	3469411	0,797
13	3469456	0,797
13	3469528	0,797
13	3469557	0,797
13	3469623	0,797
13	3469647	0,797
13	3469693	0,797
13	3469784	0,797
13	3469800	0,797
13	3469942	0,797
13	3469982	0,797
13	3469993	0,797
13	3470162	0,797
13	3470192	0,797

13	3470340	0,797
13	3470354	0,797
13	3470496	0,797
13	3470719	0,797
13	3470870	0,797
13	3470879	0,797
13	3470911	0,797
13	3471110	0,797
13	3471125	0,797
13	3471126	0,797
13	3471232	0,797
13	3471587	0,797
13	3471612	0,797
13	3471627	0,797
13	3472751	0,797
13	3472999	0,797
13	3473153	0,797
13	3473154	0,797
13	3473287	0,797
13	3473523	0,797
13	3473649	0,797
13	3473679	0,797
13	3473681	0,797
13	3473764	0,797
13	3473866	0,797
13	3473962	0,797
13	3474083	0,797
13	3474157	0,797
13	3474164	0,797
13	3474197	0,797
13	3474600	0,797
13	3474606	0,797
13	3474650	0,797
13	3474723	0,797
13	3475118	0,797
13	3475123	0,797
13	3475220	0,797
13	3475237	0,797
13	3475243	0,797
13	3475258	0,797
13	3475394	0,797
13	3475613	0,797
13	3475618	0,797
13	3476052	0,797
13	3476184	0,797
13	3476195	0,797
13	3476237	0,797
13	3476276	0,797

13	3476277	0,797
13	3476278	0,797
13	3476299	0,797
13	3476377	0,797
13	3476576	0,797
13	3476638	0,797
13	3477121	0,797
13	3477146	0,797
13	3477222	0,797
13	3477258	0,797
13	3477261	0,797
13	3477271	0,797
13	3477278	0,797
13	3477338	0,797
13	3477394	0,797
13	3477442	0,797
13	3477474	0,797
13	3477538	0,797
13	3477539	0,797
13	3477556	0,797
13	3477563	0,797
13	3477567	0,797
13	3477574	0,797
13	3477578	0,797
13	3477778	0,797
13	3477782	0,797
13	3477806	0,797
13	3477808	0,797
13	3477819	0,797
13	3477833	0,797
13	3477845	0,797
13	3477912	0,797
13	3477951	0,797
13	3477964	0,797
13	3477966	0,797
13	3478023	0,797
13	3478056	0,797
13	3478104	0,797
13	3478226	0,797
13	3478406	0,797
13	3478517	0,797
13	3478551	0,797
13	3478674	0,797
13	3479086	0,797
13	3479145	0,797
13	3479263	0,797
13	3479277	0,797
13	3479288	0,797

13	3479411	0,797
13	3479437	0,797
13	3479868	0,797
13	3480022	0,797
13	3480045	0,797
13	3480171	0,797
13	3480228	0,797
13	3480245	0,797
13	3480584	0,797
13	3482134	0,797
13	3482309	0,797
13	3482420	0,797
13	3482451	0,797
13	3482554	0,797
13	3482577	0,797
13	3482591	0,797
13	3482732	0,797
13	3482734	0,797
13	3482739	0,797
13	3482791	0,797
13	3482809	0,797
13	3482820	0,797
13	3482837	0,797
13	3482867	0,797
13	3482918	0,797
13	3482920	0,797
13	3482937	0,797
13	3482952	0,797
13	3483033	0,797
13	3483090	0,797
13	3483199	0,797
13	3483349	0,797
13	3483403	0,797
13	3483490	0,797
13	3483500	0,797
13	3483514	0,797
13	3483579	0,797
13	3483642	0,797
13	3483791	0,797
13	3483912	0,797
13	3483923	0,797
13	3483950	0,797
13	3483958	0,797
13	3484275	0,797
13	3484355	0,797
13	3484363	0,797
13	3484369	0,797
13	3484424	0,797

13	3484616	0,797
13	3484967	0,797
13	3485026	0,797
13	3485636	0,797
13	3485895	0,797
13	3487527	0,797
13	3487615	0,797
13	3487628	0,797
13	3487865	0,797
13	3488389	0,797
13	3488397	0,797
13	3488491	0,797
13	3488499	0,797
13	3488613	0,797
13	3492844	0,797
13	3493239	0,797
13	3493247	0,797
13	3493261	0,797
13	3493465	0,797
13	3493647	0,797
13	3493779	0,797
13	3493813	0,797
13	3493907	0,797
13	3493931	0,797
13	3493989	0,797
13	3493992	0,797
13	3494017	0,797
13	3494873	0,797
13	3495619	0,797
13	3496492	0,797
13	3496537	0,797
13	3496636	0,797
13	3496641	0,797
13	3496831	0,797
13	3497080	0,797
13	3497849	0,797
13	3497865	0,797
13	3498418	0,797
13	3498597	0,797
13	3498679	0,797
13	3499266	0,797
13	3499362	0,797
13	3501429	0,797
13	3501479	0,797
13	3501498	0,797
13	3501629	0,797
13	3502332	0,797
13	3502475	0,797

Table S6. SNPs with allele-frequency difference > 0.5 in purging region in HW lineage on chromosome 2 (16.0-17.4 Mb). Loci highlighted in green were tag SNPs selected by backward-elimination analysis in this region.

Chromosome	Position	AFD
2	16018852	0,672
2	16019289	0,672
2	16020599	0,689
2	16020895	0,672
2	16020922	0,672
2	16021762	0,672
2	16021783	0,672
2	16021792	0,672
2	16021793	0,672
2	16021851	0,672
2	16022191	0,672
2	16022228	0,672
2	16022264	0,672
2	16022828	0,689
2	16022839	0,689
2	16024874	0,672
2	16025604	0,672
2	16025626	0,672
2	16025743	0,672
2	16025787	0,672
2	16025969	0,672
2	16027339	0,672
2	16027442	0,689
2	16027625	0,672
2	16028657	0,689
2	16029346	0,672
2	16029370	0,672
2	16030889	0,689
2	16032604	0,672
2	16032743	0,672
2	16033155	0,672
2	16033705	0,689
2	16034127	0,672
2	16034422	0,672
2	16034950	0,689
2	16035707	0,689
2	16036504	0,672
2	16038971	0,689
2	16039019	0,689
2	16039832	0,689
2	16040684	0,672
2	16040713	0,672
2	16040714	0,672
2	16042927	0,672

2	16042928	0,672
2	16043162	0,689
2	16043261	0,689
2	16044373	0,689
2	16044606	0,689
2	16044997	0,689
2	16045079	0,672
2	16045121	0,672
2	16045673	0,672
2	16047558	0,689
2	16047582	0,689
2	16048473	0,672
2	16048503	0,672
2	16048699	0,672
2	16048757	0,672
2	16048779	0,672
2	16048849	0,672
2	16048893	0,672
2	16048925	0,672
2	16048952	0,672
2	16048977	0,672
2	16049063	0,672
2	16049090	0,672
2	16049122	0,672
2	16049542	0,672
2	16049549	0,672
2	16049748	0,672
2	16049751	0,672
2	16049859	0,672
2	16050024	0,672
2	16050030	0,672
2	16050040	0,672
2	16050156	0,672
2	16050968	0,672
2	16050976	0,672
2	16051399	0,672
2	16051411	0,672
2	16051452	0,672
2	16051457	0,672
2	16051823	0,672
2	16052783	0,689
2	16052886	0,672
2	16052915	0,672
2	16052940	0,672
2	16052962	0,672
2	16053024	0,672
2	16053025	0,672
2	16053540	0,672

2	16053581	0,689
2	16053695	0,689
2	16053884	0,672
2	16053902	0,672
2	16054300	0,672
2	16054568	0,672
2	16054577	0,672
2	16054688	0,672
2	16054696	0,672
2	16055066	0,689
2	16055319	0,672
2	16055490	0,672
2	16055648	0,672
2	16055649	0,672
2	16056105	0,672
2	16056196	0,672
2	16056311	0,672
2	16056314	0,672
2	16057729	0,672
2	16057732	0,672
2	16058018	0,672
2	16058413	0,672
2	16058417	0,672
2	16058850	0,672
2	16058851	0,672
2	16059696	0,672
2	16059787	0,672
2	16059955	0,689
2	16060826	0,672
2	16060834	0,672
2	16060989	0,672
2	16061081	0,672
2	16061102	0,672
2	16061246	0,672
2	16061333	0,672
2	16061439	0,672
2	16061464	0,672
2	16061474	0,672
2	16061904	0,672
2	16062487	0,672
2	16062491	0,672
2	16063083	0,689
2	16063798	0,672
2	16063831	0,689
2	16063860	0,689
2	16065479	0,672
2	16065597	0,672
2	16065649	0,672

2	16067646	0,689
2	16068314	0,689
2	16069080	0,672
2	16069989	0,672
2	16072407	0,672
2	16074026	0,689
2	16076012	0,672
2	16076604	0,689
2	16076666	0,672
2	16076981	0,689
2	16077217	0,672
2	16077218	0,672
2	16077494	0,689
2	16078419	0,689
2	16078952	0,689
2	16079439	0,689
2	16079455	0,689
2	16079635	0,689
2	16080405	0,672
2	16080598	0,689
2	16082538	0,672
2	16082565	0,672
2	16082601	0,672
2	16082608	0,672
2	16083329	0,672
2	16083442	0,672
2	16083443	0,672
2	16084943	0,672
2	16084968	0,672
2	16085044	0,672
2	16085102	0,672
2	16085418	0,689
2	16085660	0,689
2	16085803	0,672
2	16094132	0,689
2	16094161	0,689
2	16130390	0,672
2	16130395	0,672
2	16130405	0,672
2	16130407	0,672
2	16130990	0,672
2	16131145	0,689
2	16131239	0,689
2	16131302	0,689
2	16131354	0,689
2	16131372	0,689
2	16131407	0,689
2	16131792	0,689

2	16131832	0,689
2	16131878	0,689
2	16131937	0,672
2	16131945	0,672
2	16132931	0,672
2	16133009	0,672
2	16133141	0,672
2	16133351	0,672
2	16133462	0,689
2	16133502	0,689
2	16133720	0,672
2	16133722	0,672
2	16133860	0,672
2	16134183	0,672
2	16134940	0,672
2	16134948	0,672
2	16135076	0,672
2	16135117	0,672
2	16135567	0,689
2	16136504	0,689
2	16163499	0,672
2	16163993	0,672
2	16164047	0,672
2	16164199	0,672
2	16164201	0,672
2	16164213	0,672
2	16164360	0,672
2	16164364	0,672
2	16164463	0,689
2	16164533	0,689
2	16164750	0,689
2	16164773	0,689
2	16164774	0,689
2	16165241	0,689
2	16165306	0,689
2	16165378	0,672
2	16167770	0,689
2	16168501	0,689
2	16168546	0,672
2	16168558	0,672
2	16169682	0,672
2	16169746	0,672
2	16170191	0,672
2	16170335	0,672
2	16170346	0,672
2	16170358	0,672
2	16170399	0,672
2	16170540	0,672

2	16170572	0,672
2	16170615	0,672
2	16170700	0,672
2	16170738	0,672
2	16171001	0,689
2	16171207	0,689
2	16171242	0,689
2	16194720	0,672
2	16195714	0,689
2	16196801	0,689
2	16196895	0,672
2	16196934	0,672
2	16197837	0,689
2	16197845	0,689
2	16198210	0,689
2	16198475	0,672
2	16198496	0,672
2	16198520	0,672
2	16198599	0,672
2	16198681	0,672
2	16198708	0,672
2	16198883	0,672
2	16199700	0,689
2	16199750	0,672
2	16199791	0,672
2	16200247	0,689
2	16200248	0,689
2	16201797	0,672
2	16201818	0,672
2	16201860	0,689
2	16201971	0,672
2	16203302	0,689
2	16204292	0,689
2	16204435	0,672
2	16204623	0,689
2	16204685	0,689
2	16204708	0,689
2	16204714	0,689
2	16205054	0,689
2	16205986	0,689
2	16207454	0,672
2	16209050	0,672
2	16209138	0,689
2	16209202	0,689
2	16209354	0,672
2	16209355	0,672
2	16211118	0,689
2	16215251	0,672

2	16215506	0,672
2	16215514	0,672
2	16215515	0,672
2	16215521	0,672
2	16215540	0,672
2	16215574	0,672
2	16215578	0,672
2	16215751	0,672
2	16215990	0,672
2	16216352	0,672
2	16216513	0,672
2	16216600	0,689
2	16217635	0,689
2	16219101	0,672
2	16219722	0,689
2	16221016	0,689
2	16222281	0,689
2	16222323	0,689
2	16222364	0,689
2	16222371	0,689
2	16222595	0,672
2	16223718	0,672
2	16224060	0,672
2	16224109	0,672
2	16224308	0,672
2	16224521	0,672
2	16224936	0,672
2	16224937	0,672
2	16225000	0,689
2	16225400	0,689
2	16225728	0,672
2	16225730	0,672
2	16225731	0,672
2	16225886	0,689
2	16225939	0,689
2	16226313	0,672
2	16226316	0,672
2	16226334	0,672
2	16226352	0,672
2	16226874	0,672
2	16226982	0,672
2	16227150	0,689
2	16228770	0,689
2	16228854	0,689
2	16229632	0,689
2	16230834	0,689
2	16230925	0,689
2	16230991	0,689

2	16231040	0,689
2	16231043	0,689
2	16231265	0,689
2	16232847	0,672
2	16233029	0,689
2	16233042	0,689
2	16233417	0,689
2	16233419	0,689
2	16233478	0,689
2	16233547	0,689
2	16233679	0,689
2	16234103	0,672
2	16234458	0,672
2	16234797	0,689
2	16234989	0,672
2	16234998	0,672
2	16235005	0,672
2	16235007	0,672
2	16235050	0,672
2	16235087	0,672
2	16235508	0,689
2	16235721	0,672
2	16236593	0,672
2	16237466	0,689
2	16238772	0,689
2	16238824	0,689
2	16238884	0,689
2	16239125	0,689
2	16239258	0,689
2	16239314	0,672
2	16239322	0,672
2	16239818	0,689
2	16240842	0,672
2	16240865	0,672
2	16241694	0,689
2	16241739	0,689
2	16241954	0,672
2	16242004	0,672
2	16242639	0,689
2	16242652	0,689
2	16242792	0,672
2	16242800	0,672
2	16242806	0,672
2	16242822	0,672
2	16242823	0,672
2	16243066	0,689
2	16243138	0,689
2	16243348	0,689

2	16243389	0,689
2	16243408	0,689
2	16244208	0,689
2	16244250	0,689
2	16244627	0,689
2	16246276	0,672
2	16246583	0,689
2	16246758	0,672
2	16246813	0,672
2	16246830	0,672
2	16246959	0,672
2	16247124	0,689
2	16247126	0,689
2	16247484	0,672
2	16247485	0,672
2	16247664	0,672
2	16247767	0,672
2	16249256	0,672
2	16249403	0,672
2	16249433	0,672
2	16249601	0,672
2	16249602	0,672
2	16249801	0,672
2	16249849	0,672
2	16249973	0,672
2	16250091	0,672
2	16251083	0,672
2	16251091	0,672
2	16251093	0,672
2	16270943	0,689
2	16272471	0,689
2	16273523	0,672
2	16275109	0,689
2	16281315	0,689
2	16281532	0,689
2	16281614	0,689
2	16285291	0,672
2	16285311	0,689
2	16291813	0,689
2	16292123	0,689
2	16292230	0,672
2	16292352	0,689
2	16297518	0,672
2	16298142	0,689
2	16299329	0,714
2	16707887	0,664
2	16709817	0,664
2	16710654	0,664

2	16711259	0,664
2	16711410	0,664
2	16711630	0,664
2	16712549	0,664
2	16713106	0,664
2	16713107	0,664
2	16713980	0,689
2	16713993	0,689
2	16754407	0,664
2	16754437	0,664
2	16754447	0,664
2	16754477	0,664
2	16754546	0,664
2	16754631	0,664
2	16754764	0,664
2	16755107	0,664
2	16755258	0,664
2	16756213	0,664
2	16756385	0,664
2	16756500	0,664
2	16757980	0,664
2	16758396	0,664
2	16758482	0,664
2	16758513	0,664
2	16758627	0,664
2	16758674	0,664
2	16758680	0,664
2	16758692	0,664
2	16758708	0,664
2	16758748	0,664
2	16758845	0,664
2	16758873	0,664
2	16758883	0,664
2	16759090	0,664
2	16759093	0,664
2	16759100	0,664
2	16759101	0,664
2	16759441	0,664
2	16759453	0,664
2	16759475	0,664
2	16759527	0,664
2	16759686	0,664
2	16759702	0,664
2	16759981	0,664
2	16760100	0,664
2	16760215	0,664
2	16760235	0,664
2	16760255	0,664

2	16760379	0,664
2	16760484	0,664
2	16760504	0,664
2	16760549	0,664
2	16760559	0,664
2	16760672	0,664
2	16760790	0,664
2	16760854	0,664
2	16760951	0,664
2	16760976	0,664
2	16761023	0,664
2	16761075	0,664
2	16761083	0,664
2	16761088	0,664
2	16761172	0,664
2	16761780	0,664
2	16761922	0,664
2	16762011	0,664
2	16762342	0,664
2	16762412	0,664
2	16762510	0,664
2	16762537	0,664
2	16762548	0,664
2	16762558	0,664
2	16762570	0,664
2	16762577	0,664
2	16762591	0,664
2	16762863	0,664
2	16763215	0,664
2	16763220	0,664
2	16763268	0,664
2	16764005	0,664
2	16764243	0,664
2	16764364	0,664
2	16764526	0,664
2	16764708	0,664
2	16764826	0,664
2	16765509	0,664
2	16765968	0,664
2	16766165	0,664
2	16766515	0,664
2	16766701	0,664
2	16766718	0,664
2	16767005	0,664
2	16767163	0,664
2	16767405	0,664
2	16767438	0,664
2	16767488	0,664

2	16767616	0,664
2	16768306	0,664
2	16769069	0,664
2	16769948	0,664
2	16770018	0,664
2	16770074	0,664
2	16770114	0,664
2	16770159	0,664
2	16770174	0,664
2	16770186	0,664
2	16770212	0,664
2	16770253	0,664
2	16770257	0,664
2	16770377	0,664
2	16770456	0,664
2	16770750	0,664
2	16770756	0,664
2	16770780	0,664
2	16771011	0,664
2	16771179	0,664
2	16771253	0,664
2	16771294	0,664
2	16771296	0,664
2	16771302	0,664
2	16771326	0,664
2	16771409	0,664
2	16771434	0,664
2	16771538	0,664
2	16771797	0,664
2	16772121	0,664
2	16772308	0,664
2	16772315	0,664
2	16772555	0,664
2	16772756	0,664
2	16773329	0,664
2	16773716	0,664
2	16774290	0,664
2	16774380	0,664
2	16774400	0,664
2	16774603	0,664
2	16774882	0,664
2	16775332	0,664
2	16775352	0,664
2	16775380	0,664
2	16778485	0,664
2	16778526	0,664
2	16778547	0,664
2	16778548	0,664

2	16778568	0,664
2	16778582	0,664
2	16778588	0,664
2	16778605	0,664
2	16778662	0,664
2	16778785	0,664
2	16778869	0,664
2	16778990	0,664
2	16779094	0,664
2	16779123	0,664
2	16779474	0,664
2	16779490	0,664
2	16779599	0,664
2	16779601	0,664
2	16779603	0,664
2	16779641	0,664
2	16779682	0,664
2	16779895	0,664
2	16780121	0,664
2	16780125	0,664
2	16780247	0,664
2	16780249	0,664
2	16780356	0,664
2	16780521	0,664
2	16780568	0,664
2	16780891	0,664
2	16781165	0,664
2	16781190	0,664
2	16781239	0,664
2	16781533	0,664
2	16781573	0,664
2	16781856	0,664
2	16781962	0,664
2	16782057	0,664
2	16782075	0,664
2	16782077	0,664
2	16782315	0,664
2	16782702	0,664
2	16782749	0,664
2	16782863	0,664
2	16782922	0,664
2	16782969	0,664
2	16783613	0,664
2	16783705	0,664
2	16783798	0,664
2	16783940	0,664
2	16783951	0,664
2	16783995	0,664

2	16784172	0,664
2	16784291	0,664
2	16784339	0,664
2	16784458	0,664
2	16784548	0,664
2	16784552	0,664
2	16784694	0,664
2	16784742	0,664
2	16785336	0,664
2	16785360	0,664
2	16785700	0,664
2	16785891	0,664
2	16785954	0,664
2	16786152	0,664
2	16786204	0,664
2	16786382	0,664
2	16786593	0,664
2	16786754	0,664
2	16840452	0,689
2	16840463	0,689
2	16840707	0,689
2	16841064	0,689
2	16841458	0,689
2	16841810	0,689
2	16842001	0,689
2	16842004	0,689
2	16842008	0,689
2	16842045	0,689
2	16842134	0,689
2	16843710	0,689
2	16843822	0,689
2	16844136	0,689
2	16844153	0,689
2	16844408	0,689
2	16844776	0,689
2	16863220	0,689
2	16863950	0,689
2	16864276	0,689
2	16864295	0,689
2	16864296	0,689
2	16864363	0,689
2	16864386	0,689
2	16864395	0,689
2	16864410	0,689
2	16864438	0,689
2	16864477	0,689
2	16865341	0,689
2	16865366	0,689

2	16865415	0,689
2	16865453	0,689
2	16865498	0,689
2	16877920	0,689
2	16878003	0,689
2	16878046	0,689
2	16878374	0,689
2	16878734	0,689
2	16881224	0,689
2	16881308	0,689
2	16882417	0,689
2	16882514	0,689
2	16883210	0,689
2	16883276	0,689
2	16883278	0,689
2	16883331	0,689
2	16883502	0,689
2	16883520	0,689
2	16883678	0,689
2	16883742	0,689
2	16883784	0,689
2	16883886	0,689
2	16883990	0,689
2	16884061	0,689
2	16884285	0,689
2	16884421	0,689
2	16884430	0,689
2	16884506	0,689
2	16884600	0,689
2	17388396	0,690
2	17389835	0,690
2	17389928	0,690
2	17390100	0,690
2	17390255	0,690
2	17390441	0,690
2	17391044	0,690
2	17391055	0,690
2	17391165	0,690
2	17391372	0,690
2	17393443	0,690
2	17394070	0,690
2	17395177	0,690
2	17395579	0,690
2	17396199	0,690
2	17397146	0,690
2	17397197	0,690
2	17397794	0,690
2	17397940	0,690

2	17397974	0,690
2	17397976	0,690
2	17397987	0,690
2	17398003	0,690
2	17398048	0,690
2	17398171	0,690
2	17398306	0,690
2	17398336	0,690
2	17398349	0,690
2	17398577	0,690
2	17398671	0,690
2	17398675	0,690
2	17398747	0,690
2	17398749	0,690
2	17398754	0,690
2	17399001	0,690
2	17399210	0,690
2	17399367	0,690
2	17401104	0,690
2	17401840	0,690
2	17403266	0,690
2	17403478	0,690
2	17404921	0,690
2	17405168	0,690
2	17405495	0,690
2	17406098	0,690
2	17406484	0,690
2	17406486	0,690
2	17407984	0,690
2	17408413	0,690
2	17410792	0,690
2	17411152	0,690
2	17411221	0,690
2	17411434	0,690
2	17411730	0,690
2	17414092	0,690
2	17417392	0,690
2	17417685	0,690
2	17424838	0,690
2	17425441	0,690
2	17425485	0,690
2	17425550	0,690
2	17426111	0,690
2	17426174	0,690
2	17426370	0,690
2	17426385	0,690
2	17426805	0,690
2	17427150	0,690

2	17427366	0,690
2	17431383	0,690
2	17431625	0,690
2	17431874	0,690

Table S7. SNPs with allele-frequency difference > 0.5 in purging region in HW lineage on chromosome 4 (35.1-35.2 Mb). The locus highlighted in green was the tag SNP selected by backward-elimination analysis in this region.

Chromosome	Position	AFD
4	35195793	0,550
4	35196062	0,550
4	35196063	0,550
4	35196119	0,550
4	35196213	0,550
4	35196228	0,550
4	35198689	0,550
4	35199924	0,550
4	35200416	0,550
4	35202172	0,550
4	35202175	0,550
4	35202207	0,550
4	35202215	0,550
4	35202524	0,550
4	35202691	0,550
4	35202935	0,550
4	35202989	0,550
4	35205936	0,550
4	35206475	0,550
4	35206478	0,550
4	35206497	0,550
4	35206801	0,550
4	35207051	0,550
4	35207197	0,550
4	35207461	0,550
4	35207916	0,550
4	35208148	0,550
4	35211807	0,550
4	35211968	0,550
4	35211974	0,550
4	35213778	0,550
4	35214705	0,550
4	35214721	0,550
4	35214786	0,550
4	35214994	0,550
4	35215261	0,550
4	35217632	0,550
4	35218252	0,550
4	35219182	0,550
4	35219411	0,550
4	35219636	0,550
4	35223201	0,550
4	35223690	0,550
4	35223707	0,550

4	35223716	0,550
4	35223717	0,550
4	35224320	0,550
4	35225429	0,550
4	35226658	0,550

Table S8. SNPs with allele-frequency difference > 0.5 in purging region in HW lineage on chromosome 8 (2.3-2.6 Mb). Loci highlighted in green were tag SNPs selected by backward-elimination analysis in this region.

Chromosome	Position	AFD
8	2352132	0,722
8	2352760	0,722
8	2352967	0,704
8	2354140	0,704
8	2354158	0,704
8	2354329	0,704
8	2354340	0,704
8	2354654	0,704
8	2354680	0,704
8	2354871	0,704
8	2354881	0,704
8	2355218	0,722
8	2355368	0,704
8	2355435	0,722
8	2355446	0,722
8	2355533	0,704
8	2356259	0,722
8	2357621	0,687
8	2357625	0,687
8	2358845	0,722
8	2359118	0,704
8	2359119	0,704
8	2359837	0,722
8	2359983	0,722
8	2360084	0,722
8	2360138	0,704
8	2360145	0,704
8	2360181	0,722
8	2360304	0,722
8	2360472	0,722
8	2360473	0,722
8	2360734	0,722
8	2365486	0,704
8	2365532	0,704
8	2365921	0,704
8	2368003	0,704
8	2368038	0,704
8	2375580	0,722
8	2375595	0,722
8	2376928	0,722
8	2377305	0,722
8	2377321	0,722
8	2378216	0,704
8	2378520	0,722

8	2378531	0,722
8	2382995	0,704
8	2383079	0,704
8	2383085	0,704
8	2383537	0,722
8	2383776	0,704
8	2383945	0,704
8	2384229	0,704
8	2385169	0,704
8	2385997	0,722
8	2386182	0,704
8	2386402	0,704
8	2389406	0,722
8	2389433	0,722
8	2389951	0,704
8	2391000	0,704
8	2391061	0,704
8	2391755	0,704
8	2391756	0,704
8	2392059	0,704
8	2392965	0,704
8	2393051	0,722
8	2393107	0,722
8	2393831	0,704
8	2393832	0,704
8	2393859	0,704
8	2394405	0,704
8	2394420	0,704
8	2394475	0,704
8	2395264	0,704
8	2395677	0,704
8	2395700	0,704
8	2395748	0,722
8	2395759	0,722
8	2395788	0,722
8	2395867	0,722
8	2395919	0,704
8	2395927	0,704
8	2396003	0,704
8	2396287	0,722
8	2396310	0,722
8	2396356	0,722
8	2396918	0,722
8	2397074	0,722
8	2397121	0,722
8	2397234	0,704
8	2397406	0,722
8	2398504	0,722

8	2398509	0,722
8	2399529	0,704
8	2401770	0,722
8	2401866	0,722
8	2402059	0,722
8	2402388	0,704
8	2403479	0,704
8	2403493	0,704
8	2403522	0,704
8	2403546	0,704
8	2403588	0,704
8	2403616	0,722
8	2403693	0,722
8	2404692	0,704
8	2404795	0,704
8	2405241	0,704
8	2405272	0,722
8	2405351	0,722
8	2405550	0,722
8	2405586	0,722
8	2405769	0,704
8	2405811	0,704
8	2406448	0,722
8	2407321	0,704
8	2407727	0,722
8	2407761	0,722
8	2408362	0,704
8	2409105	0,722
8	2410302	0,704
8	2410756	0,704
8	2410989	0,704
8	2411950	0,722
8	2412253	0,704
8	2412397	0,704
8	2412399	0,704
8	2412438	0,704
8	2415152	0,704
8	2415172	0,704
8	2415393	0,722
8	2416217	0,704
8	2416404	0,729
8	2416439	0,704
8	2416451	0,704
8	2416455	0,704
8	2416457	0,704
8	2417630	0,704
8	2417654	0,704
8	2417655	0,704

8	2418234	0,722
8	2418665	0,704
8	2419373	0,722
8	2419641	0,704
8	2419860	0,704
8	2419888	0,704
8	2423201	0,729
8	2423203	0,729
8	2423204	0,729
8	2423206	0,729
8	2423209	0,729
8	2423212	0,729
8	2423214	0,729
8	2423215	0,729
8	2423217	0,729
8	2424465	0,729
8	2424816	0,729
8	2424820	0,729
8	2424928	0,747
8	2424975	0,747
8	2425201	0,747
8	2425232	0,747
8	2425233	0,747
8	2425235	0,747
8	2425244	0,747
8	2425258	0,747
8	2425259	0,747
8	2425267	0,747
8	2425316	0,747
8	2425797	0,729
8	2426006	0,747
8	2426115	0,729
8	2426635	0,729
8	2426764	0,747
8	2428319	0,729
8	2429762	0,729
8	2432221	0,729
8	2433788	0,747
8	2434675	0,747
8	2435758	0,747
8	2435925	0,729
8	2435926	0,729
8	2435937	0,729
8	2435958	0,729
8	2436025	0,729
8	2441087	0,729
8	2441088	0,729
8	2442528	0,712

8	2443111	0,747
8	2443734	0,747
8	2465954	0,747
8	2468455	0,747
8	2468806	0,729
8	2469825	0,747
8	2471323	0,729
8	2472553	0,729
8	2474293	0,747
8	2474322	0,747
8	2476990	0,747
8	2477382	0,747
8	2478394	0,729
8	2478446	0,729
8	2482154	0,722
8	2482831	0,729
8	2482941	0,729
8	2483069	0,729
8	2483757	0,704
8	2484651	0,722
8	2485395	0,729
8	2485396	0,729
8	2485881	0,729
8	2486249	0,722
8	2487971	0,729
8	2487976	0,729
8	2487980	0,729
8	2487991	0,729
8	2487998	0,729
8	2619778	0,597
8	2620204	0,679
8	2620210	0,679
8	2620314	0,597
8	2620494	0,654
8	2620787	0,597
8	2623801	0,654
8	2625125	0,597
8	2628282	0,679
8	2628294	0,679
8	2628816	0,597
8	2629287	0,679
8	2630483	0,597
8	2631782	0,729
8	2631919	0,679
8	2632180	0,654
8	2633954	0,597
8	2634120	0,597
8	2635044	0,704

8	2636526	0,597
8	2636785	0,597
8	2637272	0,729
8	2643498	0,679
8	2643504	0,754
8	2644173	0,679
8	2644504	0,704
8	2644949	0,729
8	2644983	0,754
8	2645124	0,597
8	2647446	0,754
8	2647882	0,754
8	2649343	0,597
8	2649393	0,647
8	2650075	0,704
8	2650398	0,704
8	2650543	0,622
8	2650552	0,597
8	2650903	0,729
8	2652329	0,679
8	2654071	0,622
8	2654993	0,622
8	2655085	0,597
8	2655594	0,597
8	2657644	0,597
8	2659396	0,597
8	2663026	0,679
8	2663732	0,754
8	2664290	0,679
8	2668676	0,729
8	2668786	0,597
8	2668916	0,597
8	2668917	0,597
8	2668953	0,622
8	2670676	0,629
8	2671025	0,597
8	2671441	0,754
8	2675858	0,597
8	2679419	0,604
8	2679420	0,679
8	2680861	0,597
8	2684657	0,597

Table S9. SNPs with allele-frequency difference > 0.5 in purging region in HW lineage on chromosome 9 (11.9-13.6 Mb). Loci highlighted in green were tag SNPs selected by backward-elimination analysis in this region.

Chromosome	Position	AFD
9	11962587	0,638
9	11962697	0,638
9	11962709	0,638
9	11962741	0,638
9	11962955	0,638
9	11963880	0,638
9	11966906	0,638
9	11967495	0,638
9	11967556	0,638
9	11968082	0,638
9	11968232	0,638
9	11968895	0,638
9	11968997	0,638
9	11969562	0,638
9	11969846	0,638
9	11969858	0,638
9	11969976	0,638
9	11970019	0,638
9	11970430	0,638
9	11970652	0,638
9	11970862	0,638
9	11971094	0,638
9	11971516	0,638
9	11971635	0,638
9	11973150	0,638
9	11973647	0,638
9	11974656	0,638
9	11976410	0,638
9	11976709	0,638
9	11976769	0,638
9	11977385	0,638
9	11977446	0,638
9	11978974	0,638
9	11979086	0,638
9	11979348	0,638
9	11979569	0,638
9	11979594	0,638
9	11979601	0,638
9	11979613	0,638
9	11979643	0,638
9	11979678	0,638
9	11979936	0,638
9	11980166	0,638
9	11980239	0,638

9	11980346	0,638
9	11981123	0,638
9	11981562	0,638
9	11981627	0,638
9	11982077	0,638
9	11982424	0,638
9	11982630	0,638
9	11982640	0,638
9	11982766	0,638
9	11982836	0,638
9	11982877	0,638
9	11982888	0,638
9	11982985	0,638
9	11982987	0,638
9	11983025	0,638
9	11983179	0,638
9	11983198	0,638
9	11983223	0,638
9	11983229	0,638
9	11983256	0,638
9	11983289	0,638
9	11983290	0,638
9	11983338	0,638
9	11983432	0,638
9	11983497	0,638
9	11983574	0,638
9	11983749	0,638
9	11983811	0,638
9	11983824	0,638
9	11983889	0,638
9	11984081	0,638
9	11984307	0,638
9	11984480	0,638
9	11984482	0,638
9	11984579	0,638
9	11984923	0,638
9	11985072	0,638
9	11985495	0,638
9	11985526	0,638
9	11985583	0,638
9	11985658	0,638
9	11985714	0,638
9	11987252	0,638
9	11987281	0,638
9	11988367	0,638
9	11988455	0,638
9	11988619	0,638
9	11988633	0,638

9	11988790	0,638
9	11989297	0,638
9	11989330	0,638
9	11989709	0,638
9	11989759	0,638
9	11989798	0,638
9	11989832	0,638
9	11989884	0,638
9	11990136	0,638
9	11991299	0,638
9	11991323	0,638
9	11991331	0,638
9	11991393	0,638
9	11991409	0,638
9	11991627	0,638
9	11991861	0,638
9	11992966	0,638
9	11993542	0,638
9	11993589	0,638
9	11993855	0,638
9	11993961	0,638
9	11994001	0,638
9	11994071	0,638
9	11994105	0,638
9	11994275	0,638
9	11994336	0,638
9	11994422	0,638
9	11994430	0,638
9	11994900	0,638
9	11995437	0,638
9	11995711	0,638
9	11995931	0,638
9	11995935	0,638
9	11995952	0,638
9	11995956	0,638
9	11996042	0,638
9	11996055	0,638
9	11996439	0,638
9	11996872	0,638
9	11996884	0,638
9	11997061	0,638
9	11997159	0,638
9	11997174	0,638
9	11998738	0,638
9	11998799	0,638
9	12000109	0,638
9	12000180	0,638
9	12000201	0,638

9	12000390	0,638
9	12000421	0,638
9	12000429	0,638
9	12000452	0,638
9	12000453	0,638
9	12000462	0,638
9	12000471	0,638
9	12000517	0,638
9	12000534	0,638
9	12000550	0,638
9	12000774	0,638
9	12000785	0,638
9	12000823	0,638
9	12000881	0,638
9	12001048	0,638
9	12001233	0,638
9	12001364	0,638
9	12001440	0,638
9	12001724	0,638
9	12001732	0,638
9	12001821	0,638
9	12001824	0,638
9	12001915	0,638
9	12002797	0,638
9	12003205	0,638
9	12003207	0,638
9	12003271	0,638
9	12003371	0,638
9	12003444	0,638
9	12003478	0,638
9	12004074	0,638
9	12004718	0,638
9	12004720	0,638
9	12004732	0,638
9	12004819	0,638
9	12004824	0,638
9	12004883	0,638
9	12004889	0,638
9	12004909	0,638
9	12005407	0,638
9	12005408	0,638
9	12005626	0,638
9	12005629	0,638
9	12005731	0,638
9	12005751	0,638
9	12005866	0,638
9	12005872	0,638
9	12005956	0,638

9	12006844	0,638
9	12008822	0,638
9	12008895	0,638
9	12008957	0,638
9	12009018	0,638
9	12009173	0,638
9	12009180	0,638
9	12009244	0,638
9	12009301	0,638
9	12009398	0,638
9	12009612	0,638
9	12009666	0,638
9	12009805	0,638
9	12009820	0,638
9	12009826	0,638
9	12009827	0,638
9	12010004	0,638
9	12010609	0,638
9	12011146	0,638
9	12011211	0,638
9	12011510	0,638
9	12011573	0,638
9	12011816	0,638
9	12011950	0,638
9	12011964	0,638
9	12011965	0,638
9	12011980	0,638
9	12011991	0,638
9	12012022	0,638
9	12012900	0,638
9	12013133	0,638
9	12013286	0,638
9	12013288	0,638
9	12013290	0,638
9	12013334	0,638
9	12013407	0,638
9	12013415	0,638
9	12013416	0,638
9	12013432	0,638
9	12013487	0,638
9	12013565	0,638
9	12013711	0,638
9	12013953	0,638
9	12014394	0,638
9	12014558	0,638
9	12015016	0,638
9	12015193	0,638
9	12015224	0,638

9	12015259	0,638
9	12015360	0,638
9	12015529	0,638
9	12015948	0,638
9	12015997	0,638
9	12016445	0,638
9	12016597	0,638
9	12016735	0,638
9	12016778	0,638
9	12016808	0,638
9	12016872	0,638
9	12016918	0,638
9	12017502	0,638
9	12017546	0,638
9	12017638	0,638
9	12018616	0,638
9	12019653	0,638
9	12021445	0,638
9	12021450	0,638
9	12030014	0,638
9	12030048	0,638
9	12030093	0,638
9	12030327	0,638
9	12030609	0,638
9	12031886	0,638
9	12031921	0,638
9	12032019	0,638
9	12032618	0,638
9	12032982	0,638
9	12033370	0,638
9	12035705	0,638
9	12035820	0,638
9	12036056	0,638
9	12036252	0,638
9	12036257	0,638
9	12036620	0,638
9	12037016	0,638
9	12037191	0,638
9	12037237	0,638
9	12037817	0,638
9	12038127	0,638
9	12039688	0,638
9	12039702	0,638
9	12039739	0,638
9	12039815	0,638
9	12039991	0,638
9	12040206	0,638
9	12040243	0,638

9	12040364	0,638
9	12040517	0,638
9	12042182	0,638
9	12043019	0,638
9	12043059	0,638
9	12043391	0,638
9	12044233	0,638
9	12044603	0,638
9	12046555	0,638
9	12046651	0,638
9	12046788	0,638
9	12046830	0,638
9	12046910	0,638
9	12047278	0,638
9	12047528	0,638
9	12048076	0,638
9	12048577	0,638
9	12048581	0,638
9	12049238	0,638
9	12049407	0,638
9	12049485	0,638
9	12049571	0,638
9	12049582	0,638
9	12049688	0,638
9	12049689	0,638
9	12049896	0,638
9	12049996	0,638
9	12049997	0,638
9	12050057	0,638
9	12050103	0,638
9	12050128	0,638
9	12050141	0,638
9	12050144	0,638
9	12050189	0,638
9	12050216	0,638
9	12050446	0,638
9	12050556	0,638
9	12050580	0,638
9	12050618	0,638
9	12050650	0,638
9	12050690	0,638
9	12050701	0,638
9	12050714	0,638
9	12050776	0,638
9	12051200	0,638
9	12051801	0,638
9	12051870	0,638
9	12052018	0,638

9	12052150	0,638
9	12052271	0,638
9	12053194	0,638
9	12053282	0,638
9	12053486	0,638
9	12053511	0,638
9	12053514	0,638
9	12053515	0,638
9	12053522	0,638
9	12053713	0,638
9	12053721	0,638
9	12053733	0,638
9	12053886	0,638
9	12054255	0,638
9	12054404	0,638
9	12054724	0,638
9	12054786	0,638
9	12054922	0,638
9	12057052	0,638
9	12057327	0,638
9	12057377	0,638
9	12057411	0,638
9	12057466	0,638
9	12057595	0,638
9	12057990	0,638
9	12058145	0,638
9	12058289	0,638
9	12060542	0,638
9	12060708	0,638
9	12061212	0,638
9	12061777	0,638
9	12061827	0,638
9	12061956	0,638
9	12061972	0,638
9	12062064	0,638
9	12062094	0,638
9	12062185	0,638
9	12062199	0,638
9	12062286	0,638
9	12062468	0,638
9	12062873	0,638
9	12063058	0,638
9	12063331	0,638
9	12063520	0,638
9	12063847	0,638
9	12065371	0,638
9	12065962	0,638
9	12066129	0,638

9	12066609	0,638
9	12066695	0,638
9	12067059	0,638
9	12067168	0,638
9	12067532	0,638
9	12067553	0,638
9	12067835	0,638
9	12069509	0,638
9	12070556	0,638
9	12070677	0,638
9	12071514	0,638
9	12072538	0,638
9	12072818	0,638
9	12072840	0,638
9	12072972	0,638
9	12073500	0,638
9	12073513	0,638
9	12073530	0,638
9	12073798	0,638
9	12074167	0,638
9	12074880	0,638
9	12075379	0,638
9	12075429	0,638
9	12075540	0,638
9	12075740	0,638
9	12076073	0,638
9	12076696	0,638
9	12077316	0,638
9	12077364	0,638
9	12077714	0,638
9	12079001	0,638
9	12079021	0,638
9	12079090	0,638
9	12079201	0,638
9	12085583	0,638
9	12085674	0,759
9	12085728	0,759
9	12086314	0,638
9	12086672	0,759
9	12086919	0,759
9	12086965	0,759
9	12087045	0,759
9	12087231	0,638
9	12087273	0,759
9	12087690	0,638
9	12088911	0,759
9	12089117	0,638
9	12089121	0,759

9	12089332	0,638
9	12089526	0,638
9	12089717	0,638
9	12089855	0,638
9	12090572	0,638
9	12090669	0,638
9	12090685	0,638
9	12091299	0,759
9	12091814	0,759
9	12092516	0,638
9	12093027	0,638
9	12093081	0,638
9	12093235	0,759
9	12093236	0,759
9	12094451	0,638
9	12094678	0,759
9	12094885	0,759
9	12095035	0,638
9	12095156	0,759
9	12095255	0,638
9	12095436	0,759
9	12095652	0,759
9	12095700	0,759
9	12095703	0,759
9	12095733	0,759
9	12096897	0,759
9	12097993	0,759
9	12098028	0,759
9	12098262	0,759
9	12098272	0,759
9	12098351	0,759
9	12098583	0,759
9	12099345	0,759
9	12099660	0,759
9	12099682	0,759
9	12099847	0,759
9	12100166	0,759
9	12100414	0,638
9	12100951	0,638
9	12101146	0,638
9	12101463	0,759
9	12101470	0,638
9	12102232	0,638
9	12104797	0,638
9	12104821	0,759
9	12104969	0,759
9	12105184	0,638
9	12105413	0,759

9	12105490	0,638
9	12106003	0,759
9	12106480	0,759
9	12106548	0,638
9	12106573	0,638
9	12106655	0,638
9	12106659	0,638
9	12106666	0,759
9	12106795	0,638
9	12106803	0,638
9	12106949	0,759
9	12106974	0,759
9	12107232	0,759
9	12107651	0,638
9	12107881	0,759
9	12107965	0,638
9	12107974	0,759
9	12108003	0,638
9	12108272	0,638
9	12108366	0,638
9	12108376	0,638
9	12108459	0,638
9	12108466	0,638
9	12108478	0,759
9	12108488	0,638
9	12108518	0,638
9	12108675	0,759
9	12108712	0,638
9	12108940	0,759
9	12108978	0,759
9	12108998	0,759
9	12109012	0,759
9	12109017	0,759
9	12109032	0,759
9	12109041	0,638
9	12109439	0,759
9	12109538	0,638
9	12109651	0,638
9	12109801	0,638
9	12110719	0,759
9	12111825	0,638
9	12113020	0,638
9	12113052	0,638
9	12113418	0,638
9	12113491	0,638
9	12113593	0,638
9	12113662	0,638
9	12113790	0,638

9	12113804	0,638
9	12113971	0,638
9	12113975	0,638
9	12114037	0,638
9	12114153	0,638
9	12114218	0,638
9	12114410	0,638
9	12114535	0,638
9	12114538	0,638
9	12114585	0,638
9	12114630	0,638
9	12114672	0,638
9	12114678	0,638
9	12114724	0,638
9	12114902	0,638
9	12115622	0,638
9	12115779	0,638
9	12115861	0,638
9	12116196	0,759
9	12117031	0,759
9	12117057	0,759
9	12117247	0,638
9	12118800	0,759
9	12118837	0,759
9	12118871	0,759
9	12118873	0,759
9	12118888	0,759
9	12119092	0,759
9	12119363	0,759
9	12119831	0,759
9	12120260	0,759
9	12120301	0,759
9	12120358	0,759
9	12120365	0,759
9	12120377	0,759
9	12120385	0,759
9	12120392	0,759
9	12120393	0,759
9	12120418	0,759
9	12120456	0,759
9	12120537	0,759
9	12120581	0,759
9	12120601	0,759
9	12120787	0,759
9	12121147	0,759
9	12121148	0,759
9	12121525	0,759
9	12121710	0,759

9	12121972	0,759
9	12122338	0,759
9	12122537	0,759
9	12122802	0,759
9	12122907	0,759
9	12123536	0,759
9	12123738	0,759
9	12124431	0,759
9	12124489	0,638
9	12124546	0,638
9	12124661	0,638
9	12124785	0,759
9	12124888	0,638
9	12124932	0,638
9	12125002	0,759
9	12125026	0,759
9	12125437	0,638
9	12126590	0,638
9	12126845	0,759
9	12126935	0,759
9	12127285	0,759
9	12127388	0,638
9	12127726	0,759
9	12127799	0,638
9	12128436	0,759
9	12128665	0,759
9	12128726	0,638
9	12128954	0,638
9	12129845	0,638
9	12129963	0,638
9	12130126	0,638
9	12130934	0,759
9	12131134	0,759
9	12131285	0,759
9	12131748	0,759
9	12132368	0,638
9	12132378	0,638
9	12132394	0,759
9	12133442	0,638
9	12134018	0,638
9	12134295	0,759
9	12134550	0,759
9	12134987	0,759
9	12134989	0,759
9	12135113	0,759
9	12135134	0,638
9	12135174	0,638
9	12135263	0,638

9	12135565	0,638
9	12135581	0,638
9	12135870	0,759
9	12135980	0,638
9	12136514	0,759
9	12136583	0,638
9	12136620	0,759
9	12136798	0,759
9	12136838	0,759
9	12136856	0,638
9	12136903	0,759
9	12136922	0,759
9	12136985	0,759
9	12136990	0,759
9	12137009	0,759
9	12137118	0,759
9	12137199	0,638
9	12137226	0,638
9	12137230	0,638
9	12137235	0,638
9	12137742	0,759
9	12137860	0,759
9	12137867	0,638
9	12138029	0,638
9	12138202	0,638
9	12138239	0,638
9	12138460	0,638
9	12138463	0,638
9	12138478	0,638
9	12138494	0,638
9	12138575	0,638
9	12138591	0,638
9	12138679	0,759
9	12138720	0,759
9	12139381	0,759
9	12139484	0,759
9	12139640	0,759
9	12139734	0,759
9	12140590	0,759
9	12140760	0,759
9	12140868	0,638
9	12141534	0,759
9	12141705	0,638
9	12141845	0,759
9	12142088	0,759
9	12142135	0,759
9	12142246	0,759
9	12142298	0,759

9	12142422	0,759
9	12142689	0,759
9	12142705	0,759
9	12143192	0,759
9	12143243	0,759
9	12143440	0,759
9	12143464	0,759
9	12143667	0,759
9	12143808	0,759
9	12144159	0,759
9	12144160	0,759
9	12144462	0,759
9	12145273	0,759
9	12146669	0,759
9	12147025	0,759
9	12147276	0,759
9	12147555	0,759
9	12148278	0,759
9	12148484	0,759
9	12148576	0,759
9	12148887	0,759
9	12148964	0,759
9	12149108	0,759
9	12149675	0,759
9	12150044	0,638
9	12150257	0,638
9	12150568	0,759
9	12150853	0,759
9	12151164	0,759
9	12156022	0,638
9	12156042	0,638
9	12156097	0,638
9	12156116	0,638
9	12156167	0,638
9	12156287	0,638
9	12156320	0,638
9	12156322	0,638
9	12156323	0,638
9	12156354	0,638
9	12156498	0,638
9	12156563	0,638
9	12156662	0,638
9	12156797	0,638
9	12156929	0,638
9	12156935	0,638
9	12156973	0,638
9	12156991	0,759
9	12156992	0,638

9	12156999	0,638
9	12157007	0,638
9	12157024	0,638
9	12157170	0,638
9	12157209	0,638
9	12157225	0,638
9	12157249	0,638
9	12157317	0,638
9	12157445	0,638
9	12157605	0,638
9	12157860	0,638
9	12157898	0,638
9	12157928	0,759
9	12157934	0,759
9	12157963	0,759
9	12158136	0,759
9	12158175	0,759
9	12158194	0,759
9	12159563	0,638
9	12159694	0,759
9	12160053	0,638
9	12160590	0,638
9	12160634	0,759
9	12161120	0,759
9	12162155	0,638
9	12162747	0,638
9	12163120	0,638
9	12163263	0,759
9	12163521	0,638
9	12163894	0,759
9	12164193	0,759
9	12164624	0,759
9	12164638	0,759
9	12165451	0,759
9	12165718	0,638
9	12166340	0,638
9	12166607	0,638
9	12166618	0,759
9	12166805	0,638
9	12167358	0,759
9	12167359	0,638
9	12167518	0,638
9	12167533	0,638
9	12167557	0,759
9	12167606	0,759
9	12167631	0,638
9	12167632	0,638
9	12167745	0,638

9	12167980	0,759
9	12168180	0,638
9	12168190	0,759
9	12168237	0,759
9	12168296	0,759
9	12168380	0,759
9	12168382	0,759
9	12168494	0,759
9	12168500	0,759
9	12168503	0,759
9	12168512	0,759
9	12168513	0,759
9	12168623	0,759
9	12168657	0,638
9	12168661	0,759
9	12168671	0,759
9	12168703	0,759
9	12168715	0,759
9	12169469	0,638
9	12169502	0,638
9	12169605	0,638
9	12169884	0,759
9	12170030	0,638
9	12170064	0,638
9	12170068	0,638
9	12170421	0,759
9	12172173	0,759
9	12172186	0,638
9	12172500	0,759
9	12172583	0,638
9	12173093	0,638
9	12173974	0,638
9	12174075	0,759
9	12174283	0,759
9	12174616	0,638
9	12174625	0,759
9	12174931	0,638
9	12175436	0,638
9	12176138	0,759
9	12176764	0,638
9	12176942	0,759
9	12176967	0,759
9	12177786	0,638
9	12178487	0,759
9	12178646	0,638
9	12178706	0,638
9	12178866	0,638
9	12178929	0,638

9	12179093	0,638
9	12179264	0,638
9	12179347	0,638
9	12179426	0,638
9	12179635	0,759
9	12179885	0,759
9	12180375	0,759
9	12180387	0,759
9	12184251	0,759
9	12186872	0,759
9	12187024	0,638
9	12188144	0,759
9	12188506	0,759
9	12188996	0,638
9	12189227	0,638
9	12191638	0,638
9	12192954	0,759
9	12193026	0,759
9	12195491	0,759
9	12195492	0,638
9	12197053	0,759
9	12197154	0,638
9	12197635	0,759
9	12197828	0,759
9	12197927	0,638
9	12199029	0,759
9	12199033	0,638
9	12199596	0,638
9	12200150	0,638
9	12200460	0,759
9	12200464	0,759
9	12200508	0,759
9	12200825	0,759
9	12200861	0,759
9	12201476	0,759
9	12201488	0,759
9	12201510	0,759
9	12202917	0,759
9	12203099	0,638
9	12204183	0,638
9	12204984	0,638
9	12205403	0,759
9	12207043	0,759
9	12207978	0,638
9	12208417	0,638
9	12208434	0,638
9	12208536	0,638
9	12208558	0,638

9	12208742	0,638
9	12208749	0,638
9	12208763	0,638
9	12208848	0,638
9	12209259	0,638
9	12209414	0,638
9	12210188	0,638
9	12210721	0,638
9	12210727	0,638
9	12210826	0,638
9	12210907	0,759
9	12210952	0,759
9	12211013	0,759
9	12211357	0,759
9	12211466	0,638
9	12211510	0,638
9	12211708	0,638
9	12212939	0,638
9	12213179	0,759
9	12213417	0,759
9	12214704	0,638
9	12214816	0,638
9	12214852	0,638
9	12215436	0,759
9	12215465	0,759
9	12215473	0,759
9	12215513	0,759
9	12215519	0,759
9	12216244	0,759
9	12216256	0,759
9	12216723	0,759
9	12216741	0,759
9	12219468	0,638
9	12219469	0,638
9	12219474	0,759
9	12219703	0,638
9	12219730	0,759
9	12219734	0,638
9	12219742	0,759
9	12219746	0,759
9	12219787	0,759
9	12219797	0,638
9	12219812	0,638
9	12220036	0,638
9	12220161	0,638
9	12220235	0,759
9	12220468	0,759
9	12220495	0,638

9	12220544	0,638
9	12220572	0,638
9	12220582	0,759
9	12220683	0,638
9	12220701	0,759
9	12220913	0,759
9	12221108	0,638
9	12221247	0,638
9	12221257	0,638
9	12221347	0,638
9	12221537	0,638
9	12221554	0,759
9	12221565	0,759
9	12221569	0,638
9	12221719	0,759
9	12221800	0,638
9	12222244	0,638
9	12222660	0,759
9	12222685	0,638
9	12222757	0,759
9	12222895	0,638
9	12222949	0,638
9	12223559	0,638
9	12223797	0,638
9	12224329	0,759
9	12224619	0,638
9	12225008	0,759
9	12225184	0,759
9	12225188	0,759
9	12225392	0,759
9	12225695	0,638
9	12225703	0,638
9	12225709	0,638
9	12225747	0,638
9	12225784	0,638
9	12225830	0,638
9	12225894	0,638
9	12225911	0,638
9	12225942	0,638
9	12226045	0,638
9	12226078	0,638
9	12226225	0,638
9	12226227	0,638
9	12227369	0,759
9	12228307	0,638
9	12228940	0,638
9	12228979	0,638
9	12229081	0,638

9	12229492	0,638
9	12229922	0,638
9	12230910	0,638
9	12231450	0,638
9	12231718	0,638
9	12231719	0,638
9	12231769	0,638
9	12231793	0,638
9	12231870	0,638
9	12232031	0,638
9	12233111	0,759
9	12234070	0,638
9	12234700	0,759
9	12234826	0,759
9	12235232	0,759
9	12235290	0,759
9	12235297	0,759
9	12235350	0,759
9	12235396	0,759
9	12235647	0,759
9	12235690	0,638
9	12235698	0,759
9	12235748	0,759
9	12235749	0,759
9	12235803	0,759
9	12235886	0,759
9	12235924	0,759
9	12235964	0,759
9	12235965	0,759
9	12235998	0,759
9	12236023	0,759
9	12236029	0,759
9	12236058	0,759
9	12236060	0,759
9	12236065	0,759
9	12236124	0,759
9	12236138	0,759
9	12236151	0,759
9	12236158	0,759
9	12236173	0,759
9	12236193	0,759
9	12236197	0,759
9	12236218	0,759
9	12236220	0,759
9	12236223	0,759
9	12236338	0,759
9	12236345	0,759
9	12236399	0,759

9	12236593	0,759
9	12236597	0,759
9	12236663	0,759
9	12236664	0,759
9	12236806	0,759
9	12236938	0,759
9	12236971	0,759
9	12237079	0,638
9	12237117	0,759
9	12238036	0,638
9	12238194	0,638
9	12238302	0,638
9	12238544	0,759
9	12238551	0,759
9	12238590	0,759
9	12238655	0,759
9	12238735	0,759
9	12238814	0,759
9	12239015	0,638
9	12239049	0,759
9	12239186	0,759
9	12239238	0,759
9	12239267	0,759
9	12239539	0,638
9	12239642	0,759
9	12239825	0,638
9	12239828	0,638
9	12240063	0,638
9	12240212	0,638
9	12240496	0,638
9	12240980	0,759
9	12241094	0,759
9	12241095	0,759
9	12241629	0,759
9	12241772	0,759
9	12241811	0,638
9	12242308	0,638
9	12242438	0,759
9	12242498	0,759
9	12242802	0,759
9	12243041	0,759
9	12243639	0,638
9	12243645	0,638
9	12243701	0,759
9	12243747	0,759
9	12243794	0,759
9	12243800	0,759
9	12244474	0,638

9	12244564	0,638
9	12244692	0,759
9	12244866	0,759
9	12244969	0,759
9	12244977	0,759
9	12245651	0,759
9	12245685	0,759
9	12245764	0,759
9	12245771	0,759
9	12245831	0,759
9	12246535	0,759
9	12246629	0,759
9	12246651	0,759
9	12246724	0,759
9	12246952	0,759
9	12247026	0,759
9	12247028	0,759
9	12247125	0,759
9	12247168	0,759
9	12247186	0,759
9	12247257	0,759
9	12247272	0,759
9	12247347	0,759
9	12247969	0,759
9	12247982	0,759
9	12248069	0,759
9	12248140	0,759
9	12248195	0,759
9	12248396	0,759
9	12248410	0,759
9	12248471	0,759
9	12248546	0,759
9	12248965	0,759
9	12249132	0,759
9	12249166	0,759
9	12249172	0,759
9	12249262	0,759
9	12249328	0,759
9	12249559	0,759
9	12249726	0,759
9	12249727	0,759
9	12249756	0,759
9	12249778	0,759
9	12249791	0,759
9	12249858	0,759
9	12249929	0,759
9	12249981	0,759
9	12250081	0,759

9	12250115	0,759
9	12250187	0,759
9	12250423	0,759
9	12250466	0,759
9	12251186	0,759
9	12251213	0,759
9	12251469	0,759
9	12251760	0,759
9	12252021	0,759
9	12252057	0,759
9	12252149	0,759
9	12252673	0,759
9	12252789	0,759
9	12252969	0,759
9	12253200	0,759
9	12253255	0,759
9	12253286	0,759
9	12253290	0,759
9	12253296	0,759
9	12253368	0,759
9	12253393	0,759
9	12253421	0,759
9	12254129	0,638
9	12257293	0,759
9	12257358	0,759
9	12257531	0,759
9	12257537	0,759
9	12257557	0,759
9	12257577	0,759
9	12257600	0,759
9	12257673	0,759
9	12257739	0,759
9	12258152	0,759
9	12258260	0,759
9	12258316	0,759
9	12258403	0,638
9	12258413	0,638
9	12258428	0,638
9	12258440	0,638
9	12258441	0,638
9	12258608	0,638
9	12258685	0,759
9	12258692	0,759
9	12258703	0,759
9	12258705	0,759
9	12258790	0,638
9	12258817	0,638
9	12258970	0,638

9	12259186	0,759
9	12259187	0,759
9	12259591	0,759
9	12259782	0,759
9	12259906	0,759
9	12260158	0,759
9	12260517	0,759
9	12260563	0,759
9	12260837	0,759
9	12261063	0,759
9	12261587	0,759
9	12261658	0,638
9	12262298	0,638
9	12262952	0,638
9	12263708	0,759
9	12263717	0,638
9	12263779	0,759
9	12264125	0,759
9	12264149	0,759
9	12264696	0,638
9	12264862	0,638
9	12265029	0,759
9	12265049	0,759
9	12265168	0,759
9	12265208	0,638
9	12265219	0,638
9	12265226	0,638
9	12265342	0,638
9	12265367	0,638
9	12266365	0,759
9	12267555	0,638
9	12267795	0,759
9	12267980	0,638
9	12268083	0,638
9	12268243	0,759
9	12269098	0,759
9	12269192	0,759
9	12269261	0,759
9	12269297	0,759
9	12270004	0,759
9	12270231	0,759
9	12270340	0,759
9	12270342	0,759
9	12270357	0,759
9	12270369	0,759
9	12270439	0,759
9	12270608	0,759
9	12270687	0,759

9	12271085	0,759
9	12271151	0,638
9	12271493	0,759
9	12271657	0,759
9	12271661	0,759
9	12271701	0,759
9	12272585	0,759
9	12272608	0,759
9	12272633	0,638
9	12272786	0,638
9	12272846	0,759
9	12273111	0,759
9	12273121	0,759
9	12273135	0,759
9	12273159	0,759
9	12273317	0,638
9	12273657	0,759
9	12273718	0,759
9	12273847	0,759
9	12274147	0,638
9	12274157	0,759
9	12274191	0,759
9	12274224	0,759
9	12274356	0,759
9	12274361	0,759
9	12274483	0,759
9	12274542	0,638
9	12274616	0,759
9	12274624	0,759
9	12274679	0,759
9	12274690	0,638
9	12274735	0,759
9	12275055	0,638
9	12275299	0,759
9	12275388	0,638
9	12275402	0,638
9	12275420	0,638
9	12275562	0,638
9	12275574	0,638
9	12275697	0,759
9	12275728	0,638
9	12275825	0,638
9	12276122	0,759
9	12276177	0,638
9	12276223	0,759
9	12276241	0,638
9	12276314	0,759
9	12276339	0,638

9	12276572	0,759
9	12276686	0,759
9	12276790	0,638
9	12277069	0,638
9	12277529	0,759
9	12277844	0,638
9	12277912	0,638
9	12278018	0,638
9	12278058	0,759
9	12278365	0,638
9	12278783	0,638
9	12278887	0,638
9	12278897	0,638
9	12278898	0,759
9	12279722	0,759
9	12280317	0,759
9	12280416	0,759
9	12280524	0,759
9	12280564	0,759
9	12280576	0,759
9	12280598	0,759
9	12280600	0,759
9	12280635	0,759
9	12280703	0,759
9	12280832	0,759
9	12280874	0,759
9	12281097	0,759
9	12281107	0,759
9	12281250	0,759
9	12281272	0,759
9	12281297	0,759
9	12281817	0,759
9	12281818	0,759
9	12282066	0,759
9	12282110	0,759
9	12282135	0,759
9	12282192	0,759
9	12282398	0,759
9	12282418	0,759
9	12282476	0,759
9	12282554	0,759
9	12282640	0,759
9	12282660	0,759
9	12282661	0,759
9	12282847	0,759
9	12282885	0,759
9	12282945	0,759
9	12282948	0,759

9	12282964	0,759
9	12283071	0,759
9	12283074	0,759
9	12283146	0,759
9	12283226	0,759
9	12283636	0,759
9	12283720	0,759
9	12283916	0,759
9	12284194	0,759
9	12284419	0,759
9	12285318	0,759
9	12286390	0,759
9	12286697	0,759
9	12286716	0,759
9	12286730	0,759
9	12286847	0,759
9	12286970	0,759
9	12287222	0,759
9	12287251	0,759
9	12287272	0,759
9	12287273	0,759
9	12287274	0,759
9	12287281	0,759
9	12287306	0,759
9	12287319	0,759
9	12287324	0,759
9	12287330	0,759
9	12287337	0,759
9	12287406	0,759
9	12287494	0,759
9	12287575	0,759
9	12287600	0,759
9	12287696	0,759
9	12288445	0,759
9	12288451	0,759
9	12288544	0,759
9	12288550	0,759
9	12288789	0,759
9	12288820	0,759
9	12289000	0,759
9	12289030	0,759
9	12289048	0,759
9	12289049	0,759
9	12289058	0,759
9	12289061	0,759
9	12289135	0,759
9	12289139	0,759
9	12289167	0,759

9	12289168	0,759
9	12289191	0,759
9	12289246	0,759
9	12289254	0,759
9	12289453	0,759
9	12289495	0,759
9	12289535	0,759
9	12289539	0,759
9	12289559	0,759
9	12289592	0,759
9	12289903	0,759
9	12289982	0,759
9	12290238	0,759
9	12290261	0,759
9	12290291	0,759
9	12290353	0,759
9	12300860	0,759
9	12301375	0,759
9	12301528	0,759
9	12301836	0,759
9	12301865	0,638
9	12301897	0,638
9	12301947	0,759
9	12301966	0,759
9	12302017	0,759
9	12302023	0,759
9	12302024	0,759
9	12302053	0,759
9	12302057	0,759
9	12302084	0,759
9	12302121	0,759
9	12302125	0,759
9	12302196	0,759
9	12302306	0,759
9	12302351	0,759
9	12302394	0,759
9	12302406	0,638
9	12302585	0,759
9	12302586	0,759
9	12302593	0,759
9	12302681	0,759
9	12302685	0,759
9	12302688	0,759
9	12302689	0,759
9	12302810	0,759
9	12302888	0,759
9	12302892	0,759
9	12304024	0,759

9	12304050	0,759
9	12304319	0,638
9	12304406	0,638
9	12305007	0,759
9	12305104	0,759
9	12305357	0,759
9	12305717	0,759
9	12306813	0,638
9	12306938	0,638
9	12306971	0,638
9	12306996	0,759
9	12308088	0,759
9	12317698	0,638
9	12322285	0,759
9	12328152	0,759
9	12347483	0,759
9	12392204	0,638
9	12418481	0,638
9	12419009	0,638
9	12419914	0,638
9	12420170	0,638
9	12420180	0,638
9	12420448	0,638
9	12420497	0,638
9	12421139	0,638
9	12421179	0,638
9	12421324	0,638
9	12421589	0,638
9	12421751	0,638
9	12422008	0,638
9	12422252	0,638
9	12422260	0,638
9	12422269	0,638
9	12422316	0,638
9	12422350	0,638
9	12422465	0,638
9	12422606	0,638
9	12423076	0,638
9	12423477	0,638
9	12423657	0,638
9	12423727	0,638
9	12424327	0,638
9	12424513	0,638
9	12424568	0,638
9	12424625	0,638
9	12424756	0,638
9	12424934	0,638
9	12424971	0,638

9	12425179	0,638
9	12426015	0,638
9	12426147	0,638
9	12426209	0,638
9	12426238	0,638
9	12426387	0,638
9	12426408	0,638
9	12426547	0,638
9	12426573	0,638
9	12426693	0,638
9	12426872	0,638
9	12426893	0,638
9	12427240	0,638
9	12427336	0,638
9	12427369	0,638
9	12427591	0,638
9	12427733	0,638
9	12427802	0,638
9	12427813	0,638
9	12427899	0,638
9	12427930	0,638
9	12428223	0,638
9	12428400	0,638
9	12428402	0,638
9	12428486	0,638
9	12429297	0,638
9	12429369	0,638
9	12429673	0,638
9	12429725	0,638
9	12429745	0,638
9	12429871	0,638
9	12429985	0,638
9	12430354	0,638
9	12430417	0,638
9	12430850	0,759
9	12434820	0,638
9	12434876	0,638
9	12435163	0,759
9	12435654	0,638
9	12435681	0,638
9	12435693	0,638
9	12435802	0,638
9	12435809	0,638
9	12435977	0,638
9	12435995	0,638
9	12436022	0,638
9	12436031	0,638
9	12436188	0,638

9	12436246	0,638
9	12436477	0,638
9	12437148	0,638
9	12437182	0,638
9	12437205	0,638
9	12437329	0,638
9	12437757	0,638
9	12437810	0,638
9	12437812	0,638
9	12437831	0,638
9	12437834	0,638
9	12437985	0,638
9	12438002	0,638
9	12438186	0,638
9	12438252	0,638
9	12438356	0,638
9	12438554	0,638
9	12438562	0,638
9	12439236	0,638
9	12440012	0,638
9	12440054	0,638
9	12440094	0,638
9	12440100	0,638
9	12440134	0,638
9	12440206	0,638
9	12440225	0,638
9	12440264	0,638
9	12440297	0,638
9	12440342	0,638
9	12440511	0,638
9	12440650	0,638
9	12440692	0,638
9	12440722	0,638
9	12440751	0,638
9	12440819	0,638
9	12440846	0,638
9	12440914	0,638
9	12440922	0,638
9	12441914	0,638
9	12442029	0,638
9	12442074	0,638
9	12442262	0,638
9	12442435	0,638
9	12442879	0,638
9	12442912	0,638
9	12442949	0,638
9	12443156	0,638
9	12443173	0,638

9	12443235	0,638
9	12443254	0,638
9	12443265	0,638
9	12443363	0,638
9	12443608	0,638
9	12443631	0,638
9	12444214	0,638
9	12444280	0,638
9	12444337	0,638
9	12444490	0,638
9	12444578	0,638
9	12445145	0,638
9	12445353	0,638
9	12445369	0,638
9	12445376	0,638
9	12445474	0,638
9	12446007	0,638
9	12446122	0,638
9	12446171	0,638
9	12446246	0,638
9	12446314	0,638
9	12446320	0,638
9	12446369	0,638
9	12446516	0,638
9	12446530	0,638
9	12446868	0,638
9	12446908	0,638
9	12447039	0,638
9	12447061	0,638
9	12447122	0,638
9	12447198	0,638
9	12447242	0,638
9	12447243	0,638
9	12447254	0,638
9	12447339	0,638
9	12447724	0,638
9	12447730	0,638
9	12447748	0,638
9	12447764	0,638
9	12447792	0,638
9	12447836	0,638
9	12447854	0,638
9	12448209	0,638
9	12448244	0,638
9	12448838	0,638
9	12448866	0,638
9	12448867	0,638
9	12448871	0,638

9	12448924	0,638
9	12449044	0,638
9	12449056	0,638
9	12449062	0,638
9	12449071	0,638
9	12449079	0,638
9	12449317	0,638
9	12449386	0,638
9	12449573	0,638
9	12449770	0,638
9	12449775	0,638
9	12450295	0,638
9	12450497	0,638
9	12450838	0,638
9	12451131	0,638
9	12451252	0,638
9	12451328	0,638
9	12451347	0,638
9	12451370	0,638
9	12451426	0,638
9	12451436	0,638
9	12451440	0,638
9	12451582	0,638
9	12451593	0,638
9	12451603	0,638
9	12451608	0,638
9	12451996	0,638
9	12452255	0,638
9	12452760	0,638
9	12452926	0,638
9	12453482	0,638
9	12453531	0,638
9	12454064	0,638
9	12454065	0,638
9	12455249	0,638
9	12455674	0,638
9	12455970	0,638
9	12456009	0,638
9	12456150	0,638
9	12456541	0,638
9	12457425	0,759
9	12457703	0,638
9	12458122	0,759
9	12458575	0,638
9	12458602	0,638
9	12458604	0,638
9	12458669	0,638
9	12459349	0,638

9	12459546	0,759
9	12459625	0,638
9	12459647	0,638
9	12459829	0,759
9	12460723	0,638
9	12461019	0,759
9	12461117	0,759
9	12461118	0,759
9	12461218	0,759
9	12461332	0,638
9	12461733	0,638
9	12461857	0,759
9	12461873	0,759
9	12461888	0,759
9	12462232	0,759
9	12462627	0,759
9	12462973	0,759
9	12462991	0,638
9	12462992	0,638
9	12462993	0,638
9	12463132	0,759
9	12463978	0,638
9	12463994	0,759
9	12464251	0,759
9	12464254	0,759
9	12464258	0,638
9	12464319	0,759
9	12464336	0,759
9	12464562	0,759
9	12464582	0,759
9	12465272	0,759
9	12465439	0,759
9	12465687	0,759
9	12466018	0,759
9	12466063	0,759
9	12466389	0,759
9	12467147	0,759
9	12467304	0,638
9	12467885	0,638
9	12468099	0,759
9	12468182	0,638
9	12468529	0,759
9	12468594	0,759
9	12468666	0,759
9	12469040	0,759
9	12469133	0,759
9	12469134	0,759
9	12469196	0,638

9	12469315	0,638
9	12469757	0,759
9	12470271	0,759
9	12471037	0,759
9	12471061	0,759
9	12471252	0,759
9	12471339	0,638
9	12471585	0,759
9	12471593	0,759
9	12471627	0,638
9	12471674	0,759
9	12472025	0,759
9	12472032	0,638
9	12472065	0,759
9	12472408	0,759
9	12472901	0,638
9	12472906	0,638
9	12472908	0,638
9	12472917	0,759
9	12472978	0,638
9	12473104	0,638
9	12473120	0,638
9	12473152	0,638
9	12473188	0,759
9	12473234	0,759
9	12473245	0,638
9	12473304	0,759
9	12473316	0,759
9	12473366	0,759
9	12473371	0,638
9	12473407	0,759
9	12473423	0,759
9	12473790	0,759
9	12473805	0,759
9	12473806	0,759
9	12473862	0,759
9	12473887	0,759
9	12473964	0,638
9	12474312	0,638
9	12474368	0,638
9	12474586	0,759
9	12474594	0,759
9	12474633	0,759
9	12474897	0,638
9	12475077	0,759
9	12475085	0,638
9	12475270	0,638
9	12475306	0,759

9	12475328	0,638
9	12475395	0,759
9	12475973	0,759
9	12475994	0,759
9	12476047	0,759
9	12476196	0,759
9	12476216	0,759
9	12476287	0,638
9	12476370	0,638
9	12476375	0,638
9	12476482	0,759
9	12476539	0,759
9	12476717	0,638
9	12476772	0,759
9	12476874	0,638
9	12477373	0,638
9	12477405	0,638
9	12477442	0,638
9	12477504	0,638
9	12477515	0,638
9	12477533	0,638
9	12477538	0,638
9	12477644	0,638
9	12477675	0,638
9	12477679	0,638
9	12477682	0,638
9	12477783	0,759
9	12477797	0,759
9	12477828	0,638
9	12477863	0,638
9	12477988	0,638
9	12477997	0,759
9	12478000	0,759
9	12478004	0,638
9	12478158	0,638
9	12478163	0,638
9	12478177	0,638
9	12478523	0,759
9	12478539	0,759
9	12478551	0,759
9	12478553	0,759
9	12478570	0,638
9	12478678	0,759
9	12478892	0,638
9	12478964	0,759
9	12479044	0,638
9	12479054	0,759
9	12479143	0,759

9	12479591	0,638
9	12479593	0,638
9	12479595	0,638
9	12479598	0,638
9	12479945	0,638
9	12479973	0,759
9	12480110	0,759
9	12480337	0,638
9	12480344	0,638
9	12480400	0,638
9	12480405	0,638
9	12480451	0,759
9	12480566	0,638
9	12480575	0,759
9	12480779	0,759
9	12480902	0,638
9	12480944	0,638
9	12481108	0,638
9	12481121	0,638
9	12481269	0,638
9	12481300	0,759
9	12481370	0,638
9	12481430	0,759
9	12481442	0,759
9	12481443	0,759
9	12481448	0,638
9	12481559	0,638
9	12481562	0,638
9	12481573	0,759
9	12481595	0,759
9	12481597	0,759
9	12481617	0,759
9	12481618	0,759
9	12481621	0,759
9	12481646	0,759
9	12481662	0,638
9	12481684	0,759
9	12481712	0,759
9	12481737	0,759
9	12481926	0,638
9	12481927	0,638
9	12482185	0,638
9	12482462	0,759
9	12482464	0,759
9	12482499	0,638
9	12482633	0,638
9	12482747	0,638
9	12482810	0,759

9	12482835	0,759
9	12482841	0,759
9	12483009	0,759
9	12483128	0,638
9	12483207	0,759
9	12483628	0,759
9	12483973	0,759
9	12484246	0,759
9	12484611	0,638
9	12484654	0,759
9	12484890	0,638
9	12484986	0,638
9	12484989	0,638
9	12485021	0,638
9	12485094	0,759
9	12485317	0,638
9	12485331	0,759
9	12485459	0,638
9	12485471	0,638
9	12485494	0,638
9	12485536	0,759
9	12485544	0,759
9	12485702	0,759
9	12486146	0,638
9	12486191	0,759
9	12486231	0,638
9	12486486	0,638
9	12486725	0,638
9	12487021	0,759
9	12487076	0,759
9	12487642	0,638
9	12487643	0,638
9	12487850	0,638
9	12487874	0,638
9	12487918	0,759
9	12487931	0,638
9	12487964	0,638
9	12488229	0,759
9	12488380	0,638
9	12488427	0,759
9	12488443	0,759
9	12488444	0,759
9	12488452	0,759
9	12488471	0,759
9	12488647	0,759
9	12488694	0,638
9	12488834	0,759
9	12488937	0,759

9	12489003	0,638
9	12489102	0,638
9	12489123	0,638
9	12489476	0,638
9	12489516	0,638
9	12489519	0,759
9	12490430	0,638
9	12490557	0,759
9	12490920	0,759
9	12490946	0,638
9	12491076	0,638
9	12491328	0,638
9	12491368	0,638
9	12491423	0,638
9	12491762	0,759
9	12491870	0,759
9	12492332	0,638
9	12492339	0,759
9	12492418	0,638
9	12492625	0,638
9	12492730	0,759
9	12492856	0,759
9	12493644	0,759
9	12494261	0,759
9	12494344	0,638
9	12494542	0,759
9	12494963	0,759
9	12495071	0,759
9	12495108	0,759
9	12495243	0,638
9	12495279	0,759
9	12495366	0,638
9	12495407	0,759
9	12495408	0,759
9	12495421	0,759
9	12495473	0,759
9	12495474	0,759
9	12495796	0,759
9	12495874	0,638
9	12495916	0,759
9	12495986	0,759
9	12496000	0,759
9	12496076	0,638
9	12496316	0,759
9	12496433	0,638
9	12496494	0,759
9	12496656	0,638
9	12496732	0,638

9	12496801	0,638
9	12496875	0,759
9	12496906	0,759
9	12496924	0,638
9	12497089	0,759
9	12497115	0,759
9	12497356	0,638
9	12497436	0,638
9	12497584	0,759
9	12497591	0,759
9	12497706	0,638
9	12497829	0,759
9	12497889	0,759
9	12498192	0,638
9	12498253	0,759
9	12498286	0,638
9	12498369	0,638
9	12498449	0,638
9	12498508	0,638
9	12498527	0,638
9	12498669	0,638
9	12499124	0,759
9	12499145	0,759
9	12499152	0,759
9	12499184	0,759
9	12499200	0,759
9	12499287	0,759
9	12499330	0,759
9	12499351	0,759
9	12499356	0,759
9	12499853	0,759
9	12499865	0,759
9	12500076	0,759
9	12500082	0,759
9	12500127	0,638
9	12500192	0,759
9	12500304	0,759
9	12500394	0,759
9	12500419	0,638
9	12500446	0,638
9	12500506	0,638
9	12500518	0,638
9	12500582	0,638
9	12500589	0,638
9	12501163	0,759
9	12501500	0,638
9	12501520	0,638
9	12501531	0,638

9	12501699	0,638
9	12501796	0,638
9	12501802	0,638
9	12501832	0,638
9	12502218	0,638
9	12502572	0,638
9	12502607	0,759
9	12503804	0,638
9	12503850	0,759
9	12504367	0,759
9	12504502	0,638
9	12505294	0,759
9	12505339	0,759
9	12505663	0,638
9	12505675	0,638
9	12505701	0,759
9	12505752	0,759
9	12505791	0,759
9	12505812	0,638
9	12505831	0,759
9	12505939	0,638
9	12506040	0,638
9	12506045	0,638
9	12506270	0,759
9	12506748	0,638
9	12506804	0,638
9	12506828	0,638
9	12506829	0,638
9	12506994	0,759
9	12507048	0,759
9	12507066	0,759
9	12507277	0,638
9	12507466	0,638
9	12507660	0,638
9	12507865	0,638
9	12507972	0,638
9	12507998	0,638
9	12507999	0,759
9	12509869	0,638
9	12510361	0,638
9	12512305	0,759
9	12513015	0,638
9	12513172	0,638
9	12513175	0,638
9	12513217	0,638
9	12513376	0,759
9	12513379	0,759
9	12513386	0,638

9	12513396	0,638
9	12513411	0,638
9	12513524	0,638
9	12513526	0,638
9	12513746	0,638
9	12513903	0,759
9	12513904	0,759
9	12514181	0,759
9	12514204	0,759
9	12514210	0,759
9	12514211	0,759
9	12514244	0,638
9	12514374	0,759
9	12514406	0,759
9	12514474	0,638
9	12514496	0,759
9	12514497	0,759
9	12514518	0,759
9	12514760	0,759
9	12514813	0,638
9	12515105	0,638
9	12515259	0,638
9	12515344	0,759
9	12516556	0,759
9	12516800	0,759
9	12516906	0,759
9	12517442	0,638
9	12517545	0,638
9	12517764	0,638
9	12517926	0,638
9	12518013	0,638
9	12518143	0,638
9	12518213	0,638
9	12518219	0,638
9	12518401	0,759
9	12518554	0,759
9	12519003	0,638
9	12519010	0,759
9	12519026	0,759
9	12519713	0,638
9	12519728	0,638
9	12519783	0,759
9	12519854	0,759
9	12519877	0,759
9	12519902	0,759
9	12519929	0,638
9	12520233	0,638
9	12521244	0,638

9	12521363	0,638
9	12521411	0,638
9	12521457	0,638
9	12522050	0,638
9	12522090	0,638
9	12522667	0,759
9	12522729	0,759
9	12522803	0,759
9	12522882	0,759
9	12522892	0,638
9	12523409	0,638
9	12523832	0,638
9	12523893	0,638
9	12524094	0,638
9	12524331	0,638
9	12524617	0,638
9	12525364	0,638
9	12525852	0,638
9	12525943	0,638
9	12526275	0,638
9	12526398	0,638
9	12526404	0,638
9	12526470	0,638
9	12526509	0,638
9	12526831	0,638
9	12527081	0,638
9	12527090	0,638
9	12527167	0,638
9	12527171	0,638
9	12527173	0,638
9	12527175	0,638
9	12527184	0,638
9	12527214	0,638
9	12527256	0,638
9	12527296	0,638
9	12527459	0,638
9	12527500	0,638
9	12527519	0,638
9	12527522	0,638
9	12527583	0,638
9	12527616	0,638
9	12527728	0,638
9	12527923	0,638
9	12528251	0,638
9	12528501	0,638
9	12528512	0,638
9	12528578	0,638
9	12528579	0,638

9	12528751	0,638
9	12528765	0,759
9	12528900	0,638
9	12529110	0,759
9	12529141	0,759
9	12529474	0,638
9	12529525	0,759
9	12529608	0,759
9	12529652	0,759
9	12529758	0,638
9	12529769	0,759
9	12529807	0,759
9	12529844	0,638
9	12529852	0,638
9	12530376	0,638
9	12530394	0,638
9	12530709	0,638
9	12530758	0,638
9	12530765	0,638
9	12530913	0,759
9	12531251	0,759
9	12531271	0,759
9	12531277	0,759
9	12531423	0,759
9	12531573	0,638
9	12531632	0,638
9	12531764	0,759
9	12532987	0,638
9	12533011	0,759
9	12533623	0,638
9	12533823	0,759
9	12534398	0,759
9	12534648	0,759
9	12534802	0,638
9	12535617	0,759
9	12535677	0,759
9	12535827	0,638
9	12536093	0,638
9	12536649	0,638
9	12536723	0,759
9	12536743	0,759
9	12536748	0,759
9	12536772	0,759
9	12536805	0,759
9	12536870	0,638
9	12536871	0,759
9	12536881	0,759
9	12536882	0,759

9	12536931	0,759
9	12537115	0,638
9	12537125	0,759
9	12537128	0,759
9	12537135	0,638
9	12537323	0,759
9	12537865	0,759
9	12537915	0,759
9	12538044	0,638
9	12538062	0,759
9	12538705	0,638
9	12538881	0,638
9	12538882	0,638
9	12539065	0,638
9	12539137	0,638
9	12539156	0,638
9	12539440	0,638
9	12539459	0,638
9	12539482	0,638
9	12539916	0,638
9	12540079	0,759
9	12540100	0,638
9	12540136	0,638
9	12540297	0,638
9	12540363	0,638
9	12540450	0,638
9	12540456	0,638
9	12540522	0,638
9	12540562	0,638
9	12540697	0,638
9	12540983	0,759
9	12540994	0,638
9	12541058	0,638
9	12541141	0,638
9	12541142	0,638
9	12541370	0,638
9	12541386	0,638
9	12541553	0,638
9	12541554	0,638
9	12541566	0,638
9	12541590	0,638
9	12541606	0,638
9	12541640	0,638
9	12542082	0,638
9	12542182	0,638
9	12542238	0,638
9	12542478	0,638
9	12542495	0,638

9	12542535	0,638
9	12542540	0,638
9	12542590	0,638
9	12542592	0,638
9	12543081	0,638
9	12543481	0,638
9	12543484	0,638
9	12543499	0,759
9	12543549	0,759
9	12543622	0,638
9	12543638	0,638
9	12543683	0,638
9	12543818	0,638
9	12543836	0,638
9	12543852	0,638
9	12543957	0,638
9	12544142	0,638
9	12544207	0,638
9	12544233	0,638
9	12544263	0,638
9	12544268	0,638
9	12544299	0,638
9	12544357	0,638
9	12544368	0,638
9	12544415	0,638
9	12544433	0,759
9	12544447	0,638
9	12544478	0,638
9	12544533	0,638
9	12544686	0,638
9	12544695	0,638
9	12544715	0,638
9	12544757	0,638
9	12544802	0,638
9	12544827	0,638
9	12544846	0,638
9	12545415	0,638
9	12545424	0,638
9	12545529	0,638
9	12545674	0,638
9	12545692	0,638
9	12545745	0,638
9	12545750	0,638
9	12545758	0,638
9	12545765	0,638
9	12545780	0,638
9	12545822	0,638
9	12545840	0,638

9	12545863	0,638
9	12546031	0,638
9	12547733	0,638
9	12548598	0,759
9	12549079	0,638
9	12549363	0,638
9	12549713	0,638
9	12549987	0,638
9	12550147	0,638
9	12550540	0,759
9	12550560	0,759
9	12550580	0,638
9	12550608	0,638
9	12550653	0,638
9	12550804	0,759
9	12551397	0,759
9	12551658	0,638
9	12551733	0,759
9	12551753	0,638
9	12551762	0,638
9	12552463	0,638
9	12552870	0,759
9	12552993	0,759
9	12553588	0,759
9	12553953	0,759
9	12554132	0,759
9	12554370	0,759
9	12554504	0,759
9	12554778	0,638
9	12555020	0,759
9	12555359	0,759
9	12555428	0,759
9	12555536	0,759
9	12555619	0,638
9	12555920	0,759
9	12556209	0,759
9	12556390	0,759
9	12556507	0,638
9	12556598	0,638
9	12556640	0,638
9	12556793	0,638
9	12556824	0,638
9	12556995	0,759
9	12557280	0,638
9	12557321	0,638
9	12557397	0,638
9	12557651	0,638
9	12557656	0,638

9	12557679	0,638
9	12557710	0,759
9	12557813	0,638
9	12557876	0,638
9	12557899	0,638
9	12557914	0,638
9	12557927	0,638
9	12558224	0,638
9	12558251	0,638
9	12558307	0,759
9	12558530	0,759
9	12558653	0,638
9	12558721	0,638
9	12558813	0,638
9	12558920	0,638
9	12558952	0,759
9	12559180	0,759
9	12559377	0,759
9	12559776	0,759
9	12559909	0,759
9	12560056	0,759
9	12560165	0,759
9	12560365	0,759
9	12560371	0,759
9	12560490	0,759
9	12560733	0,759
9	12561617	0,638
9	12561778	0,759
9	12561876	0,759
9	12561996	0,759
9	12562004	0,759
9	12562295	0,638
9	13351844	0,500
9	13368083	0,638
9	13392285	0,586
9	13392310	0,586
9	13392419	0,586
9	13392478	0,586
9	13392481	0,586
9	13392498	0,586
9	13392553	0,586
9	13392735	0,586
9	13392736	0,586
9	13392772	0,586
9	13392776	0,586
9	13393056	0,586
9	13393438	0,586
9	13393535	0,586

9	13393585	0,586
9	13394128	0,586
9	13402514	0,586
9	13406320	0,534
9	13406415	0,534
9	13406444	0,534
9	13406487	0,534
9	13406495	0,534
9	13406533	0,534
9	13406562	0,534
9	13406799	0,534
9	13406838	0,534
9	13406867	0,534
9	13406909	0,534
9	13406937	0,534
9	13407103	0,534
9	13407164	0,534
9	13407203	0,534
9	13407338	0,534
9	13407347	0,534
9	13407854	0,534
9	13407898	0,534
9	13408233	0,534
9	13408236	0,534
9	13408302	0,534
9	13408306	0,534
9	13408534	0,534
9	13408552	0,534
9	13408616	0,534
9	13408623	0,534
9	13409327	0,534
9	13409328	0,534
9	13409879	0,534
9	13409909	0,534
9	13410311	0,534
9	13410416	0,534
9	13410699	0,534
9	13411549	0,534
9	13411692	0,534
9	13412084	0,534
9	13437923	0,603
9	13437934	0,603
9	13438055	0,603
9	13438104	0,603
9	13438404	0,586
9	13439825	0,603
9	13439947	0,603
9	13439951	0,603

9	13440301	0,586
9	13440572	0,586
9	13441129	0,586
9	13441779	0,603
9	13441781	0,603
9	13442231	0,586
9	13442267	0,586
9	13442268	0,586
9	13442294	0,586
9	13443215	0,603
9	13443433	0,603
9	13443764	0,603
9	13444193	0,586
9	13444268	0,586
9	13444327	0,586
9	13444372	0,586
9	13444376	0,586
9	13446800	0,603
9	13446957	0,586
9	13447675	0,603
9	13448590	0,655
9	13448835	0,603
9	13448870	0,655
9	13448947	0,655
9	13448952	0,655
9	13449998	0,638
9	13450065	0,586
9	13451109	0,638
9	13451308	0,638
9	13452655	0,638
9	13452685	0,638
9	13458330	0,586
9	13458666	0,586
9	13458702	0,586
9	13459146	0,586
9	13459324	0,638
9	13459470	0,586
9	13459660	0,638
9	13459670	0,586
9	13459719	0,586
9	13474805	0,586
9	13475188	0,655
9	13475400	0,586
9	13478949	0,655
9	13479000	0,655
9	13479275	0,603
9	13479987	0,655
9	13480122	0,655

9	13480155	0,655
9	13480255	0,655
9	13481196	0,586
9	13481197	0,586
9	13481227	0,638
9	13481471	0,655
9	13481493	0,603
9	13482485	0,655
9	13482492	0,655
9	13482560	0,638
9	13482598	0,638
9	13482603	0,638
9	13482658	0,638
9	13482660	0,638
9	13482665	0,638
9	13482670	0,638
9	13482692	0,638
9	13482819	0,655
9	13483776	0,586
9	13483781	0,638
9	13483829	0,655
9	13483835	0,603
9	13484064	0,638
9	13484744	0,655
9	13485162	0,603
9	13485871	0,586
9	13486000	0,655
9	13486007	0,603
9	13486008	0,603
9	13486022	0,655
9	13486167	0,638
9	13486185	0,638
9	13486416	0,638
9	13486509	0,638
9	13486551	0,655
9	13486560	0,655
9	13486658	0,655
9	13486667	0,655
9	13486760	0,655
9	13487196	0,655
9	13487926	0,638
9	13487983	0,638
9	13489907	0,586
9	13489942	0,638
9	13490033	0,603
9	13490379	0,638
9	13490653	0,655
9	13490768	0,603

9	13490966	0,603
9	13490968	0,603
9	13491260	0,603
9	13491465	0,586
9	13492016	0,603
9	13492116	0,586
9	13492145	0,586
9	13492688	0,586
9	13493798	0,603
9	13494109	0,603
9	13494469	0,603
9	13494516	0,603
9	13494592	0,586
9	13495018	0,586
9	13495025	0,586
9	13495074	0,586
9	13495177	0,603
9	13495192	0,603
9	13495287	0,603
9	13495289	0,603
9	13495305	0,603
9	13495310	0,603
9	13495357	0,603
9	13495358	0,603
9	13495361	0,603
9	13495566	0,603
9	13495579	0,603
9	13495585	0,603
9	13495622	0,603
9	13495879	0,586
9	13496568	0,586
9	13496600	0,586
9	13496601	0,586
9	13496689	0,586
9	13497360	0,603
9	13498698	0,586
9	13499321	0,603
9	13499410	0,603
9	13499443	0,586
9	13499494	0,603
9	13499863	0,586
9	13500930	0,586
9	13501309	0,586
9	13501937	0,603
9	13501948	0,603
9	13502007	0,603
9	13502027	0,603
9	13502493	0,603

9	13502510	0,655
9	13502614	0,603
9	13503867	0,603
9	13504955	0,586
9	13505069	0,586
9	13505126	0,586
9	13505319	0,586
9	13505320	0,586
9	13505406	0,603
9	13506405	0,603
9	13508293	0,603
9	13508297	0,603
9	13509393	0,586
9	13509993	0,586
9	13510073	0,586
9	13510637	0,586
9	13511269	0,586
9	13511282	0,586
9	13513314	0,586
9	13513445	0,586
9	13514277	0,586
9	13514692	0,586
9	13514941	0,586
9	13515107	0,603
9	13515129	0,655
9	13515134	0,603
9	13515162	0,603
9	13515179	0,603
9	13515183	0,603
9	13515390	0,603
9	13515426	0,655
9	13517955	0,586
9	13518186	0,586
9	13518619	0,603
9	13519231	0,603
9	13519465	0,586
9	13519514	0,586
9	13519552	0,586
9	13519560	0,586
9	13519583	0,586
9	13519647	0,586
9	13519653	0,586
9	13519732	0,586
9	13519826	0,586
9	13519834	0,586
9	13519841	0,586
9	13519893	0,603
9	13520422	0,586

9	13520800	0,603
9	13522950	0,603
9	13523382	0,586
9	13528314	0,603
9	13529186	0,586
9	13533376	0,603
9	13534806	0,586
9	13534830	0,586
9	13534846	0,586
9	13534858	0,586
9	13534876	0,586
9	13535170	0,603
9	13535198	0,603
9	13535327	0,603
9	13535473	0,586
9	13535485	0,586
9	13535575	0,603
9	13536044	0,586
9	13536617	0,603
9	13537131	0,603
9	13537615	0,586
9	13537634	0,586
9	13537815	0,586
9	13539549	0,603
9	13540180	0,586
9	13541012	0,603
9	13541306	0,586
9	13541464	0,603
9	13541800	0,603
9	13541868	0,603
9	13542010	0,586
9	13542545	0,586
9	13542676	0,586
9	13543207	0,586
9	13544697	0,586
9	13544780	0,586
9	13544925	0,586
9	13545133	0,603
9	13545216	0,586
9	13546012	0,603
9	13546094	0,603
9	13546098	0,603
9	13546581	0,586
9	13546599	0,586
9	13546956	0,603
9	13546957	0,603
9	13547203	0,586
9	13547387	0,586

9	13547583	0,586
9	13547594	0,586
9	13547626	0,586
9	13547630	0,586
9	13547833	0,603
9	13548177	0,603
9	13548852	0,603
9	13548853	0,603
9	13549137	0,603
9	13549700	0,586
9	13549714	0,586
9	13550274	0,586
9	13551081	0,603
9	13552193	0,655
9	13552232	0,655
9	13552234	0,655
9	13552235	0,655
9	13552255	0,655
9	13552468	0,586
9	13552545	0,638
9	13553205	0,655
9	13553211	0,655
9	13553229	0,655
9	13553287	0,655
9	13553296	0,655
9	13553314	0,655
9	13553586	0,655
9	13553598	0,603
9	13553611	0,603
9	13553622	0,603
9	13553664	0,603
9	13553681	0,655
9	13553852	0,603
9	13554027	0,655
9	13554036	0,603
9	13554185	0,586
9	13554238	0,638
9	13554240	0,638
9	13554248	0,586
9	13554251	0,586
9	13554374	0,638
9	13554475	0,586
9	13554829	0,638
9	13555331	0,586
9	13555340	0,638
9	13555550	0,655
9	13555688	0,586
9	13556082	0,655

9	13556105	0,655
9	13556255	0,638
9	13556372	0,655
9	13556688	0,638
9	13557396	0,638
9	13559295	0,603
9	13559298	0,603
9	13559414	0,603
9	13559524	0,603
9	13559586	0,603
9	13559952	0,603
9	13560108	0,638
9	13560474	0,655
9	13560520	0,603
9	13560536	0,603
9	13560761	0,603
9	13560775	0,603
9	13560799	0,655
9	13561078	0,655
9	13561090	0,655
9	13561213	0,655
9	13561304	0,603
9	13561390	0,603
9	13562360	0,603
9	13562735	0,655
9	13562777	0,655
9	13565072	0,638
9	13565366	0,655
9	13565372	0,655
9	13565460	0,603
9	13566090	0,655
9	13566508	0,638
9	13567017	0,638
9	13567552	0,586
9	13567581	0,586
9	13567594	0,638
9	13569111	0,586
9	13569123	0,586
9	13569216	0,655
9	13569217	0,655
9	13571496	0,638
9	13571639	0,638
9	13572224	0,603
9	13572254	0,603
9	13572258	0,603
9	13572287	0,603
9	13572301	0,603
9	13573203	0,586

9	13573253	0,586
9	13573254	0,638
9	13573776	0,638
9	13574401	0,655
9	13574460	0,655
9	13574651	0,586
9	13575437	0,655
9	13575521	0,655
9	13575535	0,655
9	13575537	0,655
9	13575541	0,655
9	13575554	0,655
9	13575583	0,655
9	13575786	0,655
9	13575968	0,655
9	13575970	0,655
9	13575998	0,655
9	13576012	0,655
9	13576106	0,638
9	13576191	0,655
9	13576986	0,638
9	13577054	0,586
9	13577107	0,638
9	13577153	0,638
9	13577218	0,638
9	13577235	0,586
9	13580015	0,638
9	13580048	0,638
9	13580146	0,638
9	13580420	0,638
9	13580762	0,655
9	13580847	0,655
9	13581106	0,638
9	13581178	0,655
9	13581197	0,655
9	13581917	0,638
9	13584193	0,603
9	13584308	0,603
9	13584738	0,638
9	13584810	0,638
9	13585372	0,603
9	13585443	0,603
9	13586150	0,603
9	13586153	0,655
9	13586367	0,603
9	13586475	0,655
9	13586885	0,655
9	13586909	0,655

9	13586912	0,655
9	13586914	0,655
9	13587082	0,638
9	13587121	0,655
9	13587126	0,655
9	13587314	0,603
9	13588215	0,638
9	13588232	0,586
9	13588498	0,603
9	13588911	0,603
9	13589011	0,603
9	13589117	0,655
9	13589583	0,603
9	13589692	0,603
9	13590625	0,638
9	13590701	0,638
9	13590723	0,586
9	13590863	0,586
9	13590967	0,603
9	13590976	0,655
9	13591019	0,655
9	13591121	0,638
9	13591995	0,638
9	13592247	0,603
9	13593290	0,603
9	13593303	0,603
9	13593322	0,603
9	13593323	0,603
9	13593506	0,603
9	13593513	0,655
9	13593517	0,603
9	13593638	0,638
9	13603006	0,655
9	13605412	0,655
9	13605670	0,603
9	13605676	0,603
9	13605703	0,603
9	13605810	0,603
9	13605838	0,603
9	13605857	0,603
9	13605872	0,603
9	13607034	0,603
9	13607221	0,603
9	13607224	0,603
9	13607370	0,586
9	13607416	0,586
9	13608090	0,603
9	13608367	0,603

9	13608405	0,603
9	13608793	0,603
9	13609006	0,603
9	13609023	0,603
9	13609158	0,603
9	13609167	0,603
9	13609453	0,603
9	13609457	0,603
9	13609553	0,586
9	13609790	0,638
9	13609861	0,638
9	13609941	0,603
9	13610073	0,586
9	13610223	0,586
9	13610639	0,586
9	13610735	0,638
9	13610946	0,638
9	13611440	0,638
9	13611536	0,638
9	13611656	0,638
9	13611919	0,655
9	13611978	0,603
9	13612005	0,655
9	13612046	0,638
9	13612345	0,655
9	13613433	0,586
9	13613814	0,603
9	13614420	0,655
9	13615121	0,638
9	13616166	0,603
9	13616641	0,638
9	13616749	0,655
9	13616752	0,655
9	13616778	0,603
9	13617829	0,603
9	13617849	0,603
9	13617983	0,655
9	13618014	0,603
9	13618130	0,638
9	13618442	0,603
9	13618458	0,655
9	13618526	0,603
9	13618553	0,603
9	13618558	0,603
9	13618574	0,603
9	13618627	0,603
9	13618648	0,603
9	13618669	0,603

9	13618832	0,603
9	13619320	0,603
9	13619391	0,655
9	13619392	0,655
9	13619397	0,655
9	13620477	0,655
9	13621491	0,638
9	13621610	0,586
9	13622643	0,603
9	13623055	0,655
9	13623060	0,603
9	13623195	0,603
9	13623202	0,655
9	13623216	0,655
9	13623225	0,603
9	13623315	0,586
9	13623360	0,586
9	13623913	0,603
9	13623946	0,603
9	13623947	0,603
9	13624694	0,586
9	13624785	0,586
9	13624885	0,638
9	13624925	0,586
9	13625024	0,586
9	13625059	0,586
9	13625062	0,586
9	13625793	0,603
9	13626629	0,655
9	13626632	0,603
9	13627020	0,655
9	13627029	0,655
9	13627057	0,655
9	13627194	0,638
9	13627198	0,638
9	13627549	0,655
9	13627772	0,638
9	13627873	0,638
9	13628197	0,586
9	13628339	0,655
9	13628341	0,655
9	13628397	0,655
9	13628496	0,655
9	13629195	0,586
9	13629902	0,655
9	13630096	0,655
9	13630353	0,638
9	13630441	0,638

9	13630504	0,638
9	13630523	0,638
9	13630830	0,603
9	13631016	0,603
9	13631019	0,603
9	13631063	0,603
9	13631075	0,603
9	13631094	0,603
9	13631344	0,603
9	13631357	0,603
9	13631555	0,603
9	13631825	0,638
9	13631833	0,638
9	13632041	0,638
9	13632155	0,638
9	13632322	0,655
9	13633527	0,603
9	13633545	0,655
9	13633567	0,655
9	13633583	0,655
9	13633613	0,603
9	13633614	0,655
9	13634022	0,586
9	13634037	0,638
9	13634165	0,603
9	13634581	0,603
9	13634678	0,586
9	13635214	0,638
9	13635518	0,655
9	13635696	0,655
9	13635701	0,655
9	13635871	0,655
9	13635875	0,655
9	13636289	0,655
9	13636290	0,655
9	13636319	0,655
9	13636322	0,655
9	13637301	0,586
9	13638432	0,655
9	13639783	0,603
9	13639784	0,603
9	13639991	0,638
9	13640142	0,638
9	13641145	0,603
9	13641596	0,655
9	13641739	0,655
9	13642153	0,603
9	13642690	0,638

9	13642698	0,586
9	13642708	0,586
9	13642740	0,638
9	13642908	0,638
9	13643279	0,638
9	13643644	0,586
9	13643836	0,655
9	13643841	0,655
9	13643847	0,655
9	13644058	0,655
9	13644068	0,655
9	13644571	0,655
9	13644588	0,603
9	13644732	0,655
9	13644734	0,655
9	13644744	0,655
9	13644859	0,603
9	13644981	0,603
9	13645031	0,603
9	13645244	0,655
9	13645337	0,655
9	13645346	0,655
9	13645397	0,638
9	13645439	0,638
9	13645465	0,638
9	13645469	0,638
9	13645472	0,638
9	13645488	0,638
9	13645588	0,655
9	13645655	0,655
9	13645889	0,655
9	13646060	0,603
9	13647021	0,603
9	13647323	0,638
9	13647425	0,603
9	13647439	0,603
9	13647485	0,586
9	13647494	0,586
9	13647513	0,586
9	13647610	0,638
9	13647677	0,638
9	13652453	0,603
9	13653710	0,586
9	13653810	0,603
9	13653861	0,586
9	13653953	0,638
9	13653968	0,586
9	13653982	0,586

9	13654006	0,638
9	13655655	0,638
9	13655671	0,586
9	13657550	0,638
9	13657726	0,638
9	13657892	0,638
9	13658461	0,638
9	13658614	0,603
9	13658686	0,603
9	13658867	0,655
9	13659845	0,638
9	13660678	0,603
9	13660786	0,603
9	13660798	0,603
9	13660819	0,603
9	13661476	0,638
9	13661528	0,638
9	13662279	0,638
9	13662304	0,638
9	13662331	0,638

Table S10. SNPs with allele-frequency difference > 0.5 in purging region in HW lineage on chromosome 11 (10.6-11.2 Mb). Loci highlighted in green were tag SNPs selected by backward-elimination analysis in this region.

Chromosome	Position	AFD
11	10752282	0,737
11	10767949	0,530
11	10780721	0,513
11	10780976	0,530
11	10780979	0,530
11	10782457	0,530
11	10782653	0,530
11	10782717	0,530
11	10782724	0,530
11	10783477	0,530
11	10784048	0,530
11	10785259	0,530
11	10785337	0,737
11	10785620	0,737
11	10785717	0,530
11	10786854	0,530
11	10786955	0,530
11	10786956	0,530
11	10786960	0,530
11	10787303	0,530
11	10787304	0,530
11	10787405	0,530
11	10787622	0,530
11	10787630	0,530
11	10787705	0,530
11	10787706	0,530
11	10787731	0,530
11	10787816	0,530
11	10787959	0,530
11	10788547	0,737
11	10788979	0,737
11	10789047	0,513
11	10789411	0,513
11	10789596	0,513
11	10789631	0,737
11	10789718	0,513
11	10790223	0,737
11	10790246	0,513
11	10790773	0,737
11	10790839	0,737
11	10790845	0,737
11	10790908	0,737

11	10791012	0,737
11	10791536	0,737
11	10791540	0,737
11	10792033	0,513
11	10792275	0,737
11	10792355	0,737
11	10792625	0,513
11	10792797	0,737
11	10794106	0,530
11	10796935	0,530
11	10796942	0,530
11	10796964	0,530
11	10797435	0,513
11	10797451	0,513
11	10797577	0,530
11	10797604	0,530
11	10799070	0,530
11	10799482	0,513
11	10800190	0,530
11	10801113	0,530
11	10801325	0,513
11	10801350	0,513
11	10801354	0,513
11	10801422	0,513
11	10801543	0,513
11	10801858	0,530
11	10801907	0,530
11	10801940	0,530
11	10802624	0,737
11	10803377	0,737
11	10803771	0,737
11	10803841	0,737
11	10803945	0,737
11	10803948	0,737
11	10803979	0,737
11	10803988	0,737
11	10804010	0,737
11	10804444	0,513
11	10805329	0,530
11	10806327	0,513
11	10807490	0,530
11	10807676	0,530
11	10807952	0,737
11	10807956	0,737
11	10807957	0,737
11	10807969	0,737
11	10808164	0,737
11	10808986	0,513

11	10809978	0,737
11	10810516	0,737
11	10810535	0,737
11	10810540	0,530
11	10810557	0,737
11	10810637	0,737
11	10810842	0,737
11	10810886	0,737
11	10810941	0,737
11	10811033	0,737
11	10811656	0,530
11	10811676	0,530
11	10812647	0,530
11	10812721	0,737
11	10812922	0,737
11	10812977	0,737
11	10813029	0,727
11	10813202	0,530
11	10813207	0,737
11	10813678	0,530
11	10814865	0,737
11	10815088	0,737
11	10815204	0,530
11	10815309	0,737
11	10815346	0,737
11	10815350	0,737
11	10815677	0,513
11	10815750	0,513
11	10815860	0,737
11	10816661	0,530
11	10816695	0,530
11	10816782	0,513
11	10816804	0,513
11	10816934	0,530
11	10817040	0,530
11	10817055	0,737
11	10817223	0,530
11	10817238	0,530
11	10817733	0,513
11	10817804	0,513
11	10817840	0,513
11	10817898	0,513
11	10817947	0,513
11	10821146	0,737
11	10821411	0,727
11	10821437	0,727
11	10821440	0,727
11	10821932	0,737

11	10822091	0,727
11	10822478	0,727
11	10822588	0,737
11	10823741	0,737
11	10823743	0,727
11	10823926	0,727
11	10824106	0,727
11	10837928	0,530
11	10837939	0,530
11	10837998	0,513
11	10838002	0,513
11	10838148	0,530
11	10838169	0,530
11	10838568	0,530
11	10838955	0,513
11	10839092	0,530
11	10839139	0,530
11	10840571	0,513
11	10841501	0,513
11	10841540	0,530
11	10841993	0,513
11	10846116	0,513
11	10846236	0,513
11	10846688	0,513
11	10852113	0,513
11	10852200	0,513
11	10852229	0,513
11	10852425	0,530
11	10852544	0,513
11	10852549	0,513
11	10853097	0,530
11	10853602	0,513
11	10853633	0,513
11	10854252	0,513
11	10855980	0,513
11	10859250	0,513
11	10859648	0,513
11	10859885	0,513
11	10862542	0,530
11	10862543	0,530
11	10862890	0,530
11	10862958	0,513
11	10863630	0,530
11	10863728	0,530
11	10864120	0,513
11	10864250	0,513
11	10864301	0,513
11	10864499	0,513

11	10865662	0,530
11	10865965	0,530
11	10866497	0,530
11	10866785	0,530
11	10867353	0,530
11	10867454	0,530
11	10867456	0,530
11	10867543	0,530
11	10867580	0,530
11	10867739	0,530
11	10868298	0,530
11	10869817	0,530
11	10869828	0,530
11	10869851	0,530
11	10870090	0,530
11	10870096	0,530
11	10870139	0,530
11	10870400	0,530
11	10870694	0,530
11	10871954	0,513
11	10874165	0,513
11	10875132	0,513
11	10876127	0,513
11	10876276	0,530
11	10876277	0,530
11	10876471	0,530
11	10876494	0,530
11	10876676	0,530
11	10876721	0,530
11	10877005	0,530
11	10877246	0,513
11	10877386	0,513
11	10877876	0,530
11	10877917	0,530
11	10878685	0,530
11	10878688	0,530
11	10878781	0,513
11	10879638	0,530
11	10880246	0,513
11	10880669	0,530
11	10880903	0,513
11	10880957	0,513
11	10881572	0,530
11	10881655	0,530
11	10884221	0,530
11	10884397	0,530
11	10884970	0,513
11	10885502	0,513

11	10885741	0,530
11	10885745	0,530
11	10886842	0,513
11	10886905	0,530
11	10886908	0,530
11	10887444	0,530
11	10888086	0,530
11	10890078	0,530
11	10890308	0,513
11	10891303	0,530
11	10891726	0,530
11	10892317	0,530
11	10892735	0,530
11	10893921	0,530
11	10894448	0,513
11	10895757	0,513
11	10896453	0,513
11	10896564	0,513
11	10896632	0,530
11	10896636	0,530
11	10896776	0,513
11	10896974	0,513
11	10897059	0,513
11	10897346	0,513
11	10898609	0,530
11	10898841	0,530
11	10900601	0,513
11	10900946	0,513
11	10901391	0,530
11	10901738	0,513
11	10902242	0,530
11	10902245	0,530
11	10905011	0,513
11	10907308	0,513
11	10910426	0,530
11	10913315	0,513
11	10913353	0,513
11	10913402	0,513
11	10913403	0,513
11	10914665	0,513
11	10914967	0,530
11	10915523	0,513
11	10915754	0,530
11	10916008	0,513
11	10916091	0,513
11	10916107	0,513
11	10916637	0,513
11	10916658	0,513

11	10916719	0,513
11	10917615	0,513
11	10917904	0,530
11	10918229	0,513
11	10921045	0,513
11	10923056	0,513
11	10923076	0,513
11	10923116	0,513
11	10923900	0,530
11	10924784	0,513
11	10925149	0,530
11	10928216	0,530
11	10928455	0,513
11	10929781	0,530
11	10930363	0,513
11	10931242	0,513
11	10931652	0,513
11	10931663	0,513
11	10931861	0,513
11	10932311	0,530
11	10932352	0,530
11	10932429	0,530
11	10934126	0,513
11	10934138	0,513
11	10934291	0,530
11	10934400	0,530
11	10934472	0,530
11	10934670	0,530
11	10934718	0,530
11	10934968	0,530
11	10935154	0,530
11	10935273	0,530
11	10935284	0,530
11	10935566	0,530
11	10935590	0,530
11	10935718	0,530
11	10935736	0,530
11	10936029	0,530
11	10936030	0,530
11	10936034	0,530
11	10936604	0,530
11	10936606	0,530
11	10938108	0,513
11	10938318	0,513
11	10938324	0,513
11	10938957	0,530
11	10941369	0,530
11	10941386	0,530

11	10941704	0,530
11	10941981	0,513
11	10942405	0,513
11	10942457	0,530
11	10942693	0,530
11	10942716	0,530
11	10943407	0,530
11	10943600	0,513
11	10945116	0,530
11	10945969	0,513
11	10946122	0,513
11	10946377	0,513
11	10946647	0,530
11	10946803	0,530
11	10947075	0,530
11	10947822	0,530
11	10947906	0,530
11	10947923	0,530
11	10949415	0,530
11	10949461	0,530
11	10950412	0,513
11	10950743	0,530
11	10950762	0,530
11	10950788	0,727
11	10952281	0,513
11	10952747	0,530
11	10953262	0,727
11	10953408	0,727
11	10954346	0,530
11	10954358	0,727
11	10954488	0,530
11	10955086	0,513
11	10955575	0,727
11	10955939	0,727
11	10957853	0,727
11	10959366	0,727
11	10959419	0,727
11	10959585	0,530
11	10959587	0,530
11	10959662	0,530
11	10959667	0,530
11	10959687	0,530
11	10959709	0,530
11	10960087	0,513
11	10960119	0,513
11	10960153	0,513
11	10960325	0,530
11	10961774	0,727

11	10962174	0,513
11	10963161	0,727
11	10963346	0,530
11	10963352	0,530
11	10963357	0,530
11	10963520	0,530
11	10964270	0,727
11	10964369	0,530
11	10964588	0,727
11	10964947	0,727
11	10966134	0,727
11	10966615	0,513
11	10966619	0,727
11	10967047	0,530
11	10967052	0,530
11	10967357	0,727
11	10967806	0,727
11	10968077	0,530
11	10968078	0,530
11	10968588	0,727
11	10968589	0,727
11	10969032	0,727
11	10969075	0,727
11	10969089	0,727
11	10969181	0,530
11	10969377	0,530
11	10969378	0,727
11	10969891	0,727
11	10970290	0,530
11	10970479	0,727
11	10970495	0,513
11	10970660	0,513
11	10972354	0,727
11	10972807	0,727
11	10972810	0,727
11	10972990	0,513
11	10973749	0,513
11	10973807	0,513
11	10974067	0,513
11	10974651	0,530
11	10974887	0,530
11	10975527	0,530
11	10975534	0,530
11	10975911	0,530
11	10976041	0,530
11	10976815	0,530
11	10976961	0,530
11	10977136	0,530

11	10977445	0,513
11	10977872	0,513
11	10977919	0,513
11	10977990	0,513
11	10978371	0,530
11	10978506	0,530
11	10978677	0,513
11	10978792	0,513
11	10978844	0,513
11	10979528	0,513
11	10979544	0,513
11	10981399	0,530
11	10982246	0,530
11	10982458	0,530
11	10984026	0,513
11	10984669	0,530
11	10985025	0,513
11	10985225	0,530
11	10985792	0,513
11	10985945	0,530
11	10986002	0,530
11	10987055	0,727
11	10987057	0,530
11	10987417	0,530
11	10987510	0,530
11	10987588	0,727
11	10987719	0,727
11	10987770	0,727
11	10988409	0,530
11	10988433	0,727
11	10988507	0,727
11	10988642	0,727
11	10988649	0,727
11	10989140	0,727
11	10989471	0,727
11	10990100	0,727
11	10990379	0,727
11	10990533	0,727
11	10990564	0,727
11	10990994	0,727
11	10991142	0,727
11	10991320	0,727
11	10991422	0,727
11	10991454	0,727
11	10991833	0,727
11	10992547	0,727
11	10992680	0,727
11	10992698	0,727

11	10992715	0,727
11	10992910	0,727
11	10992934	0,727
11	10992964	0,727
11	10993081	0,727
11	10993736	0,727
11	10993738	0,727
11	10993748	0,727
11	10994410	0,513
11	10994432	0,727
11	10994606	0,727
11	10994613	0,727
11	10994786	0,727
11	10994789	0,727
11	10994946	0,513
11	10994979	0,513
11	10995065	0,727
11	10995311	0,727
11	10995523	0,530
11	10995848	0,530
11	10995968	0,727
11	10996259	0,727
11	10996412	0,727
11	10996423	0,727
11	10996430	0,727
11	10996433	0,727
11	10996439	0,727
11	10996456	0,727
11	10996487	0,727
11	10996502	0,727
11	10996613	0,530
11	10996776	0,727
11	10996971	0,727
11	10997222	0,727
11	10998018	0,727
11	10998102	0,727
11	10998340	0,530
11	10998776	0,530
11	10998793	0,530
11	10998794	0,530
11	10999285	0,513
11	10999409	0,530
11	10999411	0,530
11	10999817	0,727
11	10999875	0,530
11	11000624	0,513
11	11001215	0,513
11	11001474	0,727

11	11001818	0,513
11	11002148	0,530
11	11003436	0,727
11	11003544	0,727
11	11003875	0,727
11	11004288	0,727
11	11004349	0,727
11	11004460	0,530
11	11004814	0,727
11	11004819	0,727
11	11006167	0,513
11	11006750	0,513
11	11007102	0,530
11	11007277	0,513
11	11007572	0,513
11	11007810	0,513
11	11008221	0,530
11	11008223	0,530
11	11008490	0,530
11	11008504	0,530
11	11008517	0,530
11	11008535	0,530
11	11008572	0,513
11	11009587	0,513
11	11009922	0,530
11	11010051	0,530
11	11010330	0,530
11	11010538	0,530
11	11010557	0,530
11	11010558	0,530
11	11010663	0,513
11	11010681	0,513
11	11011382	0,513
11	11012529	0,513
11	11012708	0,513
11	11012734	0,513
11	11013023	0,513
11	11013156	0,513
11	11013194	0,513
11	11013228	0,513
11	11013847	0,513
11	11014194	0,530
11	11014199	0,530
11	11014253	0,530
11	11014265	0,530
11	11014917	0,530
11	11014971	0,513
11	11015185	0,513

11	11015375	0,530
11	11016491	0,530
11	11016513	0,530
11	11017373	0,530
11	11017709	0,513
11	11017765	0,513
11	11018973	0,513
11	11019291	0,530
11	11019864	0,530
11	11019992	0,530
11	11020407	0,513
11	11020534	0,530
11	11020946	0,530
11	11020952	0,530
11	11021259	0,530
11	11022723	0,530
11	11023065	0,530
11	11024606	0,727
11	11025090	0,513
11	11027002	0,530
11	11032273	0,513
11	11034704	0,530
11	11034705	0,530
11	11035434	0,530
11	11035456	0,530
11	11036267	0,513
11	11036797	0,530
11	11036799	0,530
11	11036985	0,513
11	11037115	0,513
11	11037191	0,513
11	11037510	0,513
11	11037768	0,513
11	11038187	0,530
11	11038631	0,513
11	11038643	0,513
11	11039796	0,513
11	11039878	0,530
11	11040046	0,530
11	11040422	0,530
11	11040677	0,530
11	11040948	0,530
11	11042425	0,530
11	11062513	0,530
11	11062517	0,530
11	11065538	0,530
11	11068902	0,530
11	11068948	0,530

11	11069082	0,530
11	11069180	0,530
11	11069791	0,530
11	11070079	0,513
11	11070144	0,513
11	11070155	0,513
11	11070302	0,513
11	11070317	0,513
11	11070542	0,530
11	11071901	0,530
11	11071905	0,530
11	11072620	0,530
11	11072880	0,513
11	11073303	0,530
11	11073304	0,530
11	11075788	0,513
11	11075803	0,513
11	11075910	0,513
11	11076759	0,530
11	11077037	0,513
11	11078220	0,513
11	11078660	0,513
11	11079095	0,530
11	11079104	0,530
11	11079296	0,513
11	11079329	0,513
11	11079917	0,513
11	11081837	0,513
11	11081958	0,513
11	11081973	0,513
11	11081982	0,513
11	11082321	0,530
11	11082334	0,530
11	11082335	0,530
11	11082499	0,530
11	11082511	0,530
11	11082567	0,530
11	11083659	0,513
11	11083684	0,513
11	11083719	0,513
11	11083728	0,513
11	11083744	0,513
11	11083782	0,513
11	11083795	0,513
11	11083840	0,530
11	11083912	0,513
11	11084475	0,513
11	11086318	0,530

11	11086339	0,530
11	11086383	0,530
11	11086398	0,530
11	11086794	0,513
11	11086887	0,513
11	11087134	0,513
11	11087238	0,513
11	11087377	0,530
11	11088763	0,530
11	11088858	0,530
11	11088859	0,530
11	11089570	0,513
11	11089850	0,513
11	11089879	0,513
11	11090958	0,530
11	11092201	0,513
11	11092209	0,513
11	11092247	0,513
11	11092253	0,513
11	11092254	0,513
11	11092744	0,513
11	11093039	0,530
11	11093060	0,530
11	11093625	0,530
11	11093967	0,513
11	11094924	0,513
11	11095233	0,513
11	11095246	0,513
11	11095264	0,513
11	11096287	0,513
11	11096522	0,513
11	11096761	0,513
11	11097695	0,513
11	11098293	0,737
11	11098306	0,513
11	11098771	0,513
11	11099662	0,530
11	11099671	0,530
11	11099719	0,530
11	11099724	0,530
11	11099758	0,530
11	11099772	0,530
11	11099774	0,530
11	11099776	0,530
11	11099785	0,530
11	11100082	0,530
11	11100090	0,530
11	11100097	0,530

11	11100564	0,513
11	11101336	0,513
11	11101361	0,513
11	11101366	0,513
11	11101369	0,513
11	11101868	0,530
11	11103342	0,737
11	11103621	0,737
11	11103773	0,737
11	11103785	0,737
11	11103791	0,737
11	11104483	0,737
11	11104581	0,513
11	11104780	0,513
11	11104783	0,513
11	11104784	0,513
11	11104801	0,513
11	11107029	0,530
11	11107331	0,513
11	11107358	0,513
11	11107988	0,737
11	11108448	0,737
11	11108526	0,737
11	11109015	0,737
11	11109662	0,530
11	11109750	0,530
11	11111242	0,737
11	11111340	0,737
11	11111561	0,737
11	11111578	0,737
11	11112078	0,737
11	11112117	0,737
11	11112198	0,737
11	11112404	0,737
11	11112422	0,737
11	11112454	0,737
11	11112487	0,513
11	11113082	0,737
11	11113514	0,513
11	11114069	0,530
11	11114073	0,530
11	11114935	0,737
11	11115210	0,737
11	11115748	0,737
11	11116073	0,513
11	11116139	0,737
11	11117516	0,617
11	11117755	0,617

11	11117756	0,617
11	11118222	0,623
11	11118483	0,623
11	11119177	0,617
11	11119236	0,623
11	11120440	0,617
11	11120644	0,617
11	11120686	0,617
11	11120806	0,623
11	11120944	0,617
11	11120994	0,623
11	11121274	0,617
11	11121286	0,617
11	11121318	0,617
11	11121324	0,623
11	11121405	0,617
11	11121825	0,617
11	11121851	0,617
11	11121900	0,617
11	11122370	0,623
11	11122432	0,623
11	11122519	0,617
11	11122533	0,617
11	11125287	0,623
11	11125290	0,617
11	11158770	0,623
11	11159133	0,623
11	11159277	0,623
11	11159420	0,623
11	11159574	0,617
11	11159881	0,617
11	11160096	0,617
11	11160217	0,617
11	11160219	0,617
11	11161187	0,623
11	11161398	0,623
11	11161472	0,623
11	11161649	0,623
11	11161815	0,623
11	11161960	0,623
11	11161994	0,623
11	11162043	0,623
11	11162197	0,623
11	11162384	0,623
11	11162499	0,623
11	11162557	0,623
11	11162568	0,623
11	11162688	0,623

11	11162746	0,623
11	11163268	0,623
11	11163283	0,623
11	11163416	0,623
11	11163718	0,623
11	11165089	0,623
11	11165206	0,623
11	11165435	0,623
11	11165488	0,623
11	11165585	0,623
11	11165636	0,623
11	11165726	0,623
11	11165757	0,623
11	11165818	0,623
11	11165848	0,623
11	11165875	0,623
11	11165890	0,623
11	11166036	0,623
11	11166094	0,623
11	11166116	0,623
11	11166163	0,623
11	11166204	0,623
11	11166205	0,617
11	11166613	0,623
11	11166638	0,623
11	11166783	0,623
11	11168423	0,623
11	11168425	0,623
11	11169724	0,623
11	11169949	0,623
11	11170554	0,623
11	11170635	0,623
11	11170969	0,623
11	11171045	0,623
11	11171546	0,617
11	11172150	0,623
11	11172166	0,623
11	11172233	0,623
11	11172261	0,623
11	11172558	0,623
11	11173252	0,623
11	11173283	0,623
11	11173343	0,623
11	11174462	0,623
11	11175562	0,623
11	11175577	0,623
11	11175578	0,623
11	11176166	0,623

11	11176997	0,623
11	11178913	0,623
11	11180458	0,623
11	11180872	0,623
11	11181545	0,623
11	11182954	0,623
11	11183177	0,623
11	11183255	0,623
11	11187036	0,623
11	11188159	0,617
11	11188195	0,617
11	11193646	0,617
11	11193682	0,617
11	11193817	0,617
11	11194727	0,617
11	11195009	0,617
11	11195579	0,623
11	11195765	0,617
11	11195766	0,617
11	11195924	0,617
11	11198573	0,623
11	11199373	0,623
11	11200132	0,617
11	11200356	0,617
11	11201901	0,623
11	11202143	0,617
11	11202272	0,617
11	11202943	0,617
11	11203023	0,617
11	11203136	0,617
11	11204217	0,623
11	11204624	0,623
11	11204702	0,623
11	11204847	0,623
11	11204904	0,623
11	11245902	0,613
11	11246208	0,623
11	11246740	0,603
11	11246802	0,623
11	11246821	0,623
11	11247074	0,603
11	11247518	0,623
11	11247782	0,603
11	11247792	0,623
11	11248133	0,613
11	11248171	0,613
11	11248221	0,613
11	11248227	0,603

11	11248229	0,613
11	11248346	0,613
11	11248354	0,613
11	11248382	0,603
11	11248447	0,603
11	11248503	0,603
11	11248587	0,623
11	11248961	0,623
11	11249588	0,603
11	11251912	0,603
11	11252308	0,603
11	11252311	0,638
11	11252356	0,603
11	11252407	0,628
11	11252869	0,613
11	11253263	0,623
11	11253296	0,623
11	11253527	0,623
11	11253536	0,623
11	11253545	0,613
11	11253650	0,623
11	11254276	0,628
11	11254406	0,623
11	11254489	0,628
11	11254620	0,628
11	11255095	0,623
11	11255621	0,623
11	11255834	0,623
11	11256119	0,623
11	11256350	0,628
11	11256653	0,628
11	11256776	0,623
11	11256960	0,623
11	11257435	0,623
11	11258032	0,628
11	11259059	0,623
11	11259313	0,623
11	11259378	0,617
11	11259941	0,623
11	11259976	0,623
11	11260044	0,623
11	11260323	0,628
11	11260512	0,623
11	11261212	0,623
11	11261868	0,623
11	11261958	0,623
11	11261963	0,623
11	11262215	0,623

11	11262843	0,628
11	11263242	0,628
11	11263263	0,628
11	11263410	0,628
11	11263456	0,628
11	11263763	0,628
11	11264370	0,628
11	11264606	0,628
11	11265358	0,623
11	11265574	0,623
11	11266256	0,623
11	11266434	0,628
11	11271668	0,628
11	11271848	0,623
11	11272001	0,623
11	11273584	0,623
11	11277509	0,623
11	11278110	0,623
11	11278128	0,623

Table S11. SNPs with allele-frequency difference > 0.5 in purging region in HW lineage on chromosome 20 (10.6-10.7 Mb). Loci highlighted in green were tag SNPs selected by backward-elimination analysis in this region.

Chromosome	Position	AFD
20	10666379	0,584
20	10666401	0,584
20	10666405	0,584
20	10666480	0,584
20	10666681	0,756
20	10667370	0,722
20	10667575	0,722
20	10667817	0,544
20	10668030	0,722
20	10668353	0,561
20	10668737	0,722
20	10668758	0,756
20	10669071	0,756
20	10669160	0,756
20	10670059	0,756
20	10670133	0,756
20	10670134	0,756
20	10670532	0,618
20	10670663	0,618
20	10671067	0,618
20	10671147	0,618
20	10671528	0,584
20	10671690	0,584
20	10671955	0,618
20	10671976	0,584
20	10672006	0,618
20	10672027	0,584
20	10672079	0,756
20	10673132	0,756
20	10674284	0,722
20	10674488	0,756
20	10675275	0,544
20	10676908	0,618
20	10676992	0,618
20	10677008	0,618
20	10677015	0,618
20	10677032	0,756
20	10677033	0,756
20	10677856	0,756
20	10679133	0,584
20	10679199	0,618
20	10679508	0,756
20	10679799	0,756
20	10680453	0,756

20	10681113	0,756
20	10681200	0,756
20	10681426	0,756
20	10681505	0,584
20	10681580	0,722
20	10681733	0,756
20	10681734	0,584
20	10681777	0,722
20	10681815	0,756
20	10682220	0,756
20	10683397	0,722
20	10683918	0,756
20	10684497	0,578
20	10685645	0,578
20	10685893	0,756
20	10686088	0,756
20	10686093	0,756
20	10686696	0,756
20	10686784	0,756
20	10686846	0,584
20	10686869	0,584
20	10687583	0,618
20	10688489	0,756
20	10689504	0,722
20	10689579	0,756
20	10689690	0,618
20	10689738	0,756
20	10690403	0,756
20	10690458	0,584
20	10690466	0,618
20	10690475	0,618
20	10690583	0,756
20	10690705	0,578
20	10690733	0,578
20	10691133	0,578
20	10691241	0,584
20	10691942	0,756
20	10692256	0,756
20	10692761	0,561
20	10692840	0,756
20	10692982	0,561
20	10692983	0,561
20	10693029	0,756
20	10693074	0,722
20	10693494	0,756
20	10694352	0,561
20	10694362	0,561
20	10694698	0,544

20	10694866	0,756
20	10694883	0,618
20	10695265	0,756
20	10695286	0,561
20	10695415	0,756
20	10695430	0,756
20	10695468	0,756
20	10695687	0,756
20	10695798	0,561
20	10696021	0,756
20	10696133	0,722
20	10696574	0,578
20	10696585	0,578
20	10696616	0,578
20	10696694	0,756
20	10696839	0,756
20	10697106	0,756
20	10697486	0,618
20	10697489	0,618
20	10698092	0,756
20	10698564	0,618
20	10698810	0,756
20	10698949	0,756
20	10699052	0,756
20	10699774	0,756
20	10700425	0,756
20	10700464	0,561
20	10700465	0,561
20	10701679	0,756
20	10702012	0,756
20	10702233	0,756
20	10702244	0,561
20	10702439	0,756
20	10703091	0,722
20	10703278	0,756
20	10703922	0,527
20	10704869	0,756
20	10705422	0,756
20	10705665	0,756
20	10705762	0,722
20	10705834	0,722
20	10705866	0,722
20	10705980	0,756
20	10706022	0,578
20	10706169	0,544
20	10706403	0,722
20	10706424	0,756
20	10706545	0,527

20	10706730	0,756
20	10706744	0,756
20	10707959	0,544
20	10708043	0,722
20	10710190	0,756
20	10710332	0,722
20	10710456	0,544
20	10710474	0,544
20	10710687	0,756
20	10710814	0,756
20	10711007	0,561
20	10711063	0,722
20	10711098	0,527
20	10711138	0,527
20	10711154	0,756
20	10711474	0,544
20	10711517	0,722
20	10711694	0,756
20	10711735	0,756
20	10712151	0,756
20	10712289	0,544
20	10712420	0,756
20	10712486	0,756
20	10712698	0,578
20	10713196	0,578
20	10713571	0,756
20	10713574	0,756
20	10713600	0,756
20	10713625	0,756
20	10713630	0,756
20	10713636	0,756
20	10713644	0,756
20	10713685	0,756
20	10713707	0,756
20	10713711	0,756
20	10713812	0,722
20	10714422	0,756
20	10714425	0,756
20	10714718	0,756
20	10715095	0,618
20	10715399	0,561
20	10715401	0,561
20	10715740	0,618
20	10716887	0,756
20	10717267	0,722
20	10717433	0,756
20	10717634	0,618
20	10718530	0,722

20	10722552	0,618
20	10722611	0,756
20	10724776	0,756
20	10725644	0,561
20	10726091	0,756
20	10726094	0,609
20	10726714	0,756
20	10726737	0,722
20	10726743	0,747
20	10726952	0,756
20	10727568	0,756
20	10729503	0,634
20	10729674	0,781
20	10729887	0,772
20	10730019	0,772
20	10735190	0,806
20	10735238	0,578
20	10735598	0,781
20	10735664	0,668
20	10735685	0,806
20	10735787	0,643
20	10737007	0,806
20	10737060	0,806
20	10737192	0,578
20	10737362	0,668
20	10738314	0,561
20	10738326	0,806
20	10738382	0,806
20	10740762	0,578
20	10740930	0,561
20	10741543	0,668
20	10741964	0,772
20	10742547	0,772
20	10742834	0,668
20	10742889	0,634
20	10743581	0,806
20	10743752	0,806
20	10744503	0,772
20	10744554	0,772
20	10745054	0,527
20	10745058	0,527
20	10745060	0,527
20	10745263	0,806
20	10745341	0,772
20	10745712	0,806
20	10745769	0,772
20	10748859	0,668

Table S12, Haplotype frequencies for the major haplotypes segregating in the HWS41/LWS41 birds used as founders for the AIL where associations to 56 day body weights were performed in generations F2 - F15

Haplotype	Lineage	Location Chromosome_Position	F_2	F_3	F_4	F_5	F_6	F_7	F_8	F_9	F_{10}	F_{11}	F_{13}	F_{14}	F_{15}	HWR9	HWS41	HWS50	HWS53	LWR9	LWS41	LWS50	LWS53
$Hap1_{HC8-2}$	HW	8_10Mb	0,7	0,7	0,7	0,7	0,6	0,6	0,6	0,7	0,8	0,8	0,7	0,7	0,7	0,0	0,8	0,4	0,6	1,0	0,9	1,0	1,0
$Hap2_{HC8-2}$	HW	8_10Mb	0,0	0,0	0,0	0,0	0,0	0,0	0,0	0,0	0,0	0,0	0,0	0,1	0,0	0,6	0,0	0,1	0,0	0,0	0,0	0,0	0,0
$Hap3_{HC8-2}$	HW	8_10Mb	0,1	0,1	0,1	0,1	0,2	0,2	0,2	0,2	0,1	0,1	0,1	0,1	0,2	0,3	0,1	0,3	0,2	0,0	0,1	0,0	0,0
$Hap1_{HC20-10}$	HW	20_2Mb	0,5	0,5	0,5	0,5	0,5	0,5	0,5	0,4	0,5	0,5	0,5	0,4	0,4	0,8	0,1	0,3	0,3	1,0	1,0	1,0	1,0
$Hap2_{HC20-10}$	HW	20_2Mb	0,3	0,2	0,3	0,2	0,2	0,3	0,2	0,2	0,2	0,1	0,1	0,1	0,1	0,2	0,3	0,5	0,3	0,0	0,0	0,0	0,0
$Hap3_{HC20-10}$	HW	20_2Mb	0,2	0,2	0,2	0,2	0,3	0,3	0,4	0,4	0,4	0,4	0,4	0,4	0,4	0,0	0,5	0,2	0,4	0,0	0,0	0,0	0,0
$Hap1_{HC6-17}$	LW	6_17Mb	0,5	0,5	0,5	0,5	0,5	0,6	0,7	0,6	0,6	0,6	0,6	0,6	0,6	0,5	0,4	0,2	0,3	0,2	0,8	0,8	1,0
$Hap2_{HC6-17}$	LW	6_17Mb	0,5	0,5	0,5	0,5	0,5	0,4	0,3	0,4	0,5	0,4	0,4	0,4	0,4	0,5	0,6	0,8	0,7	0,8	0,2	0,3	0,0
