# Supplementary material for: Haplotype Purging after Relaxation of Selection in Lines of Chickens That Had Undergone Long-Term Selection for High and Low Body Weight
Source: Genes (Basel). 2020 Jun 8;11(6):630. doi: 10.3390/genes11060630 (PMC7349872; doi:10.3390/genes11060630)
Supplement: Supplementary file 1 [file genes-11-00630-s001.zip › Supplementary_Figures_S1-S15.pdf]

Supplementary figures S1-S15

Haplotype purging after relaxation of selection in lines of chickens that had undergone long-term selection for high and low body weight

Yunzhou Yang, Yanjun Zan, Christa F. Honaker, Paul B. Siegel, and Örjan Carlborg

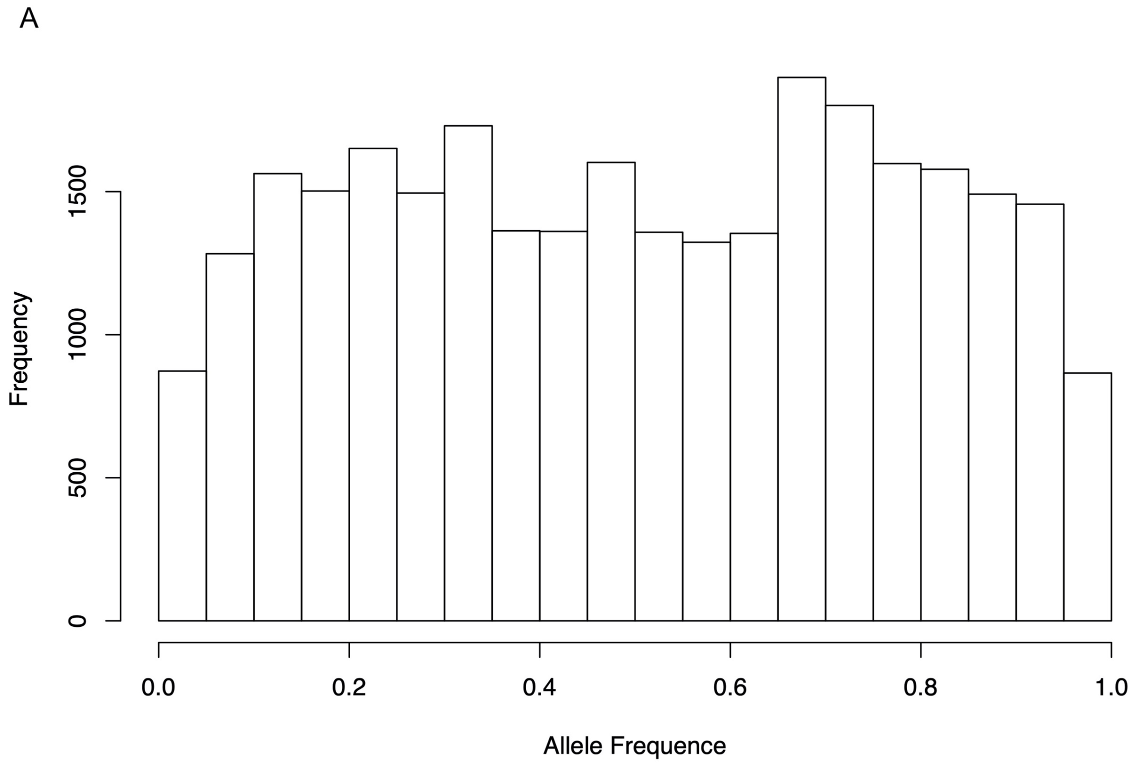

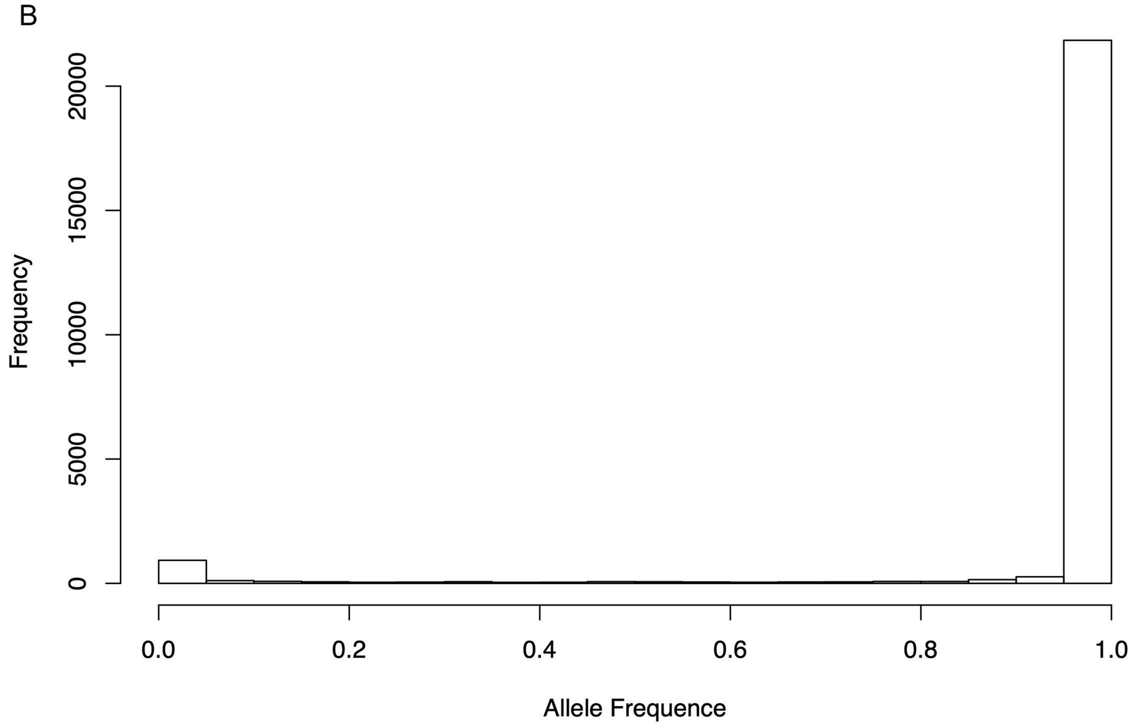

Figure S1. Allele frequency of single nucleotide polymorphisms (SNP) genotyped by the 60-K chicken chip. **A:** Distributions of allele frequency for 29,147 SNPs which were kept for imputation, **B:** Distributions of allele frequency for 24,166 SNPs which were filtered out.

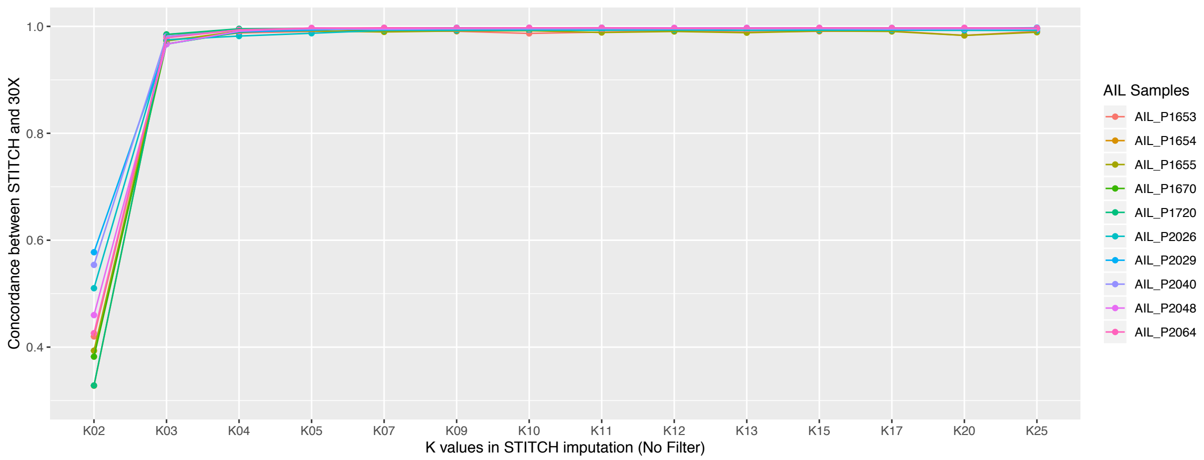

Figure S2. Evaluation of genotyping quality from imputation using different K-values in the *STITCH* software [25]. X-axis are the values tested for K. The Y-axis are the corresponding concordances between the genotypes scored using ~30X-depth sequencing and the *STITCH* genotypes imputed on the same individuals after down-sampling to ~0.4X using *seqtk* (https://github.com/lh3/seqtk).

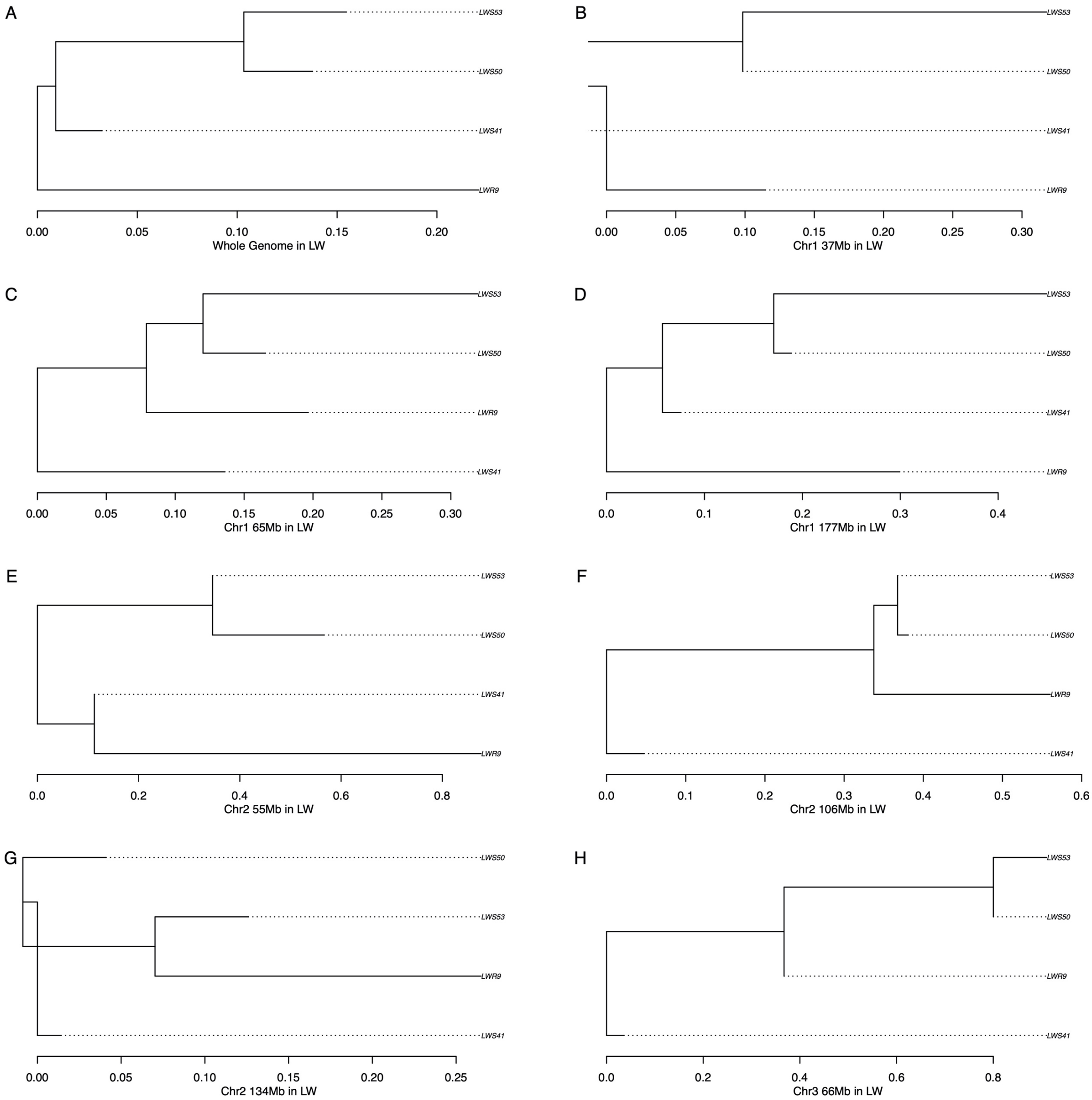

Figure S3. Hierarchical *hapFLK* trees for the LW lineage of the Virginia lines. **A** shows the tree constructed using whole-genome data for reference. **B**-**H** are the local trees constructed using genotype data for the *hapFLK* significant regions on chromosomes 1, 2, and 3.

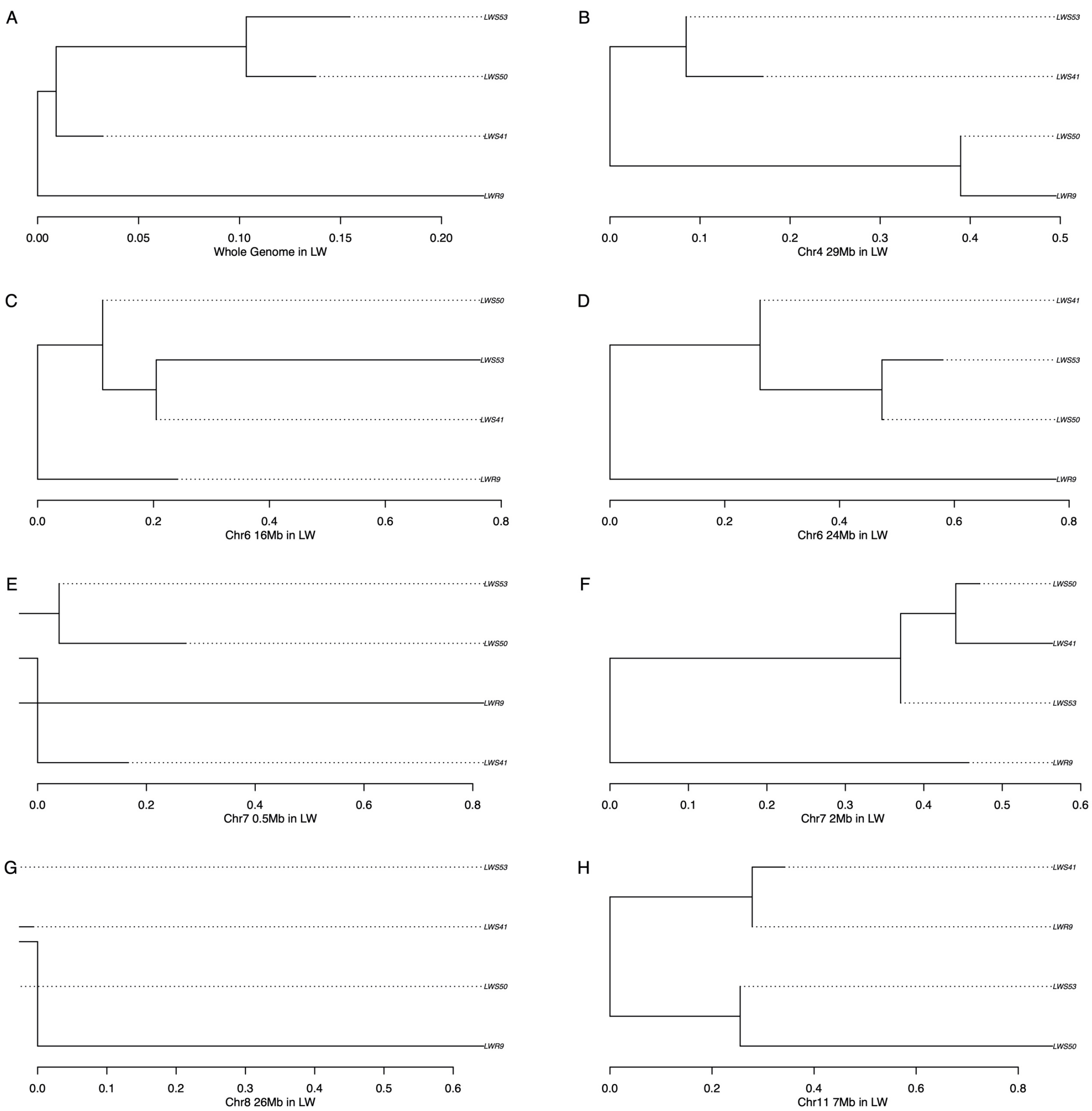

Figure S4. Hierarchical *hapFLK* trees for the LW lineage of the Virginia lines. **A** shows the tree constructed using whole-genome data for reference. **B**-**H** are the local trees constructed using genotype data for the *hapFLK* significant regions on chromosomes 4, 6, 7, 8, and 11.

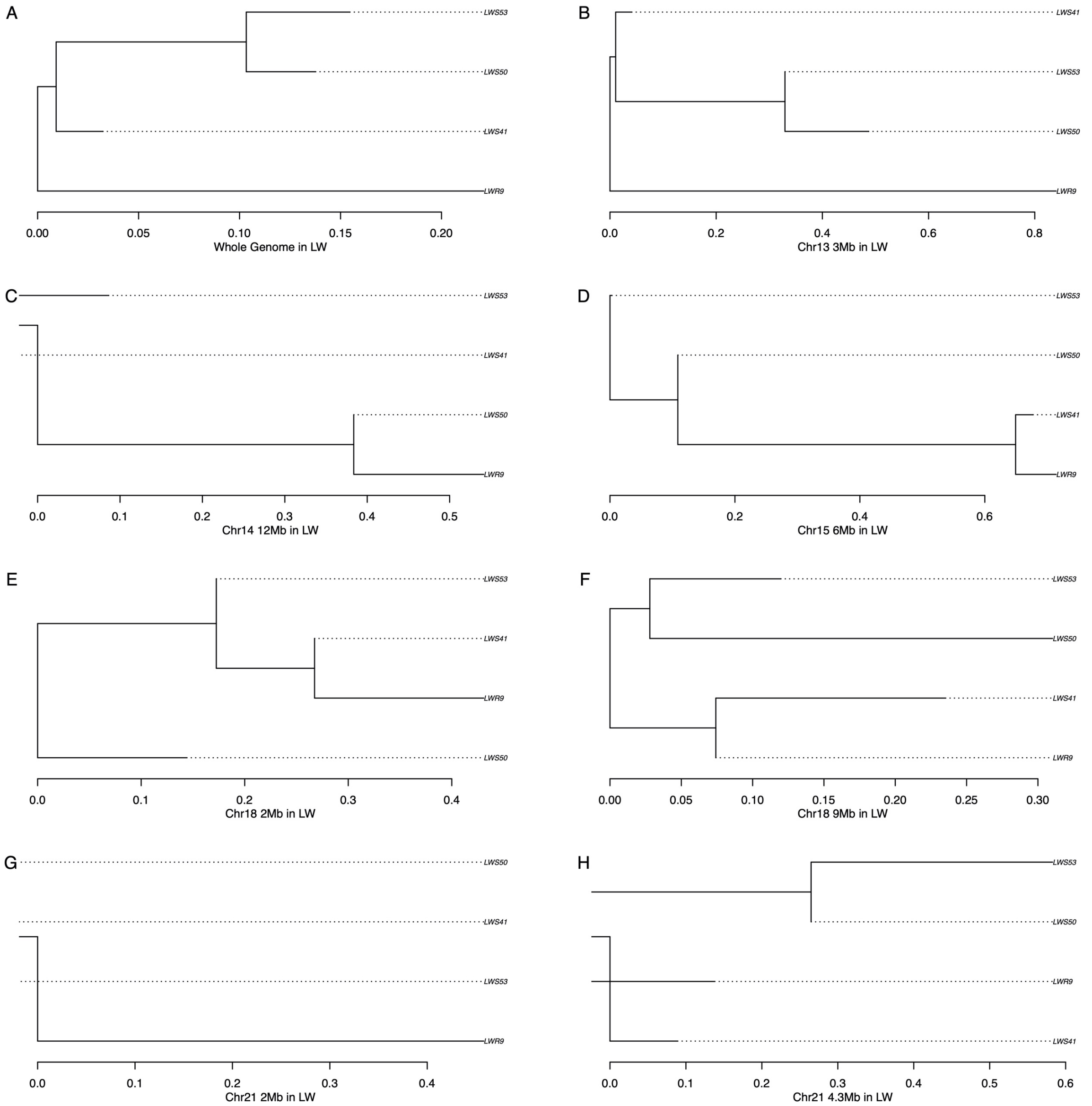

Figure S5. Hierarchical *hapFLK* trees for the LW lineage of the Virginia lines. **A** shows the tree constructed using whole-genome data for reference. **B-H** are the local trees constructed using genotype data for the *hapFLK* significant regions on chromosomes 13, 14, 15, 18, and 21.

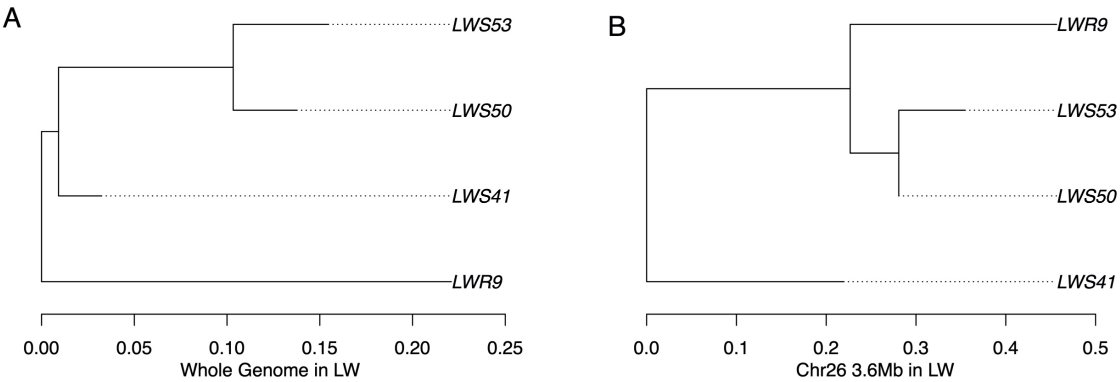

Figure S6. Hierarchical *hapFLK* trees for the LW lineage of the Virginia lines. **A** shows the tree constructed using whole-genome data for reference. **B** is the local tree constructed using genotype data for the *hapFLK* significant region on chromosome 26.

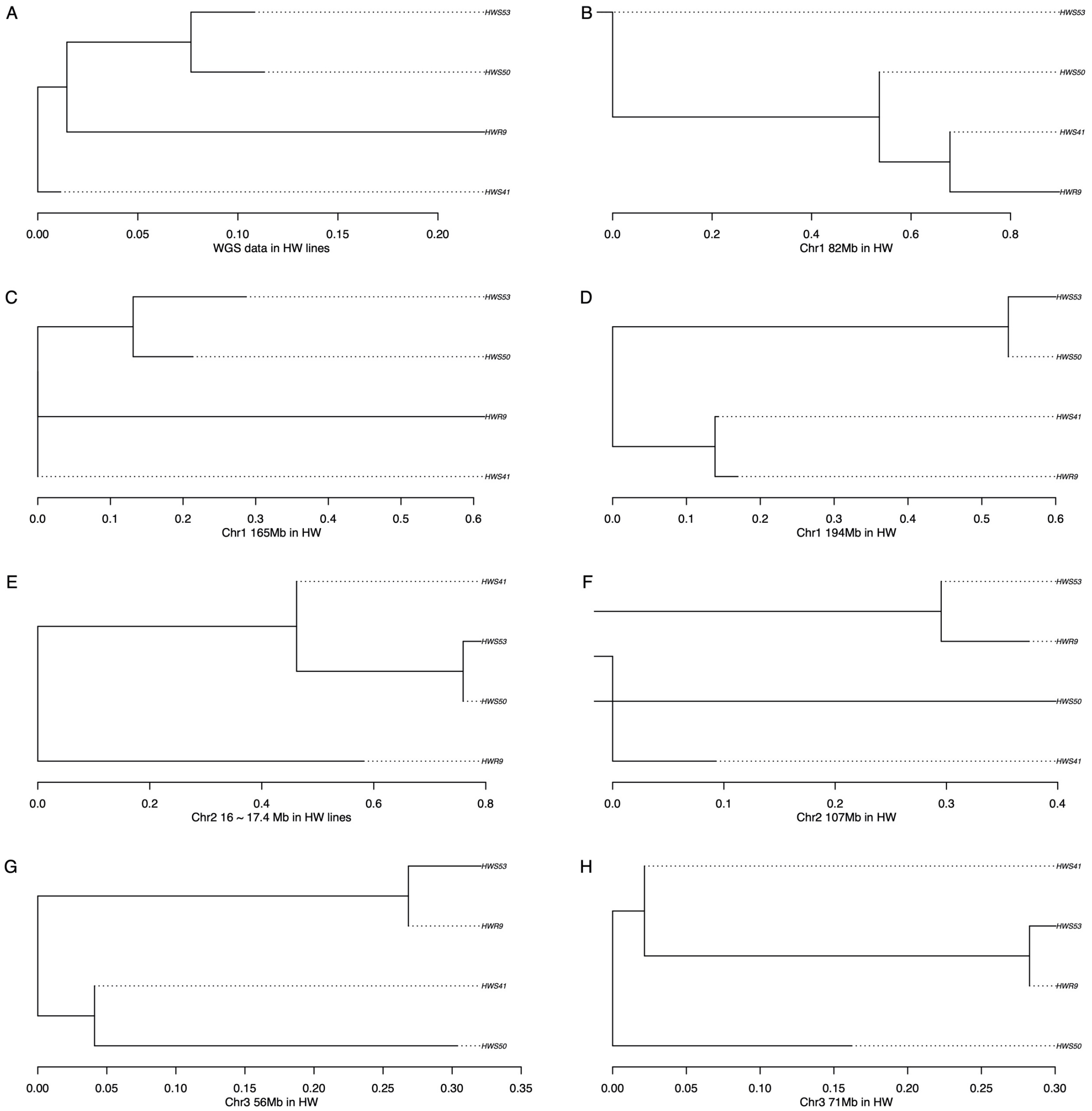

Figure S7. Hierarchical *hapFLK* trees for the HW lineage of the Virginia lines. **A** shows the tree constructed using whole-genome data for reference. **B**-**H** are the local trees constructed using genotype data for the *hapFLK* significant regions on chromosomes 1, 2, and 3.

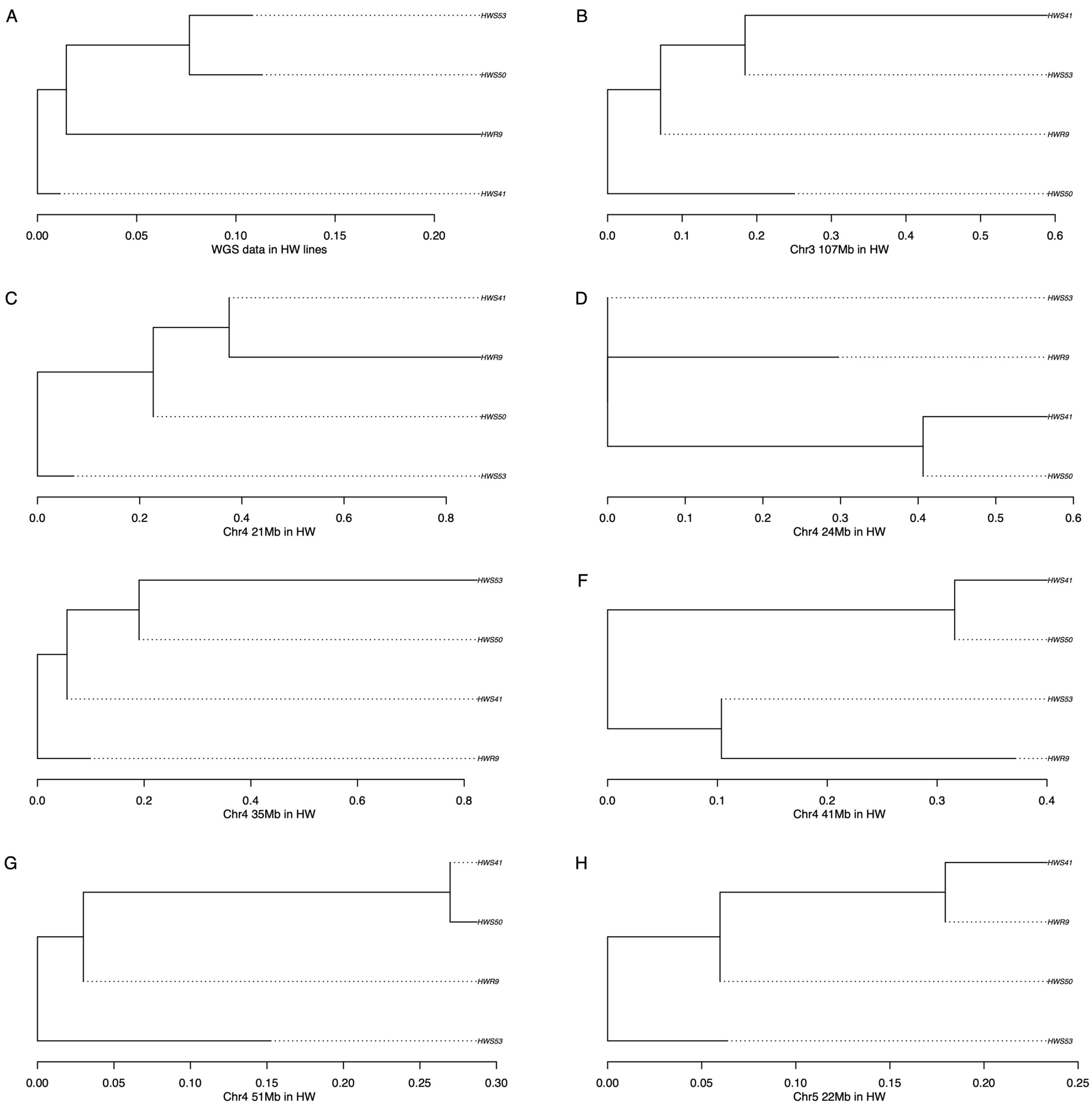

Figure S8. Hierarchical *hapFLK* trees for the HW lineage of the Virginia lines. **A** shows the tree constructed using whole-genome data for reference. **B**-**H** are the local trees constructed using genotype data for the *hapFLK* significant regions on chromosomes 3, 4, and 5.

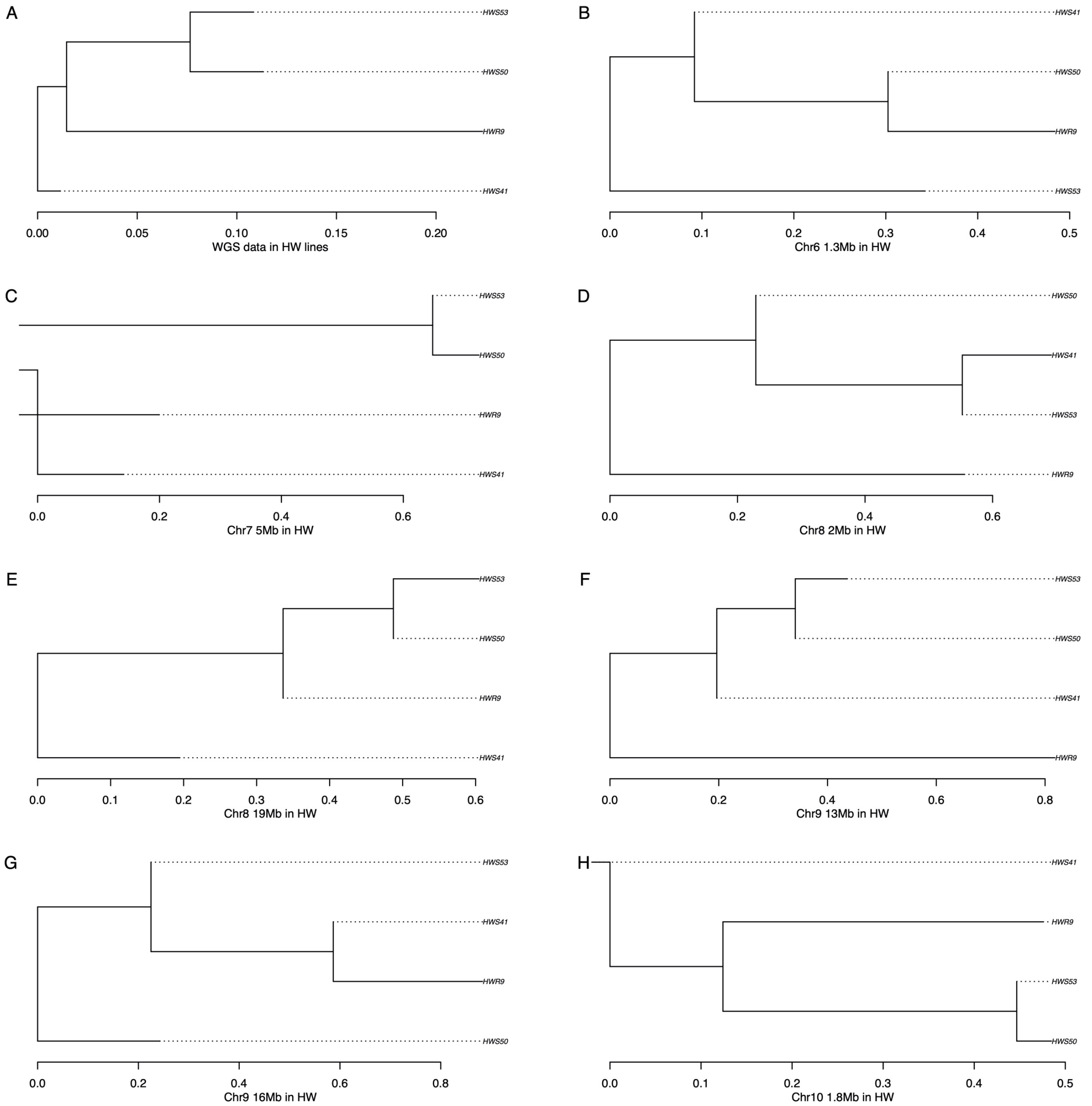

Figure S9. Hierarchical *hapFLK* trees for the HW lineage of the Virginia lines. **A** shows the tree constructed using whole-genome data for reference. **B**-**H** are the local trees constructed using genotype data for the *hapFLK* significant regions on chromosomes 6 – 10.

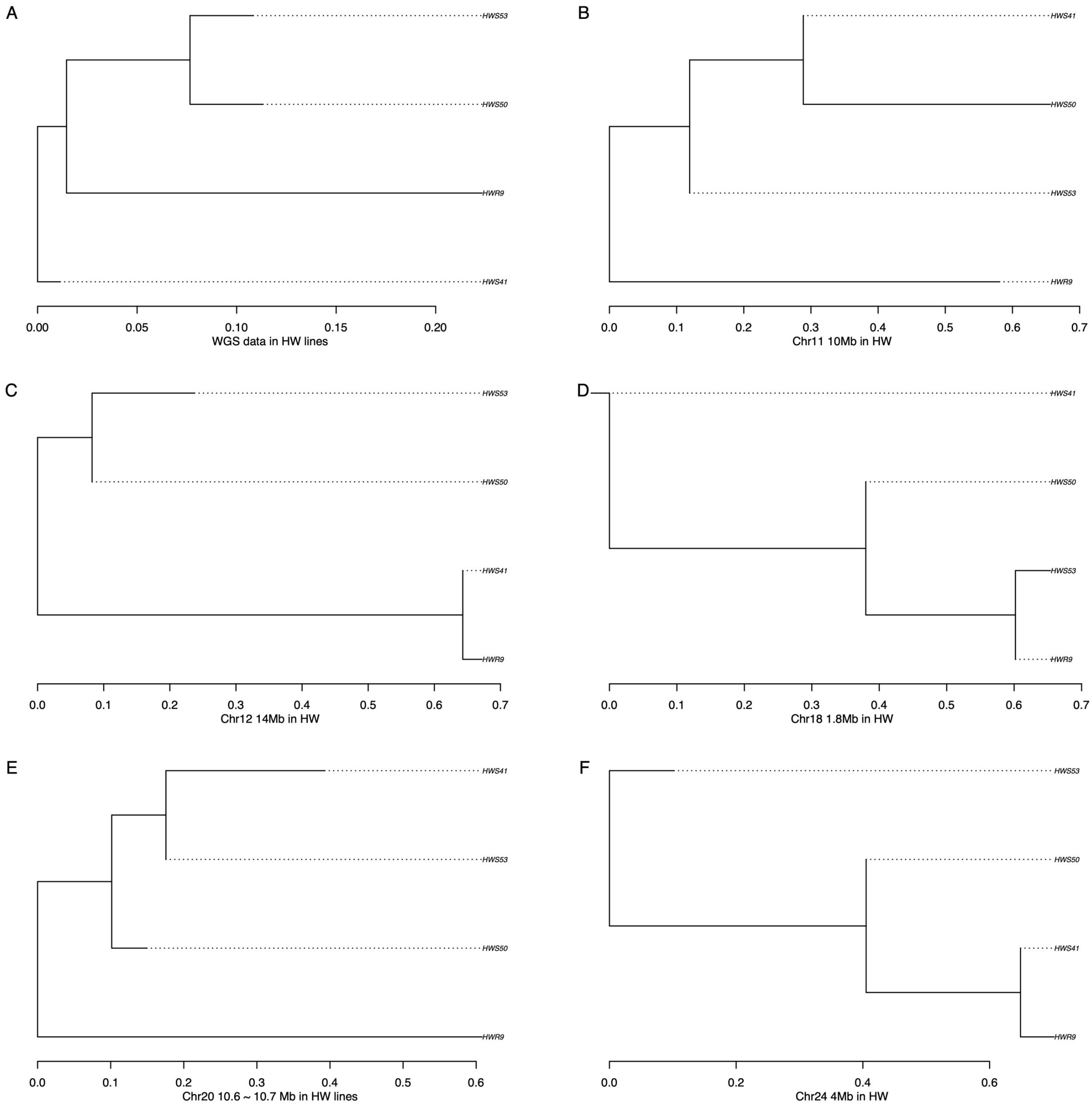

Figure S10. Hierarchical *hapFLK* trees for the HW lineage of the Virginia lines. **A** shows the tree constructed using whole-genome data for reference. **B**-**F** are the local trees constructed using genotype data for the *hapFLK* significant regions on chromosomes 11, 12, 18, 20, and 24.

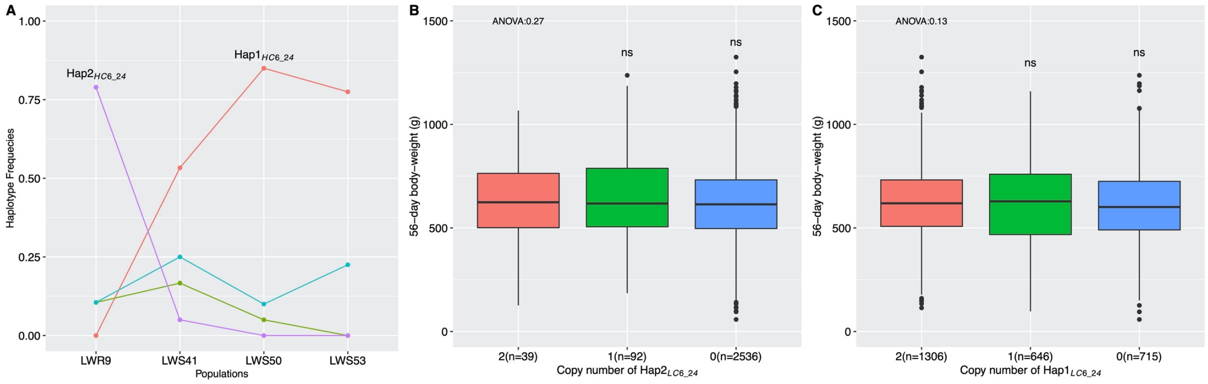

Figure S11. Changes in haplotype frequencies in the LW lineage (**A**) and associations between the major haplotypes in the relaxed (**B**) and selected (**C**) lines on chromosome 6 (~24Mb) in the AIL.

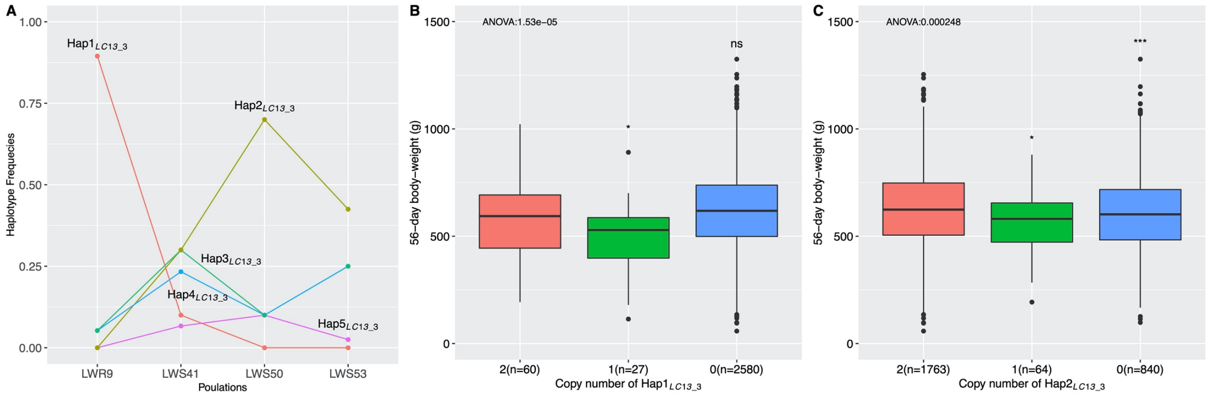

Figure S12. Changes in haplotype frequencies in the LW lineage (**A**) and associations between the major haplotypes in the relaxed (**B**) and selected (**C**) lines on chromosome 13 in the AIL.

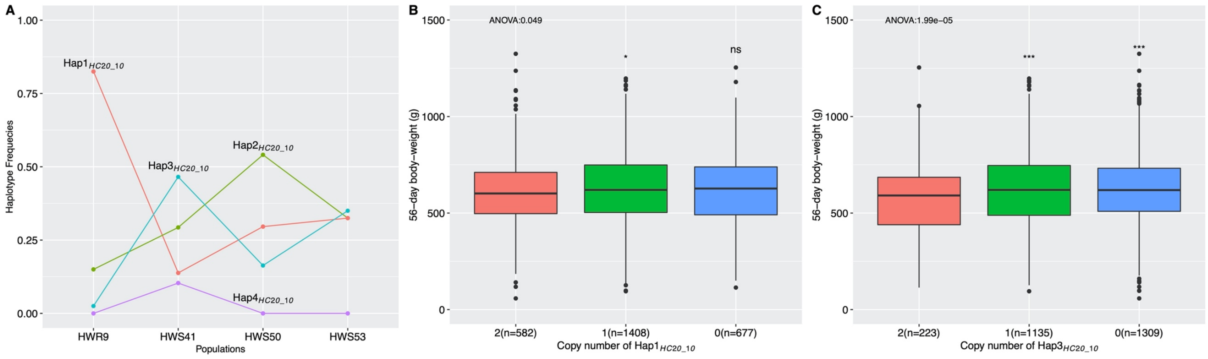

Figure S13. Association between the haplotype Hap3 detected on chromosome 20 in HW lineage in the AIL. Hap3 is the second most common selected haplotype and significantly related to body weight.

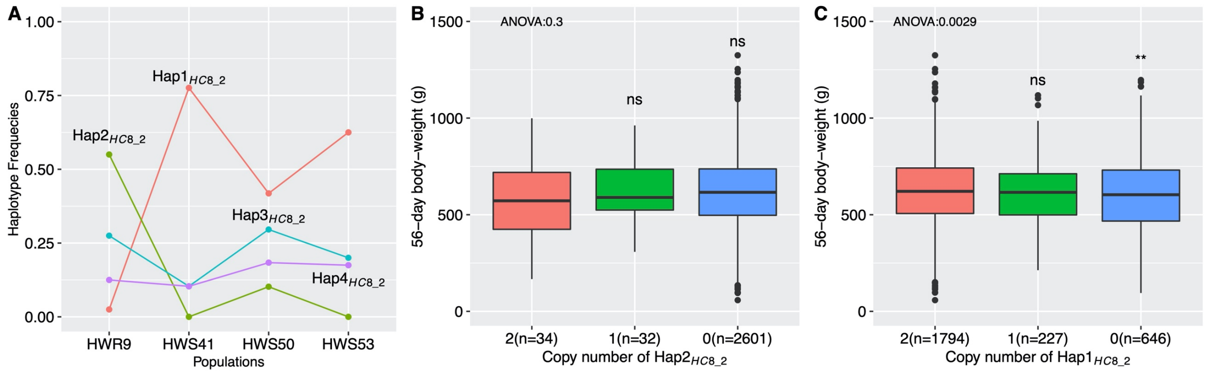

Figure S14. Association between the haplotype Hap2$_{HC8_2}$ on chromosome8 in HW lineage and 56-day body weight. Hap2$_{HC8_2}$ was the most common haplotype in relaxed lines but had no significant relation to 56-day body weight (B).

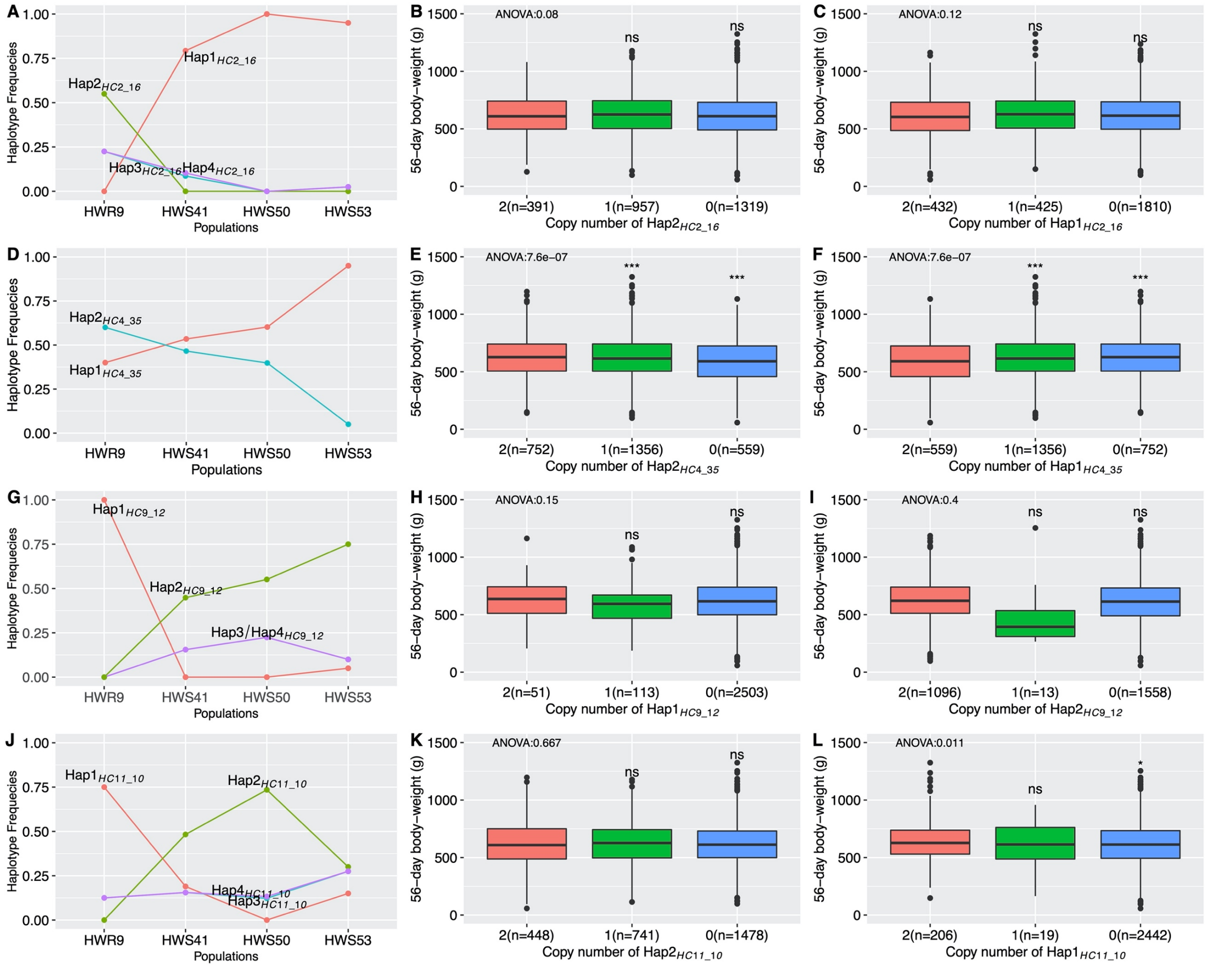

Figure S15. Changes in haplotype frequencies in the HW lineage **A/D/G/J** and associations between the major haplotypes in the relaxed **B/E/H/K** and selected **C/F/I/L** lines on chromosomes 2 **A-C**, chromosome 4 **D-F**, chromosome 9 **G-I** and chromosome 11 **J-L**.
